# New synthetic approaches for the construction of 2-aminophenoxazinone architectures

**DOI:** 10.1039/d5ra00604j

**Published:** 2025-03-28

**Authors:** Ning-Yu Guo, Xiao-Yi Cheng, Xiao-Dan Dong, Chun-e Peng, Chun Zhang, Ya-Ping Han, Li-Zeng Peng

**Affiliations:** a School of Chemical Engineering and Technology, Hebei University of Technology Tianjin 300130 China 2019070@hebut.edu.cn; b Key Laboratory of Agro-Products Processing Technology of Shandong Province, Key Laboratory of Novel Food Resources Processing, Ministry of Agriculture, Institute of Agro-Food Science and Technology, Shandong Academy of Agricultural Sciences Jinan China; c School of Pharmacy, Shandong University of Traditional Chinese Medicine Jinan China penglizeng@sdnu.edu.cn

## Abstract

Elaborated molecular architectures, specifically those containing a 2-aminophenoxazinone scaffold, belong to one of the most ubiquitous and prominent classes of heterocyclic frameworks, going from natural products to biologically active pharmaceutical molecules and from agrochemicals to functional materials and polymers. Therefore, efficient synthetic strategies for the assembly of 2-aminophenoxazinone frameworks are always in demand and have gained attention in academic and industrial communities. Methodologies that involve cascade reactions generally catalyzed by transition metal complexes, such as iron, cobalt, manganese, copper, and zinc complexes, have stood out as a representative approach. Over the past few decades, a great deal of versatile, atom-economic, and straightforward protocols have been reported for the generation of value-added 2-aminophenoxazinone frameworks in a sustainable, powerful, and applicable manner. The state-of-the-art methodologies toward the construction of 2-aminophenoxazinone skeletons are summarized in this review, which could be divided into four categories: (1) construction of 2-aminophenoxazinone compounds catalyzed by transition metal complexes; (2) construction of 2-aminophenoxazinone compounds catalyzed by biosynthetic enzymes; (3) synthetic process routes of 2-aminophenoxazinone compounds; and (4) construction of 2-aminophenoxazinone compounds *via* other innovative methods.

## Abstract 2

In this review, we summarize the recent advances in the synthesis of 2-aminophenoxazinone frameworks based on different catalytic approaches and synthetic process routes of 2-aminophenoxazinone compounds.

## Graphical abstract

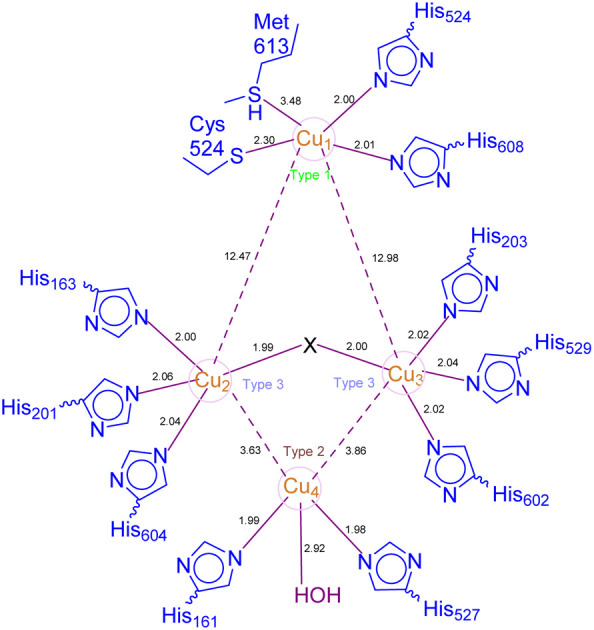

## 1. Introduction

The 2-aminophenoxazinone (APX) scaffold, frequently found in a wide range of natural metabolites, such as questiomycin, cinnabarinic acid, and actinomycin,^1–3^ has exhibited significant biological and pharmacological activities. Clinical success of some pharmaceutical molecules was extremely correlated with the 2-aminophenoxazinone scaffold, which could be employed as anti-inflammatory, anti-neoplastic, and antidiabetic compounds and were identified as practical clinical pharmaceutical agents for the treatment of cancer, bacterial infection, and other diseases.^4,5^ In addition, these alkaloids have been regarded as effective radical-trapping antioxidants, which could impede DNA-directed RNA synthesis by intercalating DNA binding between adjacent G–C base pairs in the double helix.^6^ In addition, the incorporation of a fragment of 2-aminophenoxazinone into the structure of particular dyes and fluorescent probes could make them applicable for live-cell imaging.^7^ Consequently, significant effort has been dedicated to the modularized generation of highly valuable, synthetically versatile, and architecturally complex 2-aminophenoxazinone scaffolds, which provided a wide range of highly functionalized and value-added compounds for streamlining the rapid identification of drug discovery.

**Fig. 1:**
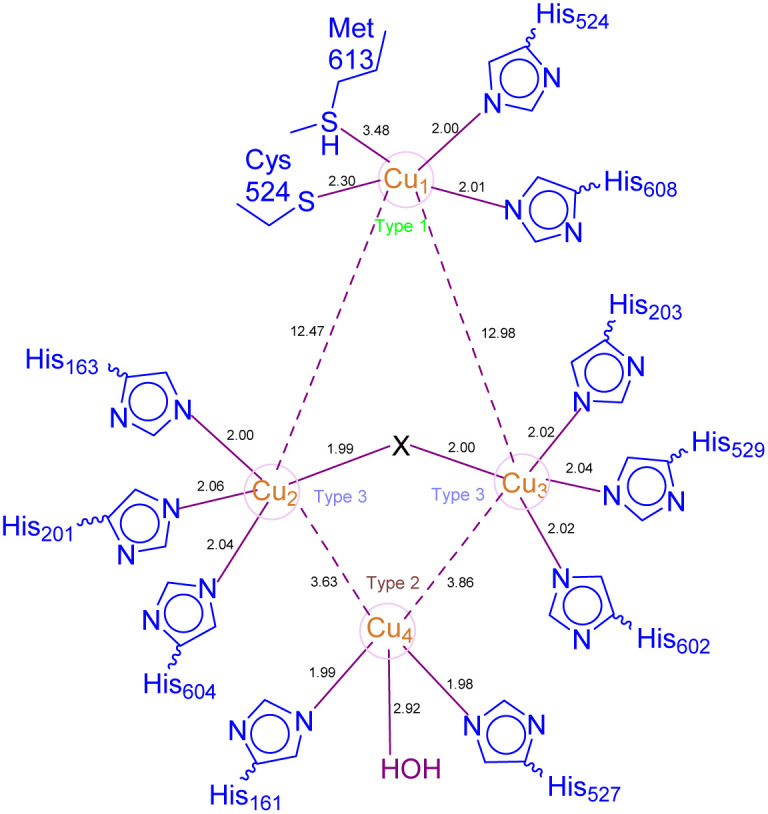
Schematic diagram of the phenoxazinone synthase enzyme; the letter ‘X’ stands for an unrecognized bridging ligand like OH.

In nature, the biosynthesis of APX scaffolds is facilitated by microorganisms through enzymatic reactions, and oxidative coupling processes catalyzed by phenoxazinone synthase (PHS) provide a highly efficient avenue to access architecturally sophisticated, synthetically versatile, and value-added APX scaffolds showing tunable selectivity and reactivity.^8,9^ The crystal structure of PHS was first isolated in 1962, which attracted a lot of attention due to its catalytic activity, and research into its mechanism for converting *o*-aminophenol (OAP) into APX commenced even prior to the complete elucidation of its structure.^10–12^ In 2006, it was reported that PHS existed as a hexamer with a pentanuclear copper core, which demonstrated superior activity compared to the dimeric form owing to its increased protein stability, enhanced accessibility to the active core, optimal metal center geometry, and the presence of solvent channels.^13^ The schematic illustration of four conserved copper atoms and their neighboring ligands are shown in Fig. 1, and the bonding and non-bonding distances are given in Å.

**Fig. 2:**
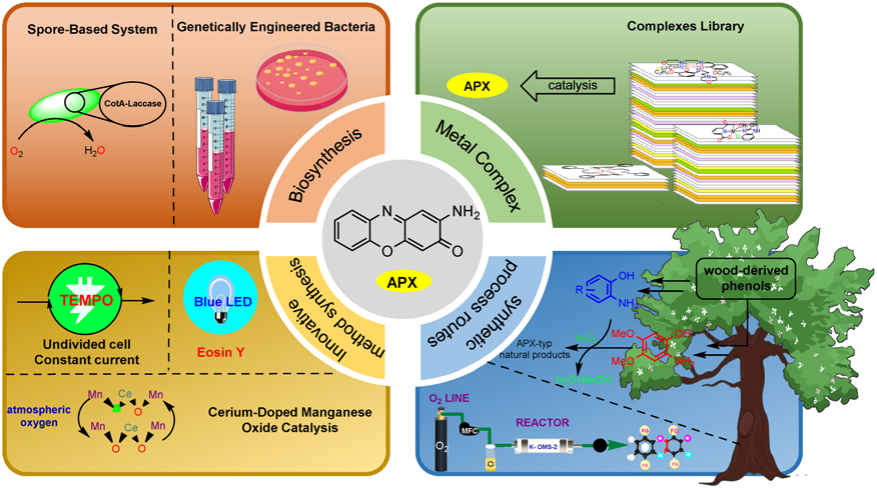
Recent advances in the synthesis of APX skeleton; the copyright permission of the picture was acknowledged in the Acknowledgements.

The elucidation of the PHS structure has significantly advanced the field of metalloenzyme-mediated synthesis of APX scaffolds and continued to inspire numerous researchers to collaboratively develop extensive libraries of complexes by investigating its active sites and catalytic mechanism.^14–18^ This ongoing research not only enhances our understanding of enzyme function but also promotes innovative catalyst design. Beyond metal enzyme-like pathways and specific environmental contexts, there is an increasing emphasis on improving product yields and conversion rates. Consequently, a significant number of researchers are exploring cleaner, more atomically economic, and stable synthetic strategies for the assembly of APX frameworks.^19–23^ Given the high biological and pharmacological values associated with APX frameworks, the development of their synthesis methods remains a prominent area. This review aims to summarize the synthesis methods of APX cores reported over the past few decades, categorizing advancements into four main chapters: transition metal complex-catalyzed reactions, biosynthetic enzyme-catalyzed reactions, synthetic process routes of 2-aminophenoxazinones, and other innovative methods (Fig. 2). This review seeks to provide valuable references and future research directions that will advance the field while developing more efficient and environmentally friendly synthetic strategies.

## 2. Construction of 2-aminophenoxazinones catalyzed by transition metal complexes

Modeling the active sites of metalloenzymes is a valuable approach that provides insights into the mechanistic pathway of natural enzymes, elucidates the roles of specific metals within these active sites, and facilitates the design of enhanced catalytic structures inspired by nature.^24^ Nevertheless, research on the mechanistic pathway of PHS remains relatively underdeveloped compared to more extensively studied enzymes such as catechol oxidase, indicating significant potential for advancement in this area.^8,25^ The PHS enzyme has been widely employed to simulate active sites and may possess mimetic capabilities that could surpass those found in biological enzyme models. This chapter summarizes various metal complexes exhibiting phenoxazinone synthase activity, which could be segmented into five sections according to their metal cores. It is anticipated that this examination will contribute to developing superior models and offer insights into the potential mechanism and pathway through utilizing these model complexes.

### 2.1 Cobalt complex-catalyzed reactions

**Scheme 1:**
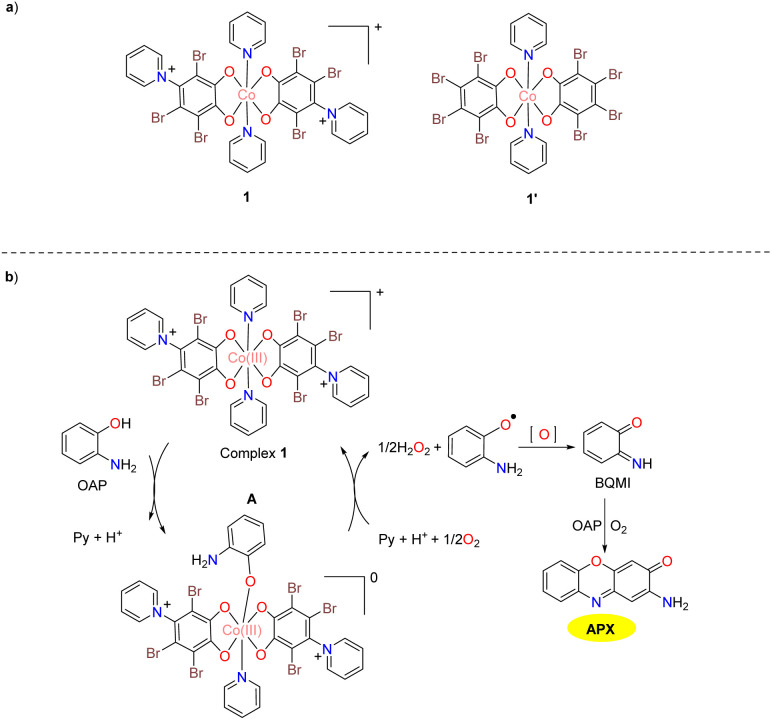
Structures of complexes 1 and 1′ and the proposed mechanism (ref. 26).

In 2018, the Panja group synthesized a mononuclear Co(iii) complex 1 at high temperatures by the reaction of CoCl_2_·6H_2_O with tetrabromophenol in excess pyridine (Scheme 1a).^26^ This complex exhibited moderate catalytic activity mimicking the function of phenoxazinone synthase. The bioinspired catalytic activity of complex 1 stimulated the investigation of compound 1′, which demonstrated negligible catalytic activity for reaction related to the phenoxazinone synthase function under the same conditions. The turnover number (*K*_cat_) value was calculated by dividing the maximum reaction rate (*V*_max_) by the concentration of the catalyst used, and it was found to be 30.6 h^−1^ for complex 1. The enhanced activity of complex 1 might be attributed to the two additional positive charges from the substituted pyridine ions, which act as anchoring entities. This mirrors a biological system where substrate recognition and intermediate stabilization through supramolecular interactions are key to metalloenzyme efficiency. The two pyridine ions in its ligand framework facilitate the formation of stable substrate–intermediate complexes before an irreversible redox transformation. The catalytic cycle for the generation of 2-aminophenoxazin-3-one begins with the formation of A, followed by a rate-determining oxidative breakdown of the intermediate to generate an OAP radical. Subsequently, the OAP radical is oxidized into *o*-benzoquinonemonoamine (BQMI), which could be further converted into the desired product 2-aminophenoxazin-3-one in the presence of oxygen and OAP (Scheme 1b).

**Scheme 2:**
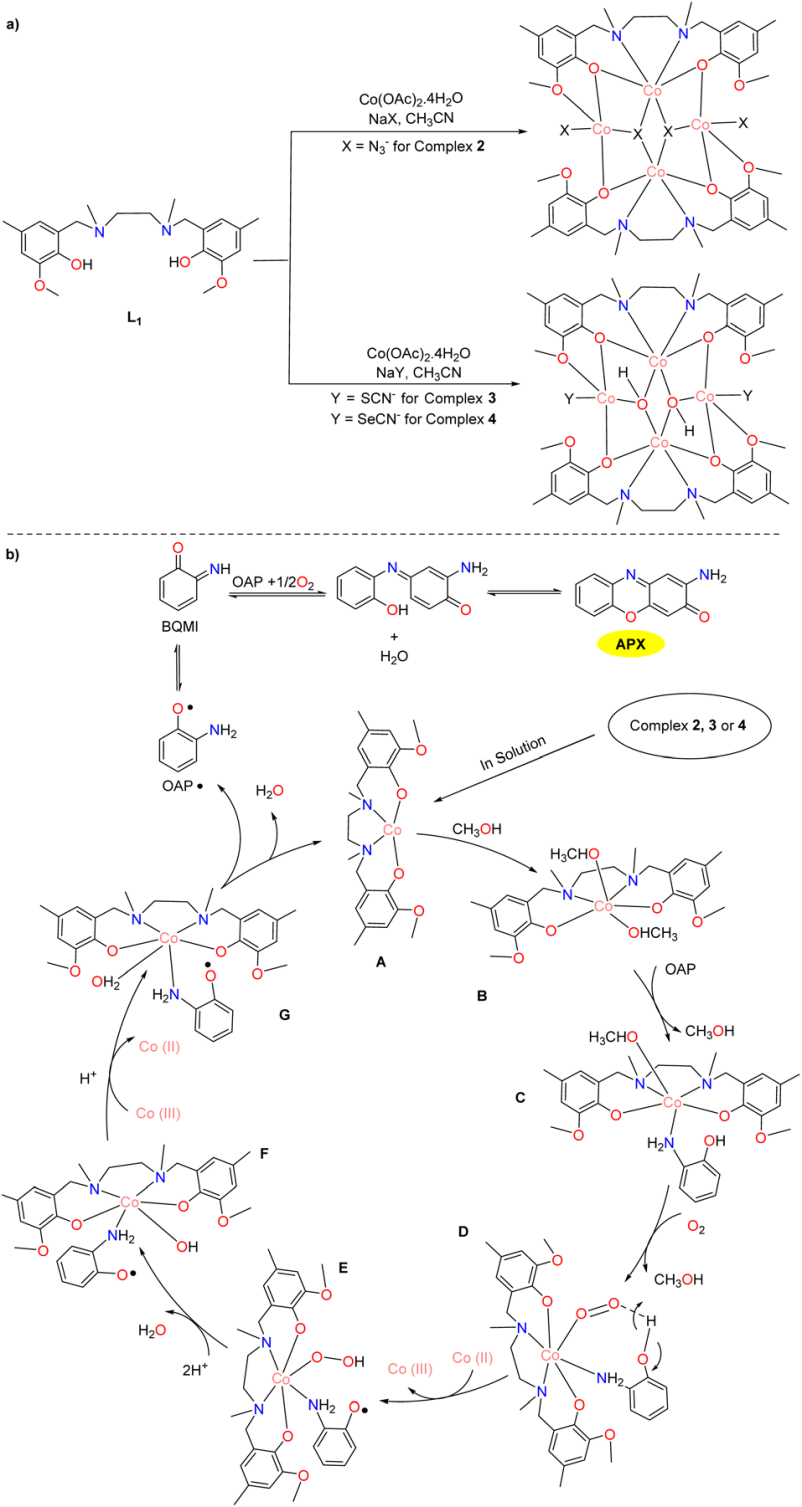
Synthesis of complexes 2–4 and the proposed mechanism (ref. 27).

In the same year, the Ghosh group synthesized three novel isomorphic tetranuclear Co(ii) complexes 2–4 with defective cubane nuclei (Scheme 2a).^27^ All three complexes showed a higher phenoxazinone synthase-like catalytic activity than most of the previously reported cobalt complexes. Of note, the *K*_cat_ value for the aerobic oxidation of OAP was calculated to be 500.4, 508.9, and 511.2 h^−1^ for complexes 2–4, respectively. The elaborate mechanism is depicted in Scheme 2b. The investigation of the catalytic mechanism employing these cobalt(ii) complexes reveals that the catalytic cycle is accomplished *via* a free radical process, entailing the oxidation of Co(ii) to Co(iii) with the formation of an OAP intermediate. Ultimately, the desired product is afforded through the oxidation of OAP species accompanying with water as a by-product and the regeneration of active cobalt complexes.

**Scheme 3:**
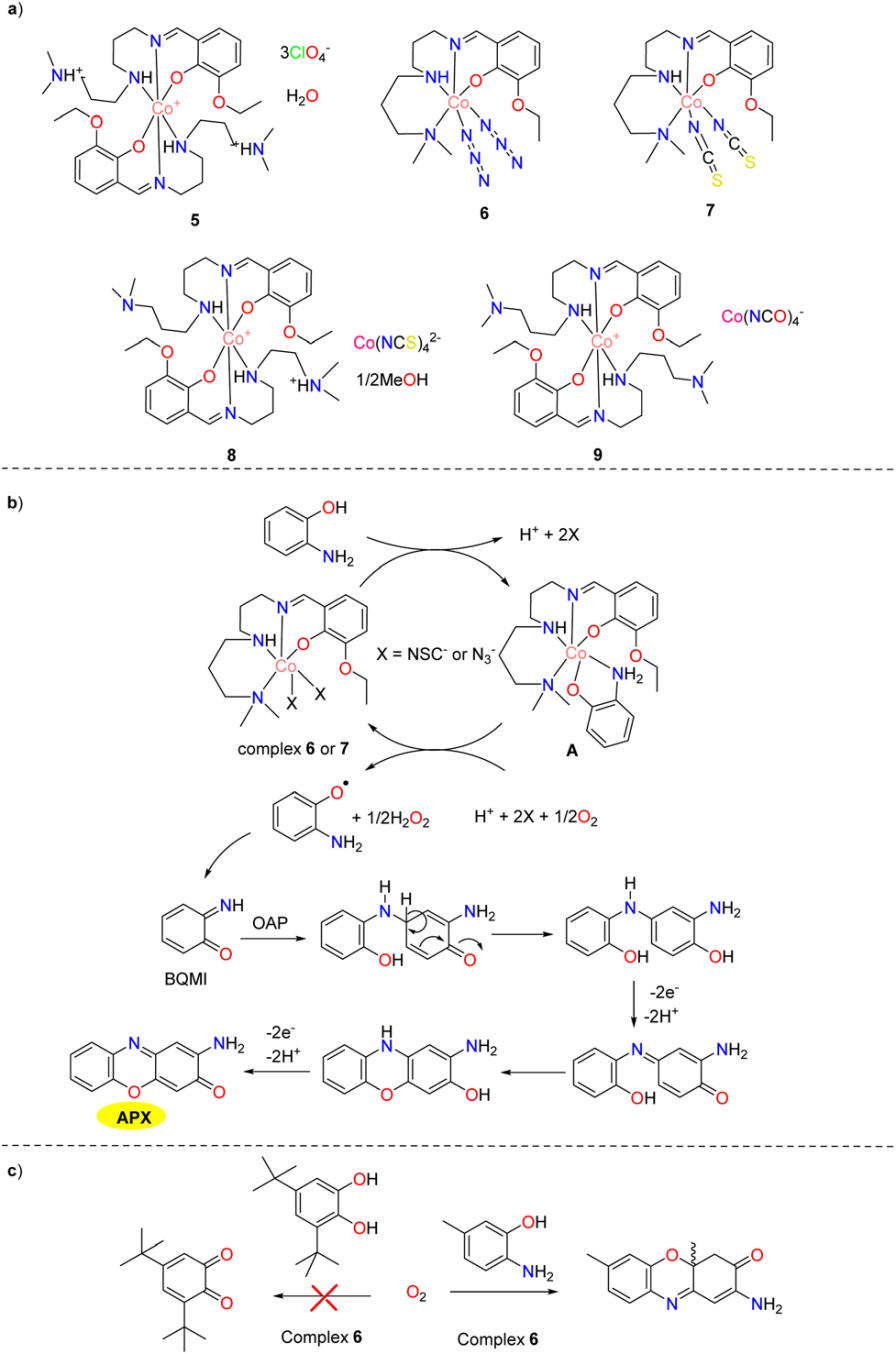
Structures of complexes 5–9 and the proposed mechanism (ref. 28).

In 2018, five novel cobalt complexes with different counterions were synthesized by the Panja group, all of which exhibited significant phenoxazinone synthase mimicry activity except for cobalt complex 5 (Scheme 3a).^28^ The *K*_cat_ value was found to be 4.35, 56.0, 53.8, 20.7 and 30.0 h^−1^ for complexes 5–9, respectively. The possible reaction mechanism is shown in Scheme 3b. The notable catalytic activity of cobalt complexes 6 and 7 was attributed to the binding sites in the metal center's first coordination sphere. While cobalt complex 5 showed minimal activity, the hydrogen bond acceptor sites in the second coordination spheres of cobalt complexes 8 and 9 made them effective for phenoxazinone synthase-like activity by stabilizing substrate aggregates and facilitating proton extraction. These results highlight the crucial role of vacant positions in the first coordination sphere for substrate binding, and the importance of substrate recognition and proton-extracting sites in the second coordination sphere for enhancing the catalyst performance *in vitro*. To demonstrate the synthetic practicality of the cobalt complex 6, the oxidation transformation of 3,5-di-*tert*-butyl catechol and 2-amino-5-methylphenol were carried out in the presence of cobalt complex 6 employing oxygen as the oxidizing agent. It is worth noting that complex 6 could efficiently catalyze the aerobic oxidation of 2-amino-5-methylphenol, but is ineffective for oxidizing 3,5-di-*tert*-butyl catechol (Scheme 3c).

**Scheme 4:**
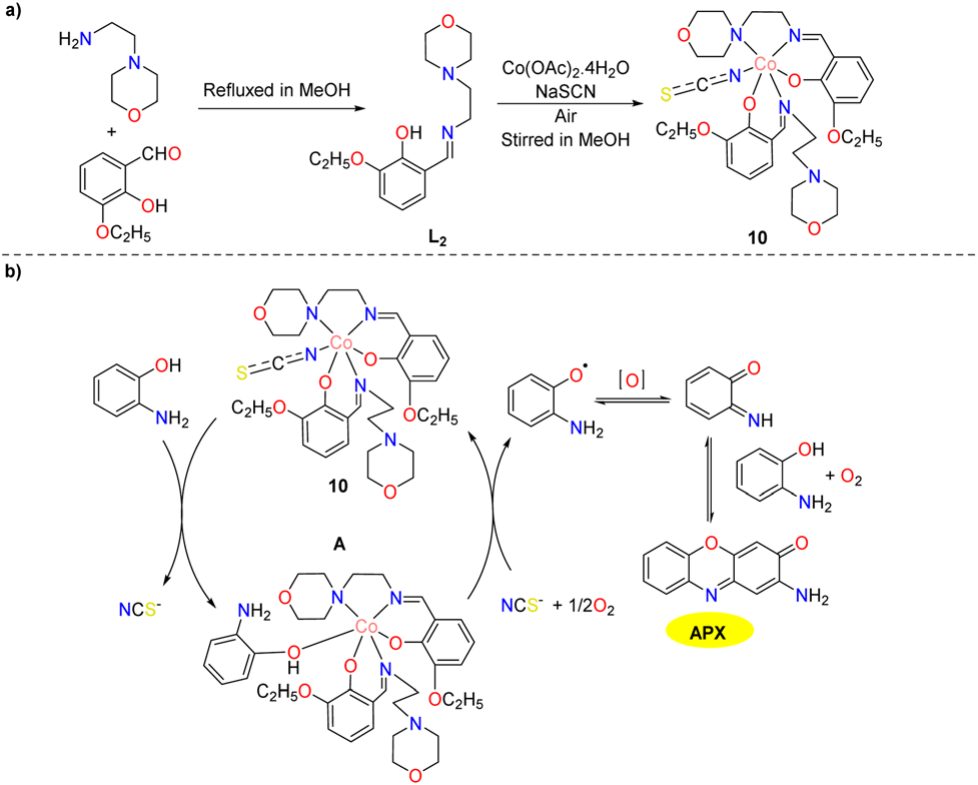
Synthesis of complex 10 and the proposed mechanism (ref. 29).

In the same year, a cobalt(iii) complex featuring a pendant Schiff base ligand was synthesized and characterized by Chattopadhyay and co-workers (Scheme 4a).^29^ The solid-state structure of the complex reveals that the organic ligand participates in diverse supramolecular interactions, encompassing interactions with ethoxy arms and aromatic rings. The complex demonstrates catalytic activity towards mimicking phenoxazinone synthase and the *K*_cat_ value is 2.059 × 10^−2^ s^−1^ in the Michaelis–Menten plot. A tentative mechanism for the generation of APX framework is outlined in Scheme 4b. In the initial stage, the substrate coordinates with the complex and substitutes the monodentate thiocyanate co-ligand. This complex–substrate intermediate undergoes degradation in the rate-determining step, resulting in the generation of a substrate radical. Subsequently, the radical yields the phenoxazinone chromophore in the presence of molecular dioxygen through a series of oxidative dehydrogenation processes to deliver the desired product.

**Scheme 5:**
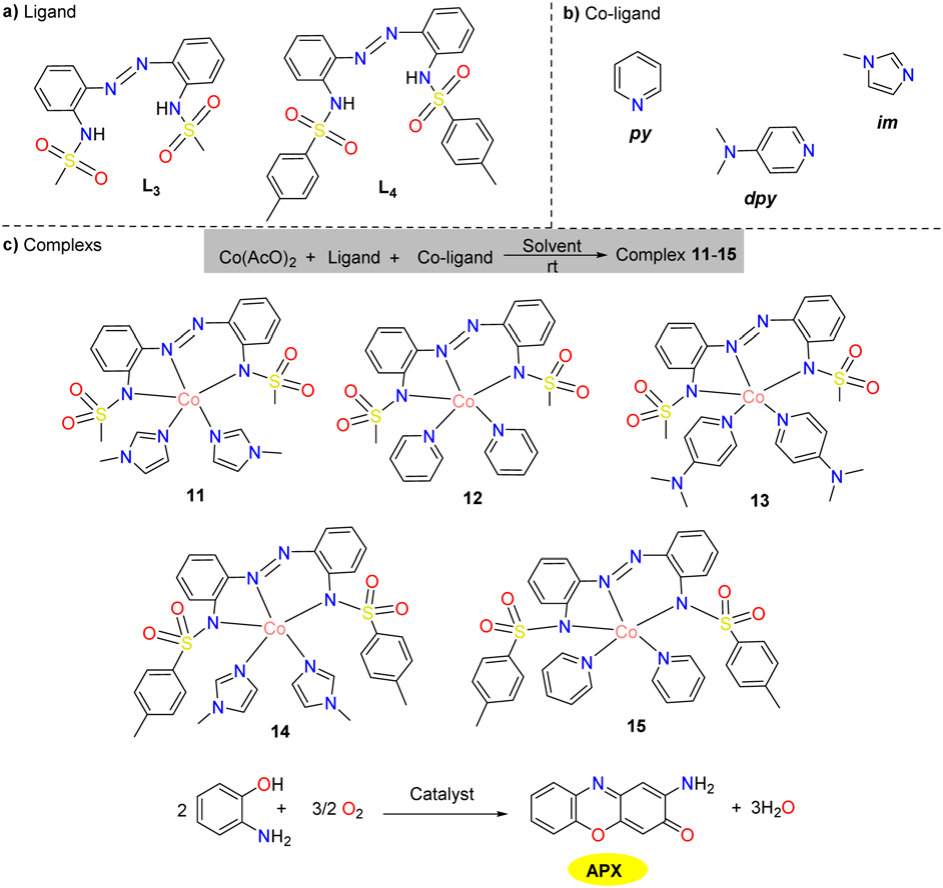
(a) Synthesized diazene–disulfonamide chelate ligands. (b) Neutral monodentate co-ligands. (c) Compositions of cobalt(ii) complexes 11–15 (ref. 30).

In 2019, two novel diazene disulfonamide ligands were combined and assembled with monodentate nitrogen-donor co-ligands derived from imidazole or pyridine moieties by the Eseola group to afford well-characterized cobalt(ii) complexes 11–15 (Scheme 5).^30^ All these cobalt(ii) complexes demonstrated good phenoxazinone synthase mimetic activity. Furthermore, the research has conducted an in-depth exploration of the correlation between these complexes' catalytic efficiency and spatial characteristics, which effectively filled the gap in the systematic methods for studying the structure–property correlation of complexes for the synthesis model of phenoxazinone. The experiment employs the initial rate method to compare reaction rates. The initial rates for complexes 11–15 were 2.58 × 10^−3^, 7.19 × 10^−3^, 11.30 × 10^−3^, 1.02 × 10^−3^, and 1.30 × 10^−3^ h^−1^, respectively. The results indicate that as the steric hindrance of ligands L_3_ and L_4_ decreases, the enzyme activity increases. Additionally, a correlation exists between the catalytic efficiency and the facility of co-ligand dissociation, which might be ascribed to the increasing size of the co-ligand following the trend of dpy > py > im. These findings corroborated that the involved metal complexes exhibited good site-selective reactivities due to the unhindered availability of unoccupied metal coordination sites devoid of spatial constraints, serving as a pre-requisite for the pronounced responsiveness exhibited by metalloenzymes during APX synthesis.

**Scheme 6:**
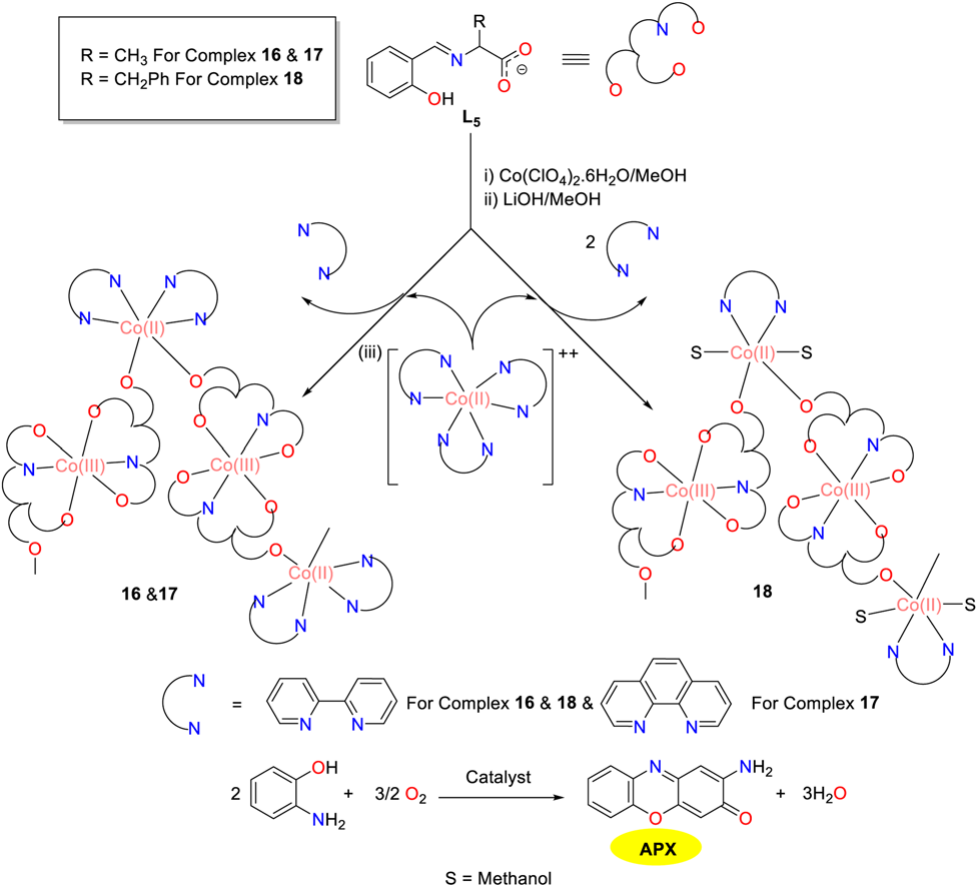
Synthetic route to complexes 16–18 and the oxidation reaction of OAP to APX (ref. 31).

In the same year, the “metal–ligand” strategy was utilized by the Ghosh group to synthesize and structurally characterize three novel mixed-valence 1D chains featuring alternating Co(ii) and Co(iii) units (Scheme 6).^31^ These chains were synthesized employing a tridentate Schiff base derived from l-alanine/l-phenylalanine and salicylaldehyde, along with neutral *N*,*N*-donor chelating bpy/phen ligands. In addition, the Co(ii) and Co(iii) centers were connected by a μ_1,3_*syn*–*anti* carboxylate bridge in cobalt complexes 16–18. The oxidation of *o*-aminophenol to phenoxazinone was catalyzed by those three cobalt complexes, representing the first example of mixed-valence Co(ii)/Co(iii) coordination polymers exhibiting the phenoxazinone synthase activity. The *K*_cat_ values of complex 16–18 were 1.2, 11.5, and 2.7 h^−1^, respectively. The significantly higher *K*_cat_ value observed for complex 17 might be attributed to the enhanced π-acceptor properties of the phen ligand, which stabilized the reaction intermediates more effectively than the bpy ligand presented in complexes 16 and 18. Mechanistically, it is apparent that in all three cobalt complexes, the Co(ii) center binds *o*-aminophenol while Co(iii) undergoes redox activity, highlighting the cooperative effect of the metal centers in different oxidation states.

**Scheme 7:**
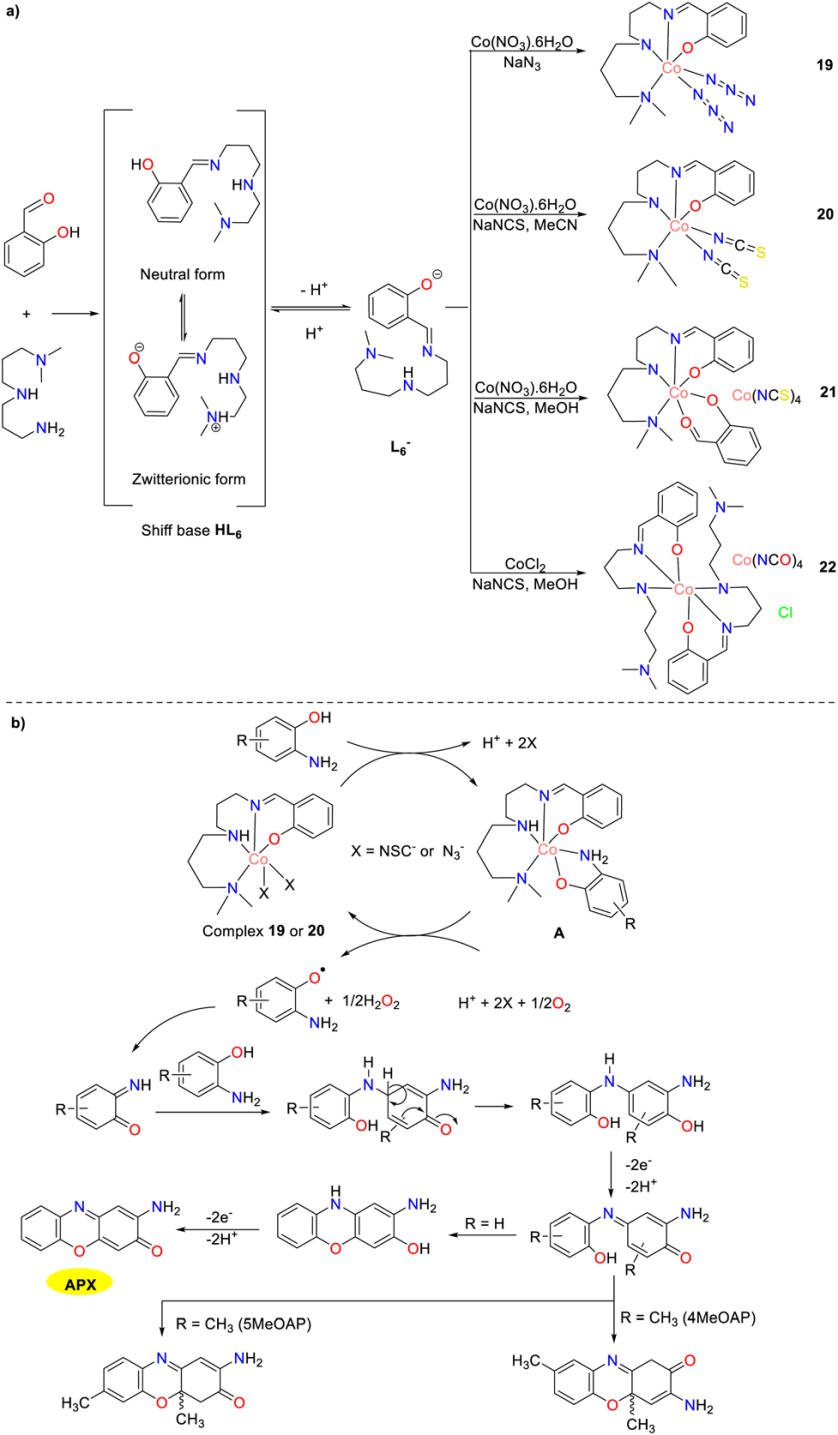
Synthesis of complexes 19–22 and the proposed mechanism (ref. 32).

In 2019, the Panja team synthesized four cobalt complexes 19–22 by the reaction of tetradentate Schiff base derived from *N*,*N*-dimethyldipropylenetriamine, salicylaldehyde, and cobalt(ii) salts in the presence of various pseudohalide ions (Scheme 7).^32^ All synthesized cobalt complexes exhibited catalytic activity in the aerobic oxidation of various OAPs. For complexes 19–22, the *K*_cat_ values for the aerobic oxidation of OAP were measured at 47.36, 47.60, 16.46, and 10.34 h^−1^, respectively. In contrast, for complexes 19 and 20, the *K*_cat_ values for the aerobic oxidation of 2-amino-5-methylphenol (5MeOAP) were found to be 74.38 and 77.85 h^−1^, while those for the aerobic oxidation of 2-amino-4-methylphenol (4MeOAP) were recorded as 71.94 and 79.66 h^−1^, respectively. Notably, complexes 19 and 20 showed high reactivity due to the presence of substitutionally labile metal-bound pseudohalide ions, which could facilitate substrate binding. The methyl-substituted 4MeOAP and 5MeOAP were significantly more favorable than the parent OAP, which might be attributed to the electron-donating effect of the methyl group. Mass spectrometric analysis revealed that the methyl substituent did not prevent the formation of a stable complex–substrate intermediate during the catalytic cycle, and it did inhibit the final oxidative dehydrogenation step. This inhibition led to the formation of a dihydro-phenoxazinone chromophore rather than a phenoxazinone chromophore.

**Scheme 8:**
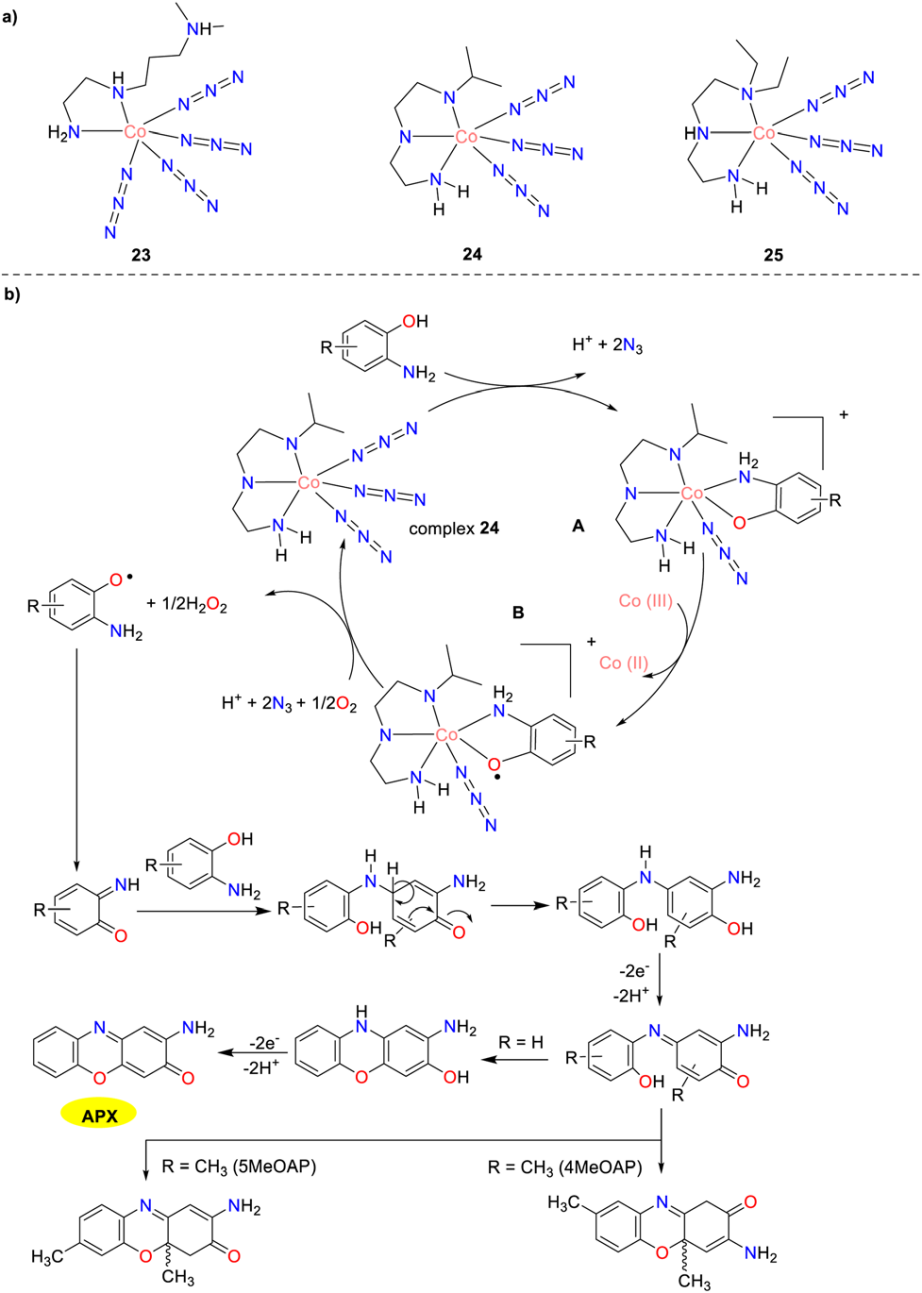
Structures of complexes 23–25 and the proposed mechanism (ref. 33).

In 2020, three new azide-bound cobalt(iii) complexes 23–25 were synthesized and characterized by Jana and co-workers as depicted in Scheme 8.^33^ The *K*_cat_ values for the aerobic oxidation of OAP by the complexes were 61.92, 40.34, and 42.63 h^−1^, respectively. For the aerobic oxidation of 5MeOAP, the corresponding *K*_cat_ values were 112.12, 89.35, and 87.81 h^−1^, whereas, for the aerobic oxidation of 4MeOAP, the *K*_cat_ values were 83.59, 65.61, and 60.12 h^−1^, respectively. These cobalt complexes exhibit high phenoxazinone synthase activity attributed to the presence of substitutionally labile azide ions in the metal coordination sphere. Cobalt complex 23 contains four labile azide ions, while cobalt complexes 24 and 25 have three azide ions each around the cobalt(iii) center. Consequently, complex 23 was anticipated to have a higher probability for the formation of the complex–substrate intermediate than complexes 24 and 25, making it the superior catalyst in this series. Notably, complexes 24 and 25 demonstrated similar activity in the catalytic oxidation of *o*-aminophenol, likely due to their structural similarity. Nevertheless, for *o*-aminophenol substituted with a methyl group, the final oxidative dehydrogenation step was hindered, leading to the formation of dihydro-phenoxazinone chromophore instead of phenoxazinone.

**Scheme 9:**
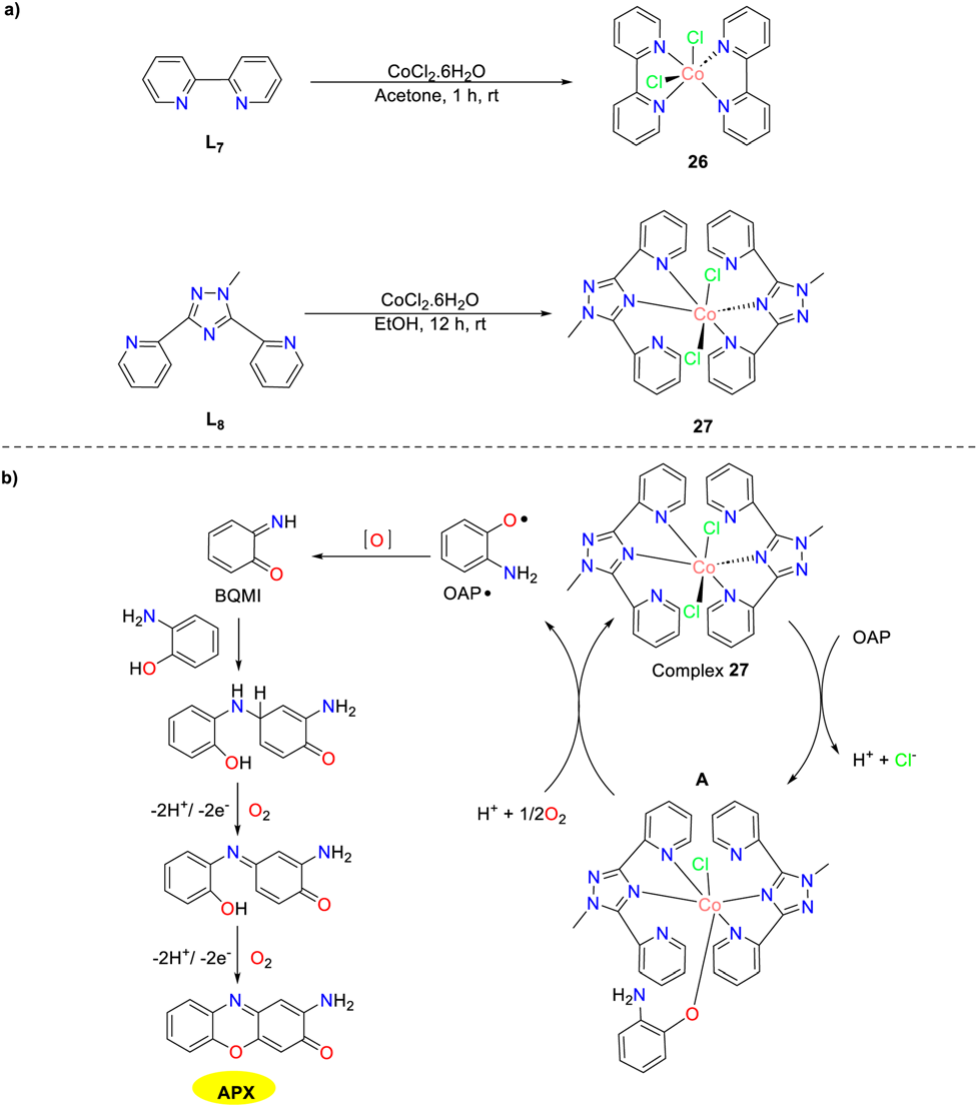
Synthesis of complexes 26–27 and the proposed mechanism (ref. 34).

In 2022, the Maji group synthesized two *cis*-dichlorocobalt(ii) mononuclear complexes 26 and 27 with polypyridine ligands (Scheme 9).^34^ Polypyridyl ligands were extensively used in a homogeneous catalytic system for organic and biological processes. It is worth noting that ligand 7 was modified by incorporating a triazole unit between its pyridine rings, leading to the formation of ligand 8 to prevent undesired metal coordination. Both complexes 26 and 27 exhibited significant phenoxazinone synthesis activity with *K*_cat_ values of 201.24 h^−1^ and 249.57 h^−1^, respectively. A tentative mechanism for the generation of APX scaffolds is outlined. The substrate undergoes internal electron transfer under aerobic conditions and generates OAP radicals, which is further converted into a BQMI intermediate through radical disproportionation in the rate-determining step. Subsequently, the reaction of the BQMI intermediate with another OAP furnishes diphenylamine species, followed by multi-electron oxidation to afford APX products.

**Scheme 10:**
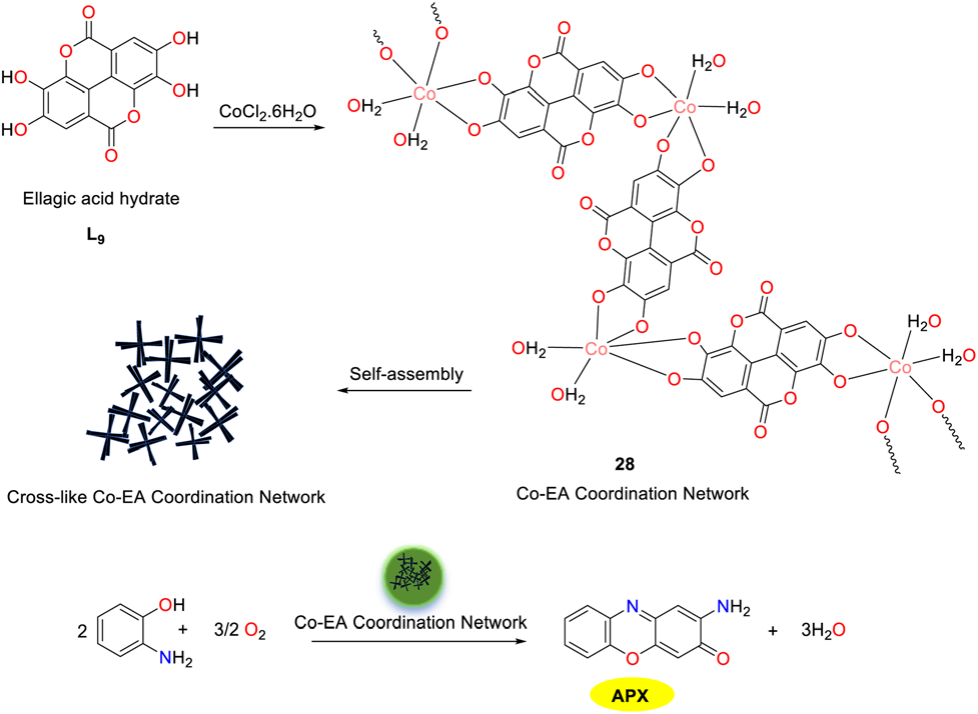
Preparation of complex 28 and the oxidation reaction of OAP to APX (ref. 35).

A distinctive cross-shaped metal–polyphenol component 28 was prepared by the Sarma group in 2023, utilizing naturally available polyphenol ellagic acid (EA) and Co(ii) ions as the substrates in aqueous media under ambient conditions (Scheme 10).^35^ Extensive coordination with two catechol groups from distinct ellagic acid was achieved by the high-spin Co(ii) centers in an octahedral environment, where adjacent vacancies were occupied by two water molecules, leading to the formation of Co_2_(CH_2_O_2_)_2_ as a monomer unit. The redox activity was significantly enhanced by the Co(ii) centers within the network, enabling the Co–EA catalyst to simulate the activities of phenoxazinone synthase, laccase, and oxidase. The heterogeneous nature of the Co–EA was validated by its stability and improved recyclability during the phenoxazinone synthase reaction, the efficient degradation of a series of phenolic substrates was accomplished, and APX scaffolds were synthesized by oxidizing OAP with the Co–EA catalyst. The Michaelis–Menten constant (*K*_M_ value) of complex 28 (1.3 × 10^−3^ M) was compared with other reported phenoxazinone synthase mimic systems. The lower *K*_M_ value indicates that the catalyst has a higher affinity for the substrate. Given its cost-effective synthesis, robust activity, stability, and recyclability, this polyphenol-based functional material was considered promising as a heterogeneous catalyst in organic transformation and environmental remediation.

**Scheme 11:**
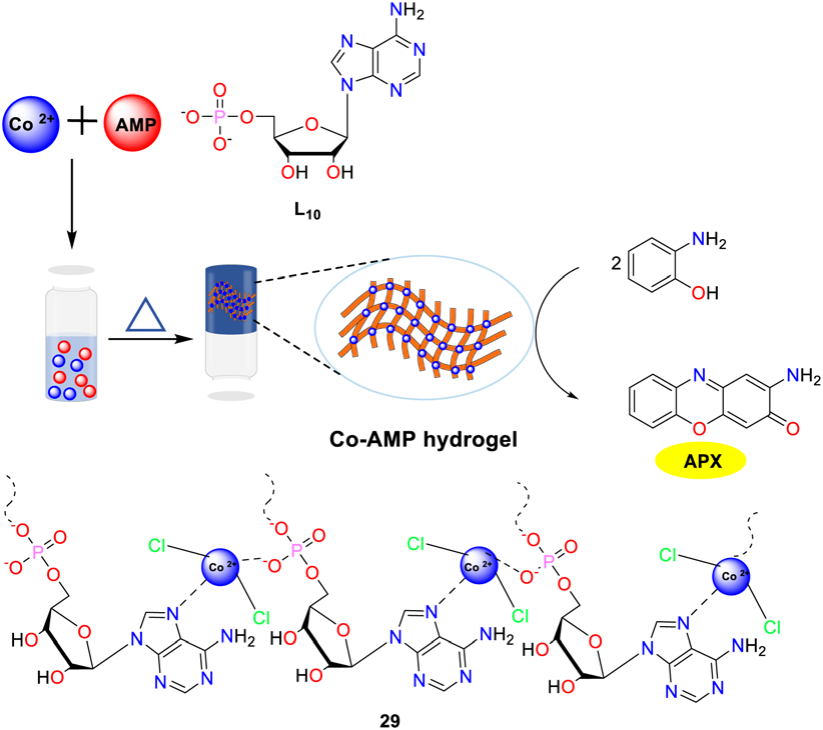
Schematic presentation of the synthesis of the Co-AMP hydrogel (ref. 36).

In the same year, the Sarma group reported a heat-sensitive metallogel, which was synthesized spontaneously through the self-assembly of adenosine 5′-monophosphate (AMP) and cobalt chloride, and exhibited a color transition indicative of a structural shift from an octahedral to a tetrahedral configuration upon heating (Scheme 11).^36^ This metallogel, demonstrating exceptional stability across a pH range of 1 to 12, is being developed as a multifunctional enzyme mimic. It has been shown to exhibit pH-responsive catalase and peroxidase activities, with catalase-like activity occurring under neutral and basic conditions and peroxidase-like activity manifesting in an acidic environment. Additionally, the versatility of the metallogel was further demonstrated by its phenoxazinone synthase-like activity. The activity was evaluated by employing OAP as the substrate. The *K*_M_ and *V*_max_ values were calculated to be 0.005 and 0.016 M min^−1^, respectively.

**Scheme 12:**
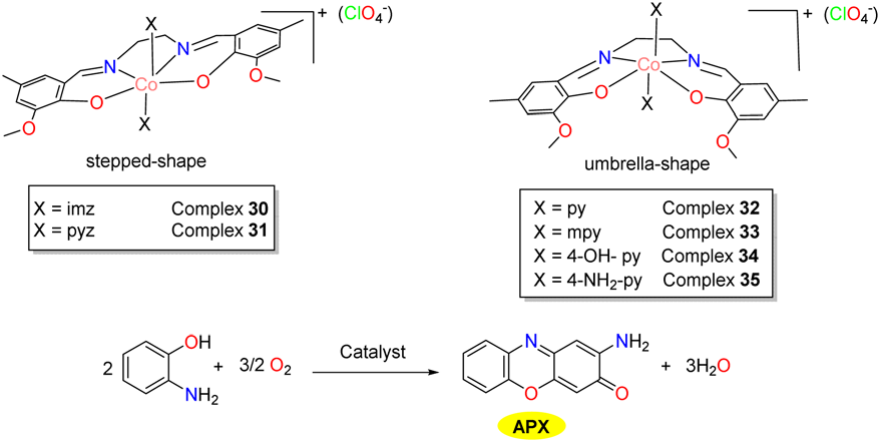
Complexes 30–35 involving cobalt(ii) salts with various heteroaromatic co-ligands, and the oxidation of OAP to APX (ref. 37).

In 2024, six mononuclear cobalt(iii) complexes 30–35 were synthesized and characterized by the Panja group for investigating the effect of heteroaromatic co-ligands on the conformational selectivity (stepped *versus* umbrella) of these complexes (Scheme 12).^37^ Notably, despite various intermolecular non-covalent interactions in the solid state, pyridyl co-ligands exclusively stabilize the umbrella-shaped conformation 32–35 while five-membered heteroaromatic bases stabilize the stepped conformation 30–31, which indicates that the different degrees of intramolecular interactions of six-membered *versus* five-membered heteroaromatic ligands at the axial position are crucial for the selective stability of these conformations in the solid state. All these complexes exhibited high catalytic activity, mimicking that of phenoxazinone synthase. The *K*_cat_ value fell in the range of 29.07–41.38 h^−1^ for these complexes. The slightly lower activity of cobalt complexes 30 and 31 compared to others could be explained by the lower basicity of pyrazole.

### 2.2 Iron complex-catalyzed reactions

**Scheme 13:**
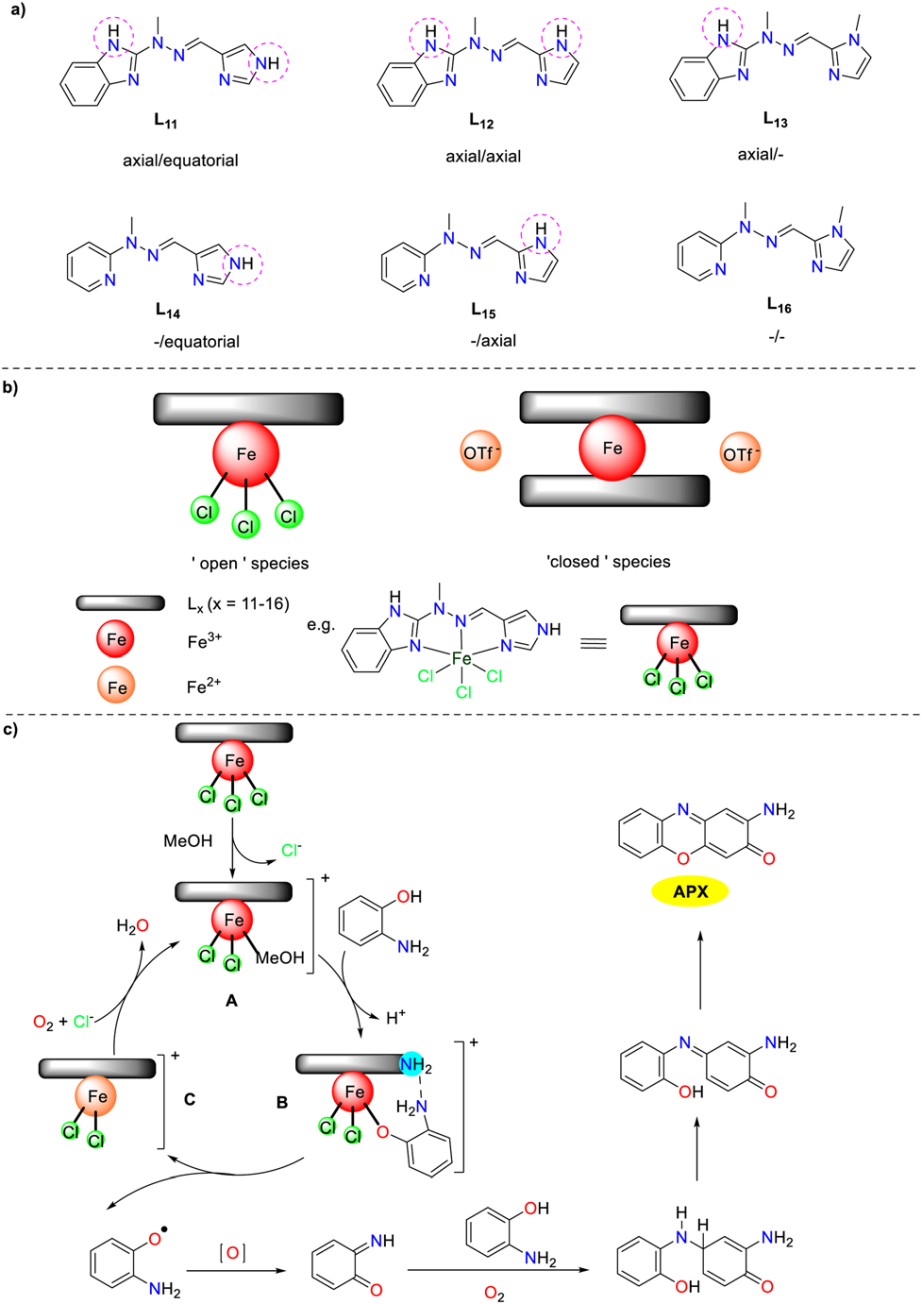
Ligands 11–16 and the proposed mechanism (ref. 38).

In 2019, a library of six tridentate N-heterocyclic ligands L_11_–L_16_ were synthesized by the Gorczyński group, differing primarily in the number and arrangement of hydrogen bond donors (Scheme 13).^38^ Coordination with iron(ii)/(iii) centers resulted in the formation of two types of complexes: 1:1 ‘open’ complexes with iron(iii) chloride and 1:2 ‘closed’ system with iron(ii) trifluoromethane sulfonate. These iron complexes were investigated as analogs of artificial phenoxazinone synthase and DNA-binding agents, which was the first study exploring the impact of hydrogen bonding on the multifunctional behavior of Schiff base iron agents in bioassays. The results suggest that the catalytic activity of each complex is determined by the interplay between its ‘primary’ structural features (open *vs.* closed) and ‘secondary’ structural features (the number and disposition of hydrogen bonds). The *K*_cat_ values of ‘open’ complexes were 185.25, 127.30, 172.30, 134.05, 103.34, and 150.49 h^−1^, while those of ‘closed’ complexes were 199.40, 97.68, 183.80, 42.76, 65.14, and 106.53 h^−1^. The ‘open’ complexes prone to ligand exchange initially undergo an exchange between chloride and methanol. This configuration allows for the interaction of OAP with the metal center in its deprotonated form, leading to the displacement of methanol and additional interaction through hydrogen bonding (N–H⋯N). The subsequent oxidation is believed to be the rate-determining step, explaining the observed time lag in the initial reaction. In addition, the desired APX scaffold is obtained through a series of redox transformations of a quinone imine intermediate with a second molecule of OAP.

**Scheme 14:**
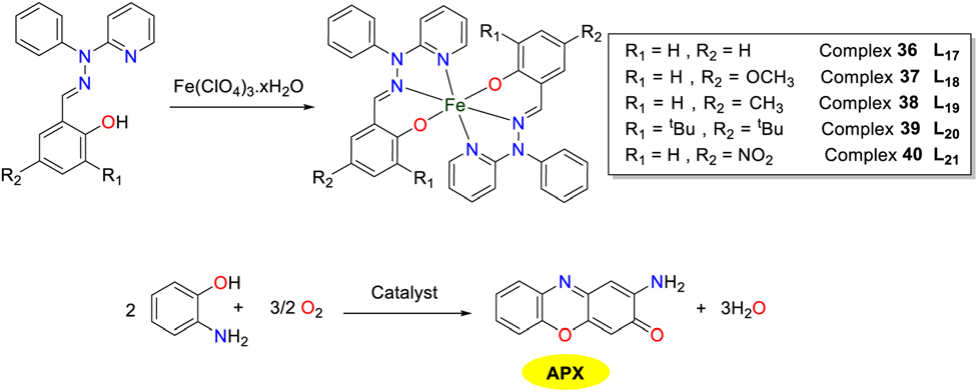
Synthesis of complexes 36–40 and the oxidation reaction of OAP to APX (ref. 39).

In 2021, the Ghosh group synthesized a series of tridentate ligands with non-innocent phenolate functions, which could coordinate *via* Npy and Nim donors upon deprotonation and further bound to an iron(iii) center for the formation of novel iron complexes 36–40 (Scheme 14).^39^ All of the iron complexes proved effective in catalyzing the oxidation of OAP under ambient conditions. The turnover frequencies (TOF) of complexes 36–40 were 16.89, 17.03, 16.92, 14.55, and 13.83 h^−1^, respectively. Notably, iron complexes 37 and 38 containing electron-donating groups (OCH_3_ and CH_3_) displayed higher oxidation activity for OAP. In contrast, despite the strong electron-donating property of the *tert*-butyl substituent in iron complex 39, its oxidation activity was lower than that of complexes 37 and 38. This decreased activity might be attributed to the steric hindrance caused by the bulky *tert*-butyl groups at the *ortho*- and *para*-positions, which might hinder substrate interaction. Additionally, the complex 40 with electron-withdrawing NO_2_ group was less efficient in catalyzing oxidation than complexes with and without substituents.

**Scheme 15:**
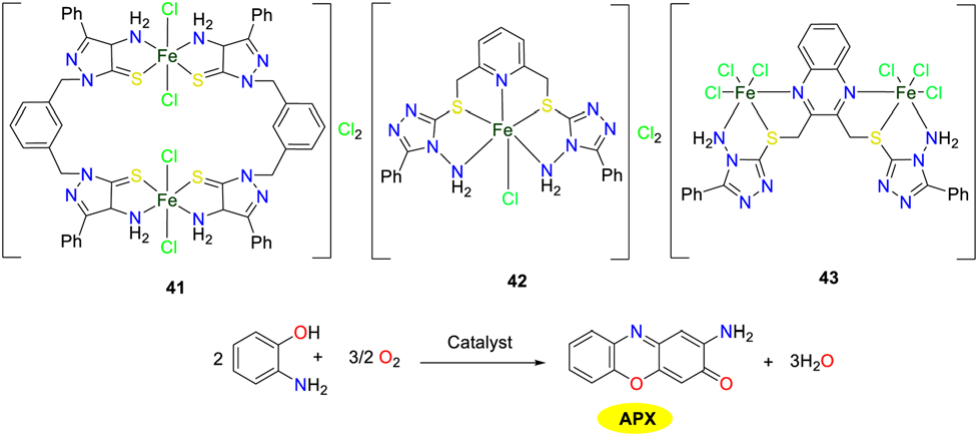
Preparation of complexes 41–43 and the oxidation reaction of OAP to APX (ref. 40).

In 2023, the synthesis and characterization of triazole-based iron(iii) complexes 41–43 were carried out by the Ramadan group (Scheme 15).^40^ The catalytic efficiency of the complexes was evaluated through the ratio *K*_cat_/*K*_M_ (*K*_cat_/*K*_M_ values of complexes 41–43 were 23.10, 14.14, and 41.95 × 10^3^ M^−1^ s^−1^), which indicated that the potencies were ranked as follows: complex 43 > complex 41 > complex 42. These disparities in catalytic performance might be attributed to the structural attributes that determine enzyme mimicry. For metal complexes mimicking oxidase enzymes, the presence of free or exchangeable *cis*-coordination sites on the metal ion is crucial for enabling the proximity binding of the substrate and oxygen. Metal complexes with labile anionic ligands such as Cl^−^ or low-coordination-number metal chelates are promising. Structural analysis of current iron(iii) complexes shows that complex 43 has these properties at both Fe(1) and Fe(2) nuclei, binuclear complex 41 only has them at Fe(1), while mononuclear complex 42 lacks the structural features.

### 2.3 Copper complex-catalyzed reactions

**Scheme 16:**
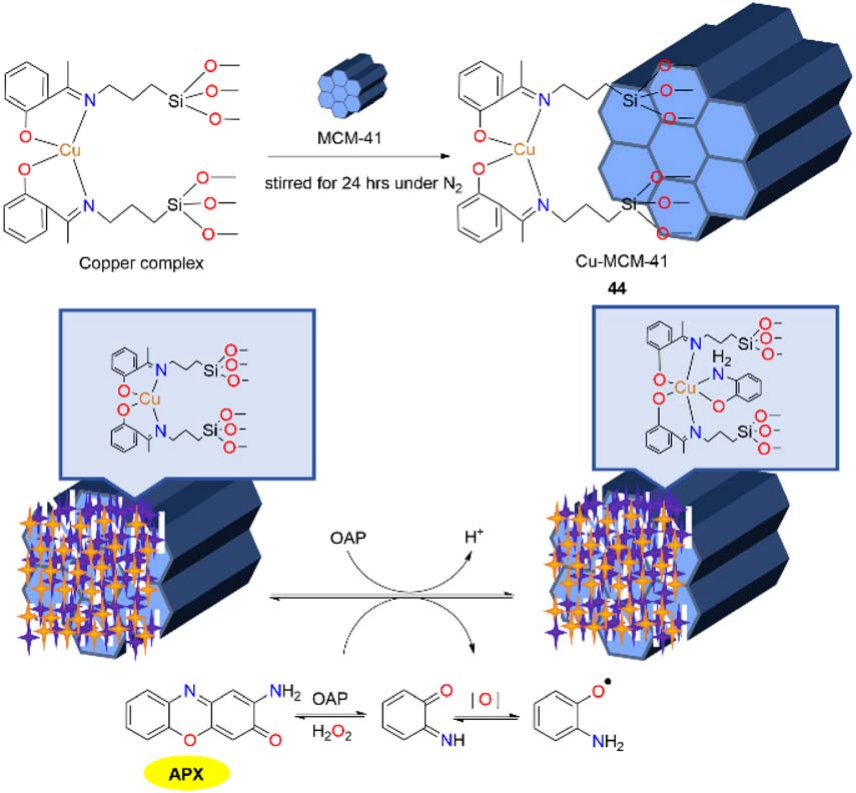
Synthesis of Cu-MCM-41 and the proposed pathway (ref. 41).

Given the relatively scarce exploration of heterogeneous catalysts within biomimetic processes, developing a silica-based material has opened up innovative prospects, particularly for the investigation of the kinetics of redox reactions. Inspired by the catalytic activities of enzymes, the Rangappan group has pioneeringly synthesized a mesoporous MCM-41-supported copper Schiff base complex 44, which mimicked the activity of phenoxazinone synthase in 2018 (Scheme 16).^41^ The copper complex with no metal leaching was shown to possess superior stability and activity compared to its homogeneous counterpart. Furthermore, the stability of the catalyst was confirmed through regeneration studies conducted over four consecutive cycles (the APX yield of each 3 h reaction was maintained at 40–42%). Given the significant application of actinomycin D in antibiotic synthesis and combinatorial therapies, the catalyst is anticipated to hold substantial potential in the pharmaceutical industry. Additionally, the catalyst with a similar structure to natural pigments found in insects and plants suggests that it may have promising application in bioinspired dye synthesis.

**Scheme 17:**
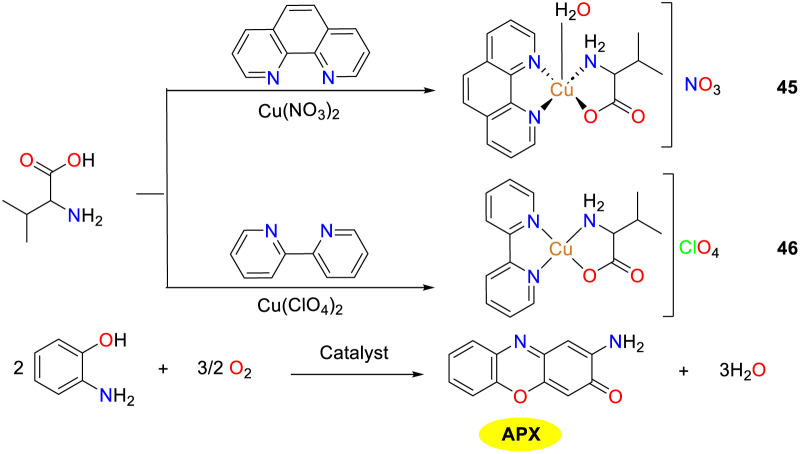
Formation of complexes 45–46 and the oxidation reaction of OAP to APX (ref. 42).

In 2019, to obtain copper complexes featuring [CuN_3_O] core, complexes 45 and 46 were synthesized and structurally characterized by Shaban and co-workers (Scheme 17).^42^ Copper complex 45 displays a distorted square-pyramidal geometry, while copper complex 46 adopts a distorted square-planar coordination geometry. The structures of complexes 45 and 46 are stabilized by a supramolecular network formed through intermolecular and intramolecular hydrogen bonding interactions. Both complexes were evaluated as functional models for phenoxazinone synthase, and copper complex 45 exhibited a slightly higher activity than copper complex 46 (the *K*_cat_ values were 2.1 × 10^3^ and 4.3 × 10^3^ h^−1^, respectively), which might be attributed to the following reasons. The coordinately saturated nature of complex 45 demands energy for ligand dissociation, a crucial factor influencing its catalytic properties. In contrast, copper complex 46, with its distorted square-planar geometry, has a vacant coordination site for substrate binding, eliminating the requirement for ligand dissociation energy. Furthermore, the distorted square-planar structure of complex 46 resembles the active-site Cu center of catechol oxidase, which are neither tetrahedral nor strictly square planar, thereby minimizing geometric changes during the Cu(ii) to Cu(i) transformation and enhancing the stability and efficiency of the active site.

**Scheme 18:**
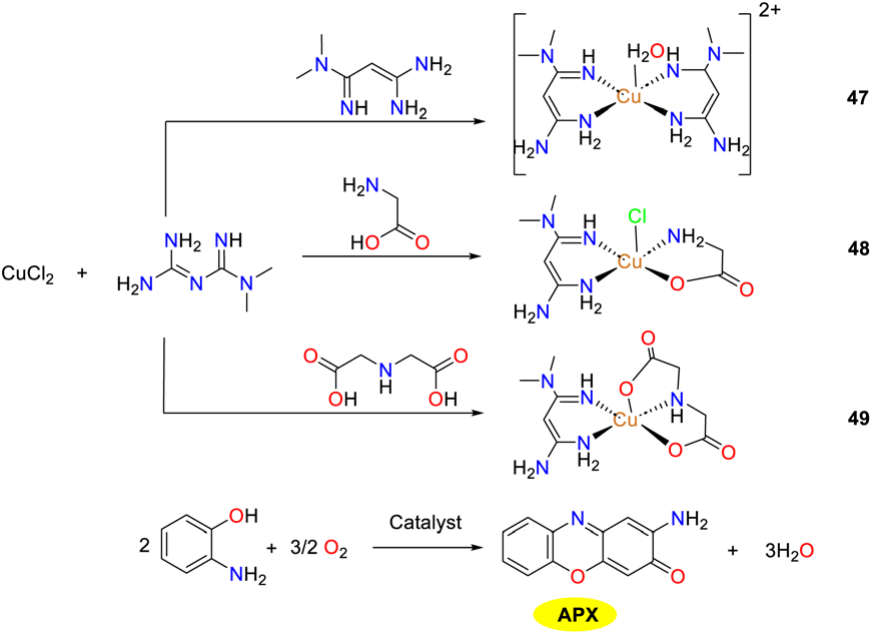
Synthesis of complexes 47–49 and the oxidation reaction of OAP to APX (ref. 43).

In 2020, the Ramadan group synthesized and characterized three Cu(ii) complexes 47–49 derived from metformin, which exhibited a promising potential to emulate the functional site of phenoxazinone synthase moiety, thus offering valuable insights into the development of novel efficacious copper oxidase mimics (Scheme 18).^43^ All copper complexes exhibit a pentacoordinate architecture, with the substrate engaging in metal-centered ligand exchange processes. H_2_O and chloride Cl^−^ are identified as potential ligand departure entities in complexes 47 and 48, respectively. Notably, the weaker coordination capacity of water facilitates its easier displacement than Cl^−^, leading to a stronger substrate binding affinity of copper complex 47 compared to copper complex 48. For copper complex 49, the absence of the leaving group renders a penta-coordinate ligand system, suggesting a possible hexa-coordination with an octahedral geometry. However, the instability of the resulting hexacoordinated catalyst–substrate intermediate significantly hampers its formation due to the Jahn–Teller effect. Consequently, copper complex 49 displays a lower catalytic efficiency than that of copper complexes 47 and 48. The *K*_cat_/*K*_M_ values of complexes 47–49 were 1.22 × 10^6^, 5.66 × 10^6^, and 1.35 × 10^6^ h^−1^ M^−1^, respectively. Although the binding affinity of the three complexes to the investigated substrate exhibits the order of 47 > 48 > 49, the catalytic efficiency follows the sequence of 48 > 47 > 49. This discrepancy can be attributed to structural factors. Specifically, the geometric irregularity of complex 48 surpasses that of complex 47.

**Scheme 19:**
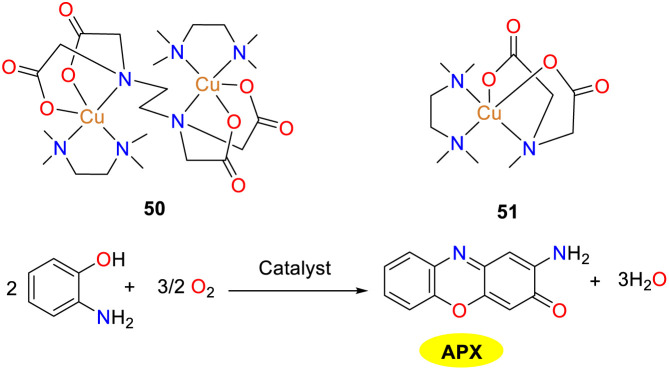
Structures of complexes 50–51 and the oxidation reaction of OAP to APX (ref. 44).

Intending to attain a more profound comprehension of diverse chemical and biological oxidation of OAP, Ramadan and co-workers synthesized two ternary copper(ii) complexes in 2020 (Scheme 19).^44^ These copper complexes employed a mixed ligand system comprising 1,1′,4,4′-tetramethylethylenediamine in conjunction with either *N*-methyliminodiacetic acid or ethylenediaminetetraacetic acid. Both copper complexes were proposed to adopt a square pyramidal geometry in their homonuclear and mononuclear forms. Complexes 50 and 51 manifested the capacity to catalyze the oxidation of OAP to generate phenoxazinone, demonstrating phenoxazinone mimetic activity. Despite their comparable redox potential values, copper complex 50 displayed a conspicuously higher catalytic activity than that of copper complex 51. The *K*_cat_/*K*_M_ values of complexes 50–51 were 7.5 × 10^4^ and 1.724 × 10^4^, respectively. This disparity in catalytic performance is ascribed to the difference in the coordination affinity of the substrate. The *K*_M_ value of complex 50 was 2 × 10^−3^ M, while that of complex 51 was 2.9 × 10^−3^ M.

**Scheme 20:**
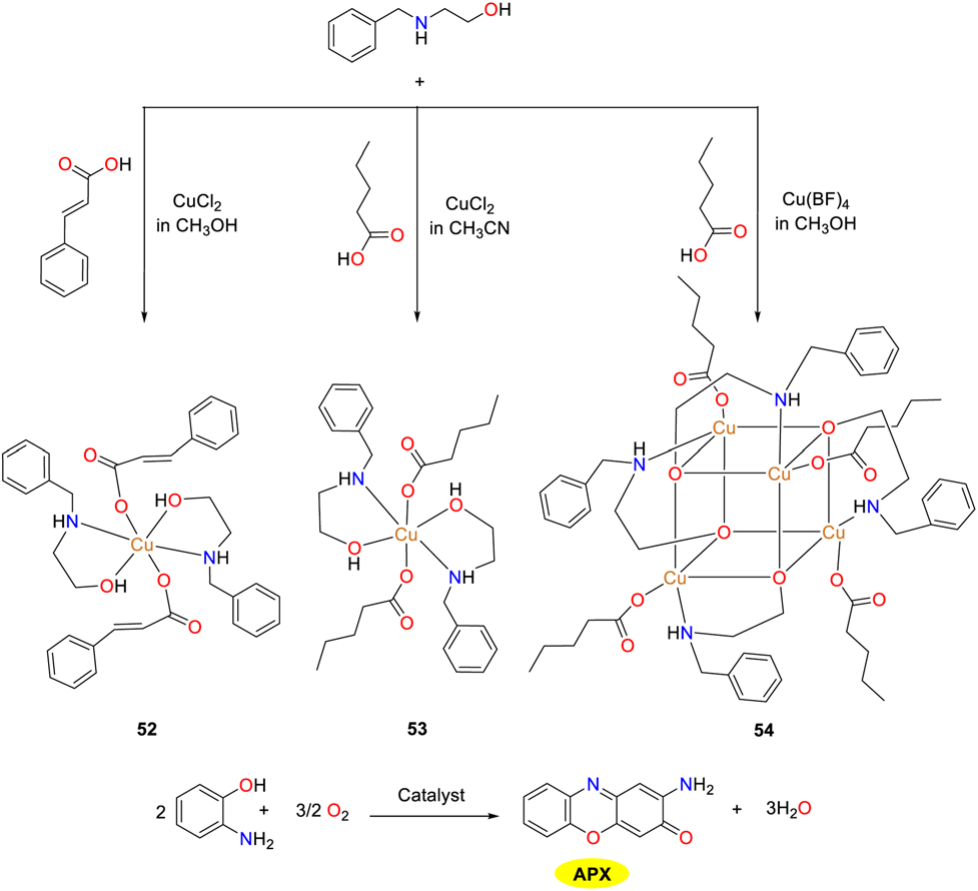
Synthesis of complexes 50–52 and the oxidation reaction of OAP to APX (ref. 45).

Three novel compounds 52–54 were synthesized by the Nesterova group *via* a self-assembly reaction of 2-benzylaminoethanol with cinnamic acid or valeric acid catalyzed by copper chloride and copper tetrafluoroborate (Scheme 20).^45^ These complexes represent the first mixed-ligand system to simultaneously incorporate 2-benzylaminoethanol and cinnamic acid (valeric acid) in their basic forms with metal ions. Notably, all compounds exhibited phenoxazinone synthase-like activity in methanol, with copper complex 52 demonstrating the optimal catalytic efficiency, which indicated that the tetranuclear species in copper complex 52 significantly outperforms the tri- and dinuclear species in terms of reaction rate. The *V*_max_ values were 4.0 × 10^−7^, 2.5 × 10^−7^ and 2.1 × 10^−7^ M s^−1^, respectively, supported by the quantitative yield of the product after 24 h. The observed rate was among the highest record for this type of reaction. Mechanistic and isotopic ^18^O-labelling studies revealed that OAP is likely oxidized by active Cu(ii). These findings are expected to advance the understanding of biomimetic oxidation mechanism in phenoxazinone synthase and to stimulate further research in this area.

**Scheme 21:**
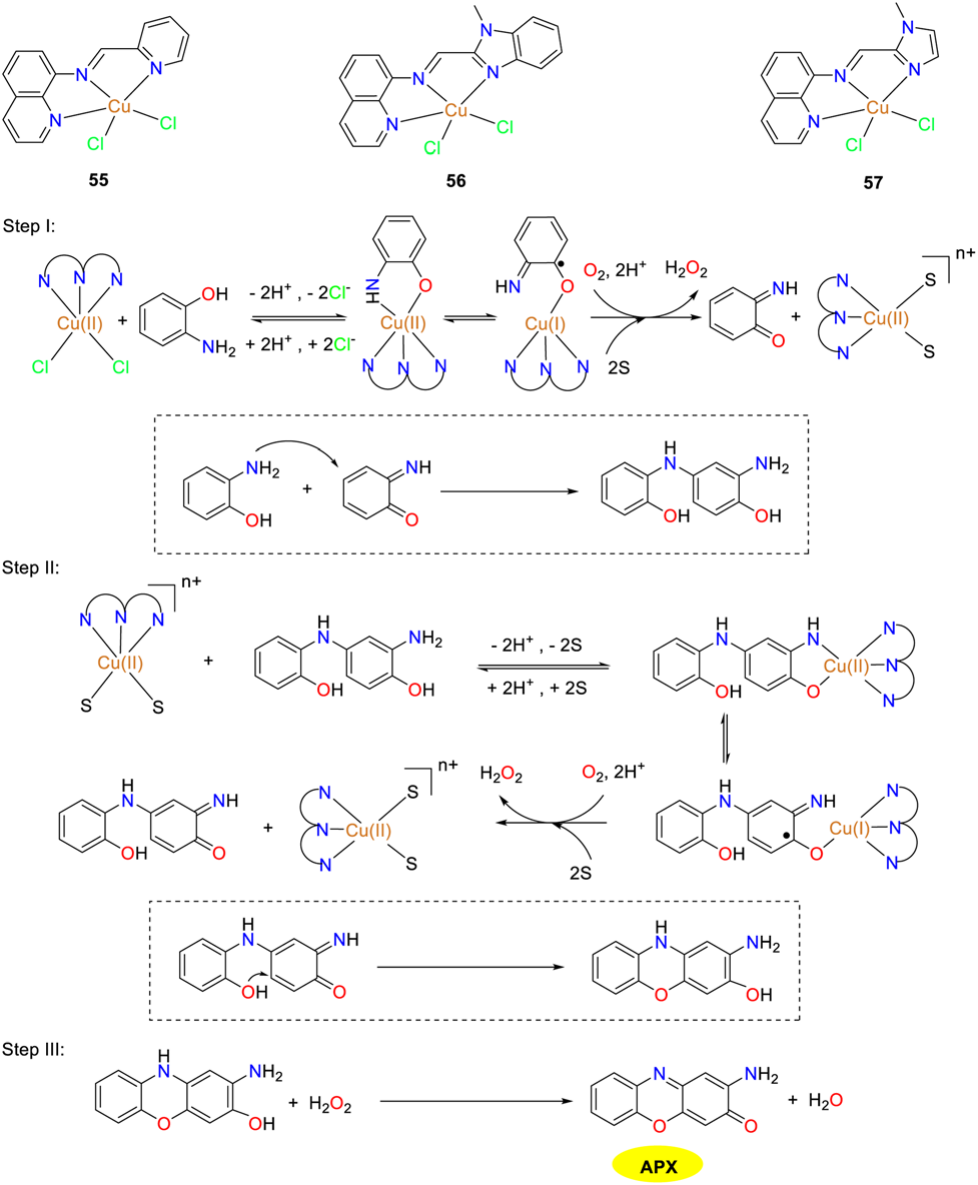
Structures of complexes 55–57 and the proposed mechanism; letter S represents solvent molecules in this scheme (ref. 46).

In the same year, the Mandal group documented the synthesis and detailed characterization of three mononuclear copper(ii) complexes 55–57, which were stabilized by Schiff base N_3_ donor ligands featuring diverse donor moieties (Scheme 21).^46^ The authors investigated the phenoxazinone synthase-mimetic activities of three copper complexes by the aerial oxidation of *o*-aminophenol to 2-amino-phenoxazine-3-one in a methanol–water solvent system at pH 8.6. Kinetic studies demonstrated that the catalytic efficiency is of the order of complex 57 ≫ 56 > 55. The *K*_cat_/*K*_M_ values of complexes 55–57 were 12, 16, and 44 M^−1^ s^−1^, respectively. Based on the ESI-MS data, kinetic analysis, and reaction stoichiometry, a plausible reaction pathway is outlined, in which the overall six-electron transfer occurs sequentially involving three consecutive two-electron transfer steps. Consequently, the findings presented in this study hold significant implication for advancing the understanding of phenoxazinone synthase-like activity.

**Scheme 22:**
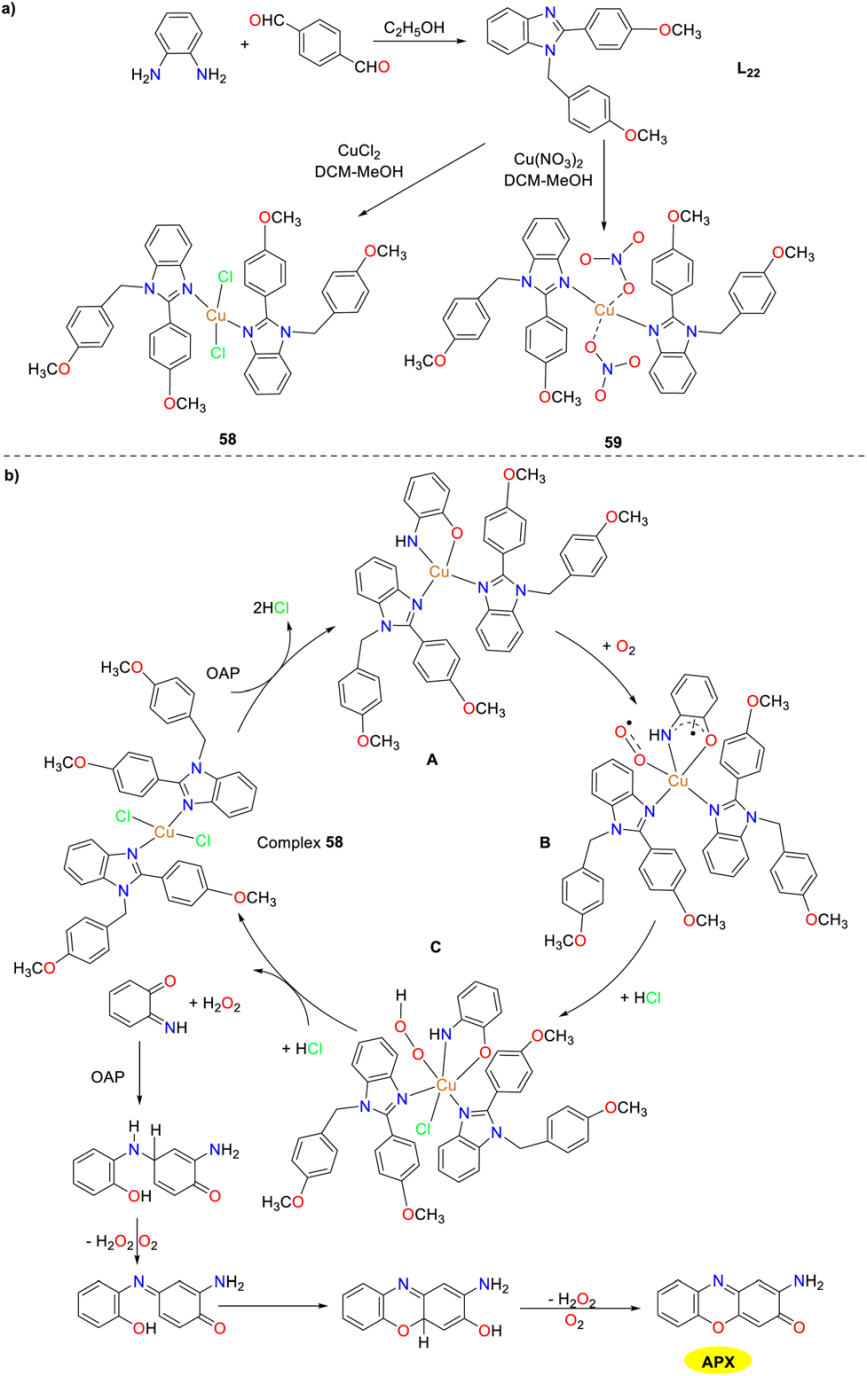
Synthesis of complexes 58–59 and the proposed mechanism (ref. 47).

In 2021, the Biswas group reported a newly designed substituted benzimidazole ligand L_22_ and two structurally similar copper(ii) complexes 58 and 59, while studying the effect of auxiliary factors (chloride and nitrate) on the activity of phenoxazinone synthase in biomimetic simulations (Scheme 22).^47^ Both copper complexes adopt a perfect planar square geometry and exist in a *trans* configuration, which is the driving force for the formation of enzyme–substrate adducts, giving the complexes excellent OAP catalytic oxidation performance. The activity of copper complex 59 is twice that of copper complex 58. The *K*_cat_/*K*_M_ values were determined as 8.78 × 10^6^ and 1.50 × 10^7^ for 58 and 59. In Cu(ii) complex 59, the coordinated nitrate around the Cu(ii) center causes high steric hindrance and greater lability. As a result, the coordinated nitrate is likely to detach when OAP approaches the Cu(ii) center. In contrast, in complex 58, the coordinated chloride in the square-plane of the Cu(ii) center leads to a more stable and inert structure. Moreover, when complex 59 reacts with OAP, the coordinated nitrates are displaced into the solution. The increased lability or coordinative unsaturation at the Cu(ii) center of complex 59 contributes to its higher catalytic performance than complex 58.

**Scheme 23:**
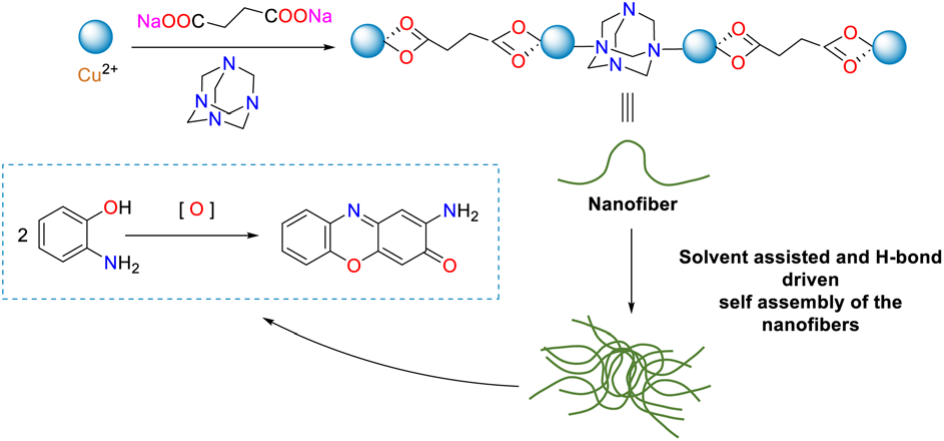
Schematic of the nanofibrous metallogel formation and its phenoxazinone synthetase activity (ref. 48).

In the same year, Sarkar and co-workers successfully synthesized the nanodimensional metallogel through the reaction of disodium succinate and hexamethylenetetramine in the presence of CuCl_2_ in an aqueous medium (Scheme 23).^48^ The resulting gel displayed a morphological character by interweaving the nanofiber and the extensive fibrous network. The stability of the gel phase was found to be affected by various chemical stimuli. Importantly, the Cu(ii)-containing gel exhibited exceptional catalytic activity as a heterogeneous catalyst in transforming *o*-aminophenol into phenoxazinone. This PHS-like catalytic behavior of the nanoscale metallogel is unprecedented and suggests its potential for further exploration in related catalytic contexts. Additionally, the catalytic mechanism was investigated, and the recyclability of the catalyst was demonstrated.

**Scheme 24:**
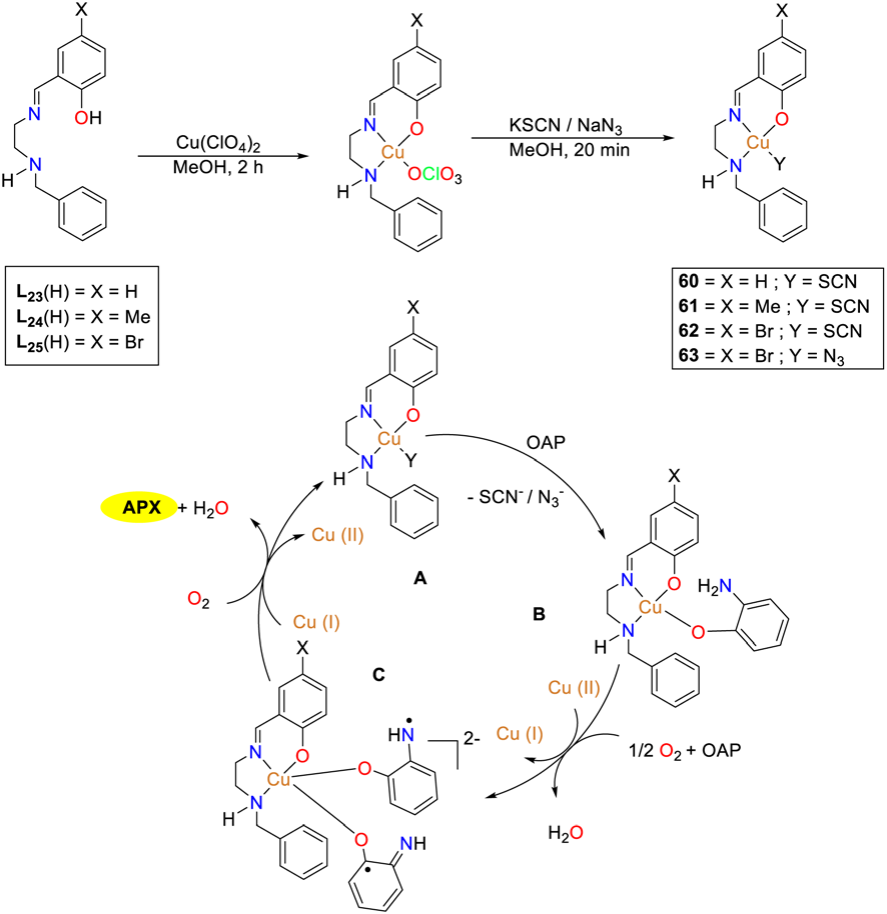
Synthesis of complexes 60–63 and the proposed mechanism (ref. 49).

In 2022, the Sankaralingam group synthesized three tridentate Schiff base ligands L_21_–L_23_ along with their copper thiocyanate complexes and a copper azide complex, and investigated the catalytic activity of mononuclear copper(ii) complexes (Scheme 24).^49^ The synthesized copper complexes all demonstrated phenoxazinone synthase mimetic activity, among which, copper complex 62 exhibited the highest conversion of OAP to APX. The *K*_cat_ values of complexes 60–63 were 7.8 × 10^5^, 2.4 × 10^5^, 6.2 × 10^6^, and 3.0 × 10^6^ h^−1^, respectively. The different catalytic activities between complexes 60 and 62 are attributed to the substituents at the *para* position of the ligand and auxiliary factors. Since the sizes of Br and CH_3_ are comparable, the steric effect on the yield of APX could be ruled out. Instead, it appears that the electron-withdrawing nature of Br enhances the catalytic efficiency of copper complex 62. Thus, it can be concluded that the electronic inductive effect within the ligand significantly influences the catalytic efficacy of these Cu(ii) complexes. The minor variation in the reaction rate between complexes 62 and 63 indicates that the –SCN group has a superior leaving ability in the catalytic process.

**Scheme 25:**
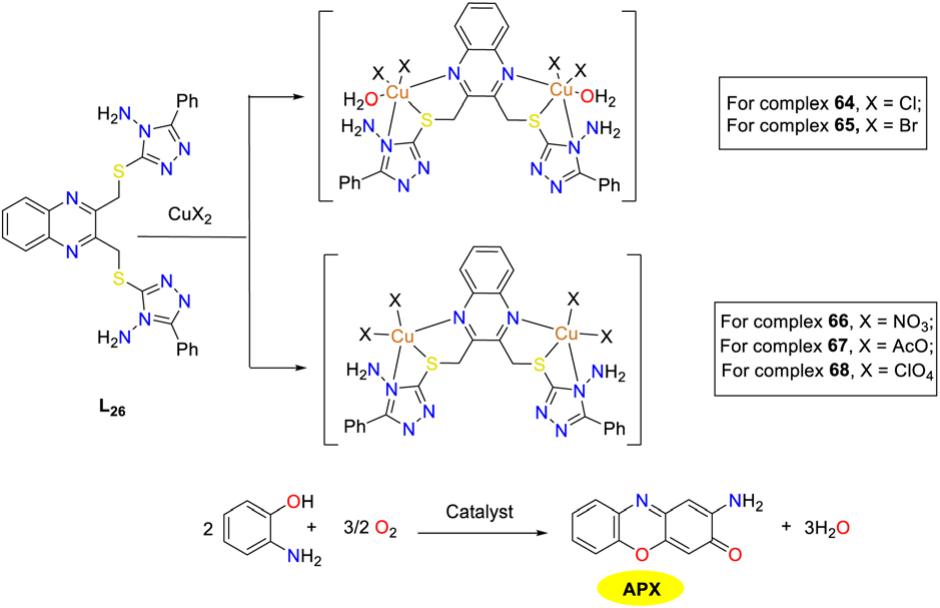
Synthesis of complexes 64–68 and the oxidation reaction of OAP to APX (ref. 50).

In the same year, the Ramadan group synthesized several hexadentate ligands featuring quinoxaline backbone and their Cu(ii) complexes with various anionic salts (Scheme 25).^50^ Monatomic halogen ligands predominantly favored an octahedral geometry, whereas larger polyatomic ligands led to a trigonal bipyramidal geometry. The oxidase-mimetic catalytic activity such as phenoxazine synthase activity was modulated by the anionic ligands present in the Cu(ii) oxidase mimics. Among the five-coordinate copper complexes, the nitrate complex demonstrated the highest activity. The *K*_cat_/*K*_M_ values of complexes 64–68 were 24.44 × 10^4^, 40.43 × 10^4^, 85.50 × 10^4^, 51.01 × 10^4^, and 68.51 × 10^4^ M^−1^ min^−1^, respectively. This study underscores the impact of both organic and anionic ligands on the redox behavior of Cu(ii) complexes, providing valuable insights into the design of metal complexes with customized structural and catalytic properties.

**Scheme 26:**
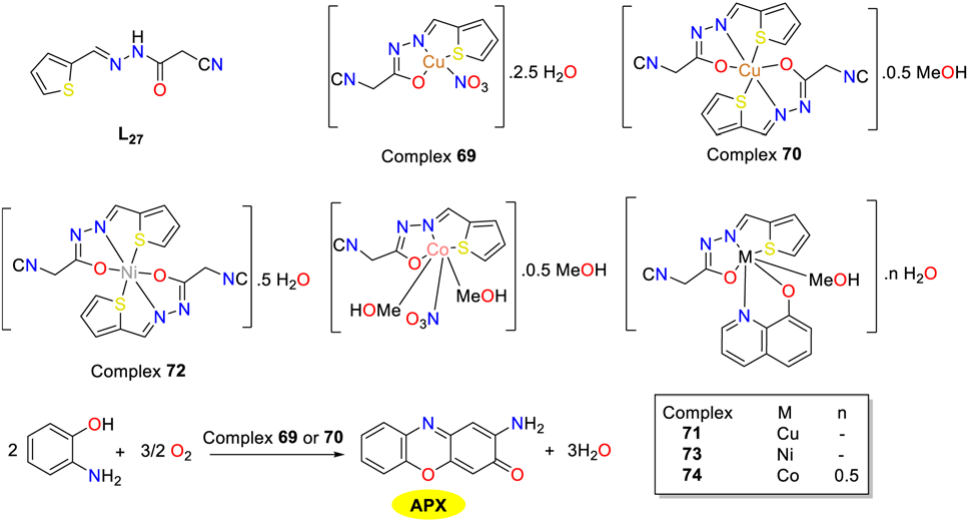
Synthesis of complexes 69–75 and the formation of APX (ref. 51).

In 2022, El-ghamry and co-workers reported the synthesis of a novel tridentate hydrazone ligand (L_27_) and its metal complexes including Cu(ii) complex 69 and 70, Ni(ii) complex 72, and Co(ii) complex 74 (Scheme 26).^51^ The protocol was further extended by devising mixed-ligand complexes employing L_27_ and 8-hydroxyquinoline as the substrates (complexes 71, 73, and 75). The investigation of the bioactivity revealed that the parent ligand L_27_ exhibited augmented efficacy when complexed with metallic ions. Specifically, the copper(ii) complex 69 demonstrated potent antibacterial properties against *Bacillus subtilis* and exerted significant anticancer effects against HepG-2 cells. Moreover, the copper complexes 69 and 70 displayed phenoxazinone synthase enzyme-like activities, with copper complex 69, demonstrating superior performance. The *K*_cat_ values of complexes 69–70 were 1.77 × 10^2^ and 15.03 h^−1^, respectively. This heightened activity of copper complex 69 might be attributed to the presence of a nitrate moiety, which acted as an efficient leaving group, thereby promoting substitution by the 2-aminophenol during the catalytic cycle.

**Scheme 27:**
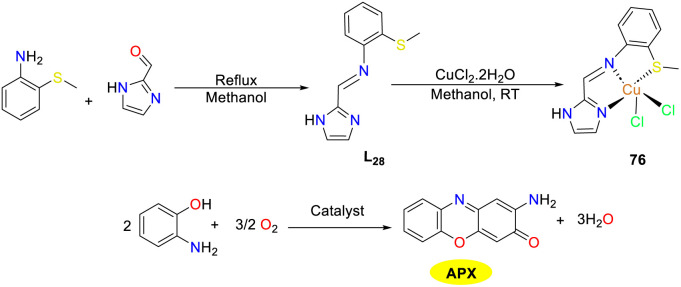
Preparation of complex 76 and the oxidation reaction of OAP (ref. 52).

In 2022, the Das team reported a mononuclear bis-chloro copper(ii) complex 76 employing a novel electron-deficient tridentate thiomethyl-substituted imidazole-based Schiff base as the ligand, which exhibited a distorted square-pyramidal geometry with two chlorides at axial and equatorial positions (Scheme 27).^52^ The catalytic activity of this redox-active copper(ii) complex 76 was investigated for the aerobic oxidation of OAP to APX, mimicking the phenoxazinone synthase activity. The copper(ii) complex demonstrated excellent catalytic efficiency with a *K*_cat_/*K*_M_ value of 13.15 × 10^6^. Additionally, the oxidation process could be scaled up to gram quantities, achieving approximately 86% yield of APX. Of note, copper complex 76 also exhibited significant antibacterial activities against *E. coli*, *Staphylococcus aureus*, and *K. pneumoniae*, as well as anticancer activities against the human colorectal adenocarcinoma HT-29 cell line.

**Scheme 28:**
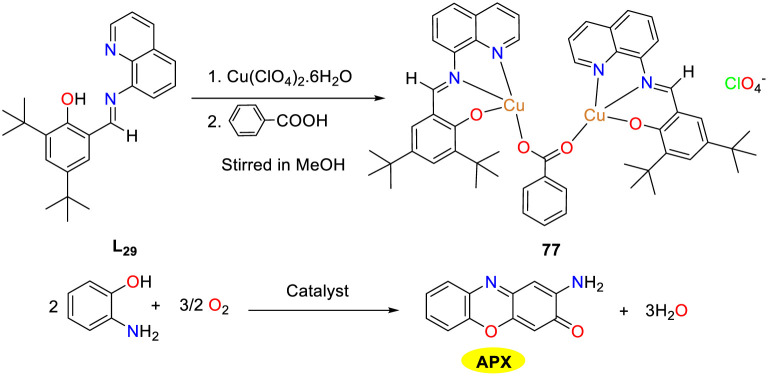
Preparation of complex 77 and the oxidation reaction of OAP to APX (ref. 53).

In 2023, the Mondal group investigated the extensive and diverse effects of copper ions on various biological processes and synthesized the homodimeric copper complex 77 utilizing a benzoate-bridged salicylaldehyde imine Schiff base NNO as the clamp ligand through a one-pot strategy (Scheme 28).^53^ The copper complex 77 was thoroughly characterized, and its biocatalytic activity was assessed during the air oxidation of OAP in an acetonitrile solution by spectrophotometry. The results indicated a gradual increase in the intensity of a peak at 436 nm in the time-dependent UV-VIS spectral scan, suggesting that OAP was oxidized into APX under the catalysis of the Cu(ii) complex. The calculated *K*_M_ value was 1.947 × 10^−3^ M, and the *K*_cat_ value was 5.244 h^−1^. In contrast, the mixture of ligand and Cu(ii)perchlorate showed no catalytic activity, which confirmed that the Cu(ii) complex was essential for the catalytic cycle.

**Scheme 29:**
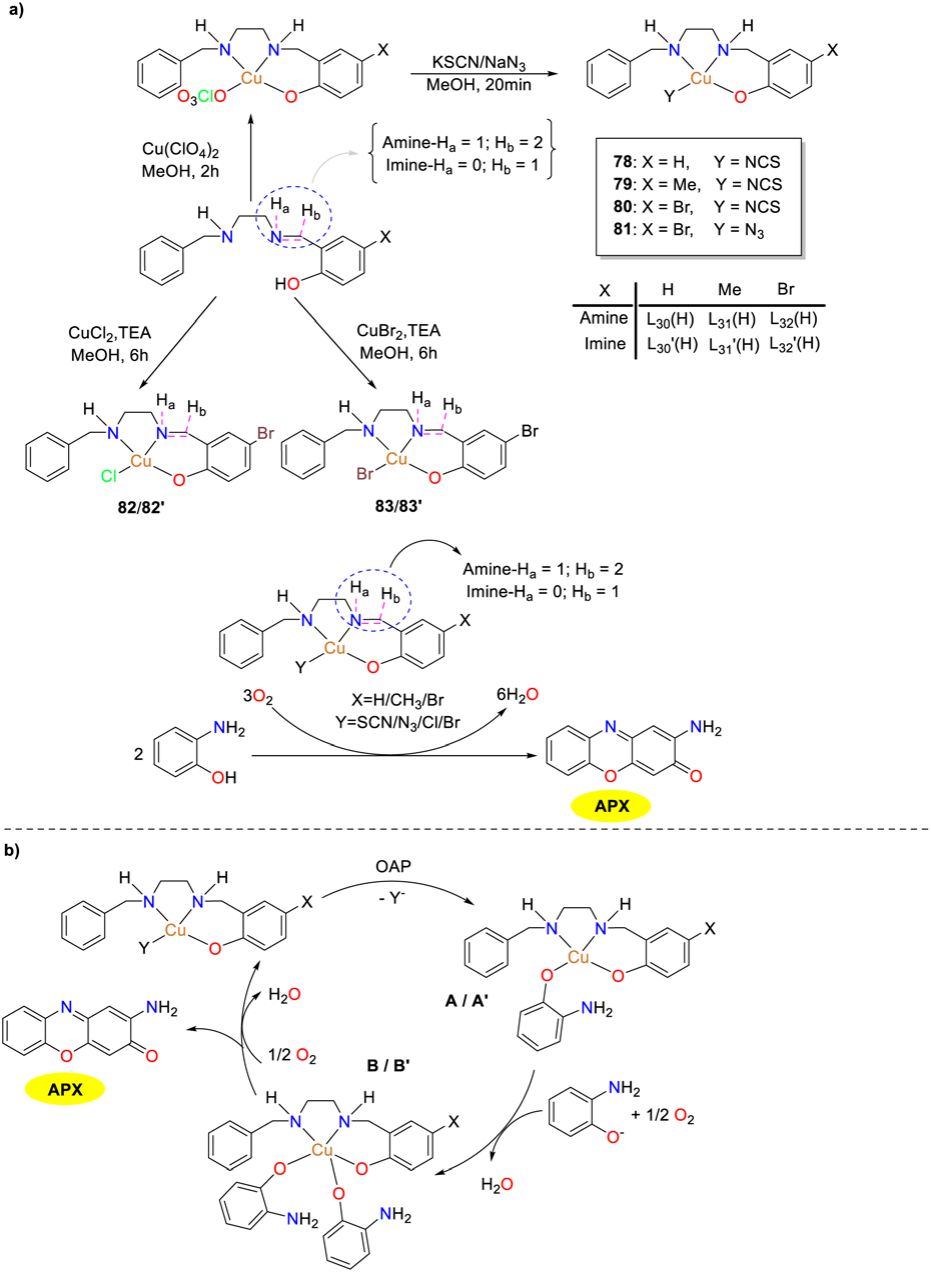
Synthesis of complexes 78–83 and the mechanistic steps of the oxidation reaction (ref. 54).

In the same year, the Sankaralingam group presented the synthesis and characterization of copper(ii) complexes 78–83 based on tridentate NNO donor ligands encompassing both amine and imine types (Scheme 29).^54^ These copper complexes exhibited remarkable reactivity in the oxidation of OAP, resembling the activity of phenoxazinone synthase enzyme models. The reactivity was governed by the substituents on the ligands and the presence of auxiliary ligands in the metal complexes. The kinetic measurement revealed that the imine-based Cu(ii) complexes (*K*_cat_, 2.4 × 10^5^ to 6.2 × 10^6^ h^−1^) are better than amine-based (*K*_cat_, 6.3 × 10^4^ to 3.9 × 10^5^ h^−1^) complexes. Mass spectrometry was employed to capture the complex–substrate mono-adducts A and A′, which assisted in the identification of radical-centered Cu(i) intermediate (B/B′). This intermediate facilitated the formation of the phenoxazinone chromophore with the regeneration of the active catalyst. Thus, mononuclear copper(ii) complexes with tridentate Schiff base ligands featuring a benzylamine group were demonstrated to achieve high PHS activity effectively.

**Scheme 30:**
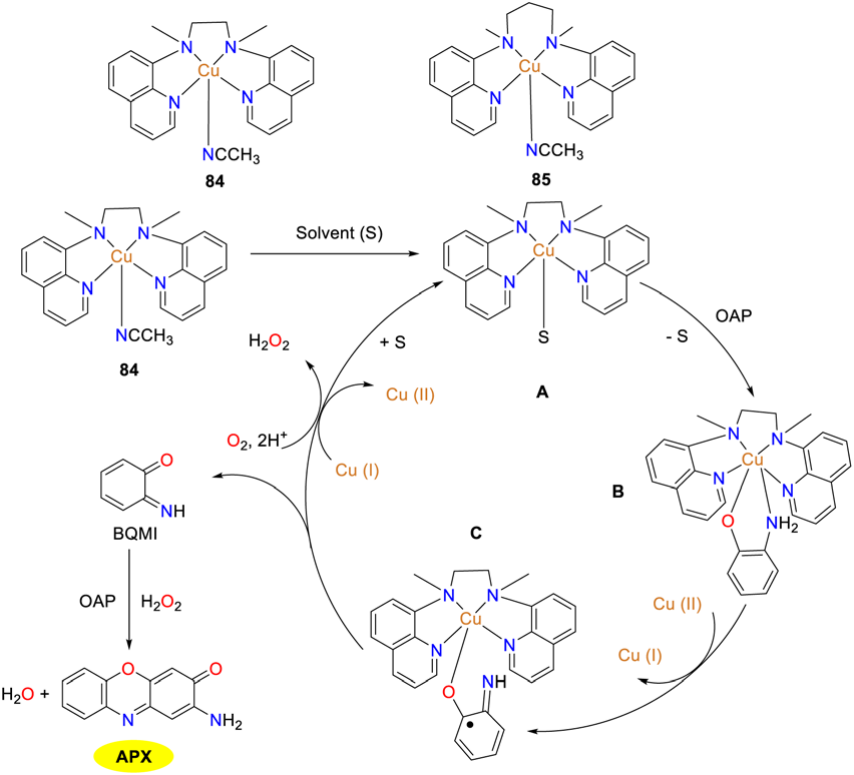
Structures of complexes 84–85 and the proposed mechanism (ref. 55).

In 2023, the mononuclear Cu(ii) complexes 84 and 85 supported by quinoline-based tetradentate non-heme ligands were synthesized and characterized by the Dhuri group to further understand the relationship between the structure and the reactivity (Scheme 30).^55^ Of note, Cu(ii) complexes 84 and 85 differ by a single carbon in their carbon chain backbones, resulting in two distinct geometries. Cu(ii) complexes 84 and 85 showed good reactivity to OAP, affording APX in 84% and 72% yields with *K*_cat_ values of 71.94 and 55.19 h^−1^, respectively. The mechanism for the generation of APX by the oxidation of OAP is illustrated below. The tetragonal cone geometry of copper complex 84 facilitates easier substrate binding to the copper center, allowing solvent molecules to be readily replaced by the substrate from vacancies. Conversely, the structure of copper complex 85 is between a planar square and a triangular bipyramid, which is more challenging for the substrate to bind.

**Scheme 31:**
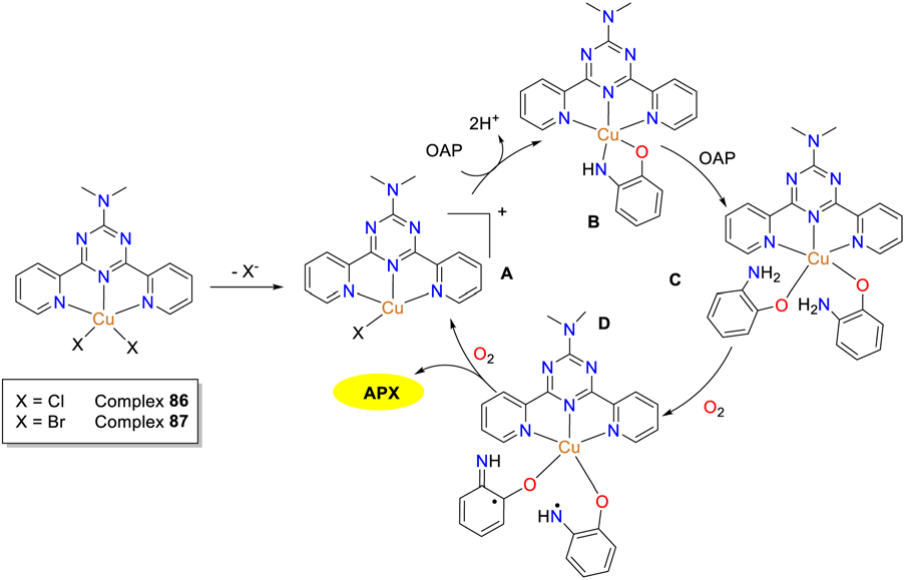
Probable mechanism of the oxidation reaction of OAP to phenoxazinone by complex 86 or 87 (ref. 56).

To get better insights into the structure–activity relationship, two penta-coordinated *cis*-dichloro and dibromo Cu(ii) complexes 86 and 87 were synthesized by the Maji group in 2024 (Scheme 31).^56^ The copper complexes revealed the distorted square pyramidal geometry around the central metal atom, where halogen atoms were found to be bonded to the Cu(ii) center in a *cis*-fashion. The apical metal–halogen bond was slightly more elongated than the corresponding equatorial bond, which was more susceptible to cleavage for adduct formation and ultimately facilitated their catalytic performance toward the oxidation of OAP in their respective methanolic solutions. The *K*_cat_ value of Cu(ii) complex 87 (*K*_cat_ = 156.7 h^−1^) is larger than that of complex 86 (*K*_cat_ = 124.5 h^−1^), which may be attributed to the greater structural instability and higher redox potential of complex 87. This present work provided valuable information regarding the effect of structural liability on the design and understanding of catalysts and their biomimicking activity, which furnished designing strategy for the generation of bioinspired catalyst.

**Scheme 32:**
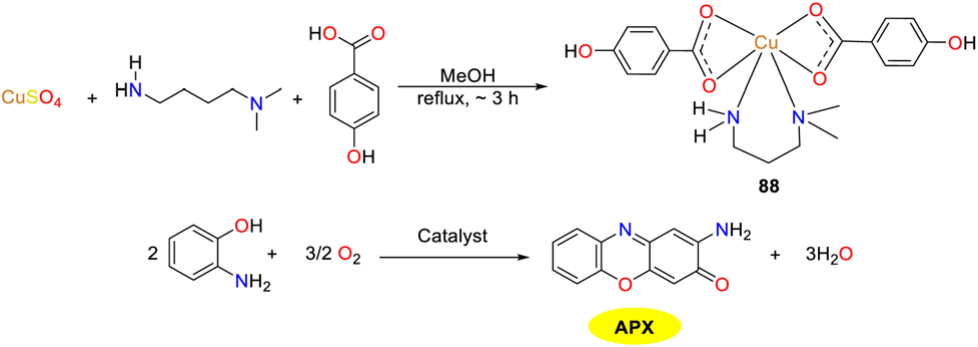
Synthesis of complex 88 and the oxidation reaction of OAP to APX (ref. 57).

In the same year, the Das group reported a newly synthesized Cu(ii) complex 88 incorporating *para*-hydroxybenzoic acid and propylamine ligands, and acquired a profound comprehension of the mechanism of metalloenzymes, which was conducive to develop biologically inspired catalysts with comparable efficacy (Scheme 32).^57^ The octahedra of the copper complex was arranged around copper ions, giving rise to geometric distortions due to extension along one axis and one equatorial direction. Hirshfeld surface analysis revealed that there were hydrogen bonds and C–H–π interactions between and within the molecules. EPR, CV, and UV analyses jointly supported that under thoroughly aerobic conditions, it demonstrated oxygen-dependent enzymatic radical reactivity towards *o*-aminophenol, resulting in the formation of phenoxazinone compound (*K*_cat_ = 0.260 × 10^5^ h^−1^).

**Scheme 33:**
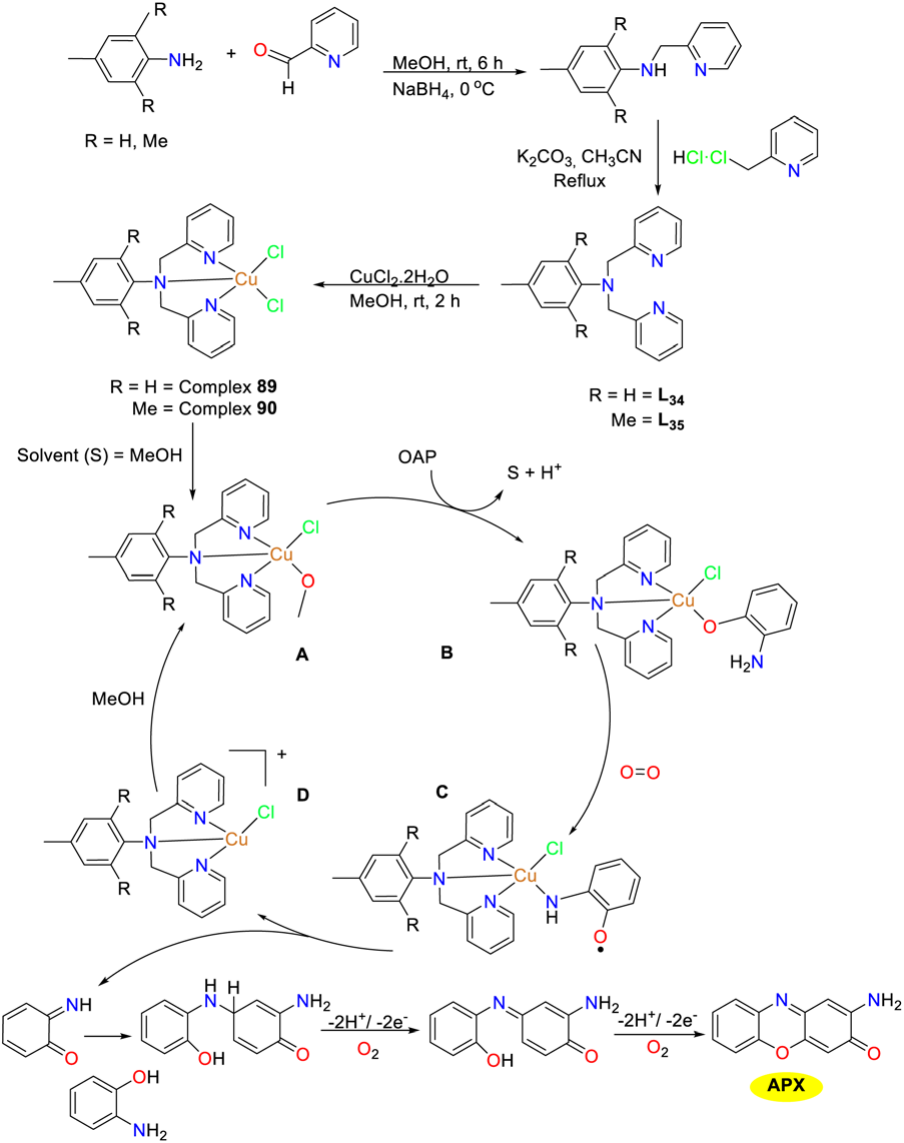
Synthesis of complexes 89–90 and the proposed mechanism (ref. 58).

In 2024, Maji and co-workers was inspired by the structure of natural catechol oxidase and synthesized two copper(ii) complexes 89 and 90 derived from dipyridine amine (DPA) with tridentate ligands employing *p*-toluidine, 2-pyridine carboxaldehyde, and 2-(chloromethyl)pyridine as raw materials through stepwise reactions (Scheme 33).^58^ The unstable nature of the chloride ion at the top of these tetragonal cone complex allows them to adhere to substrate easily. Of note, OAP was easily converted into phenoxazinone utilizing copper(ii) complex as the catalysts under aerobic conditions in a methanol solution, exhibiting excellent reaction activity. The complexes 89 and 90 had *K*_cat_ values of 525.55 h^−1^ and 255.32 h^−1^, respectively. The lower activity of 90 stems from structural tuning influencing ligand lability and substrate–metal binding. Crystal structure reveals a longer, weaker metal–halogen bond in 89, facilitating cleavage and complex–substrate adduct formation. This protocol provided a new platform for designing suitable biomimetic catalysts.

**Scheme 34:**
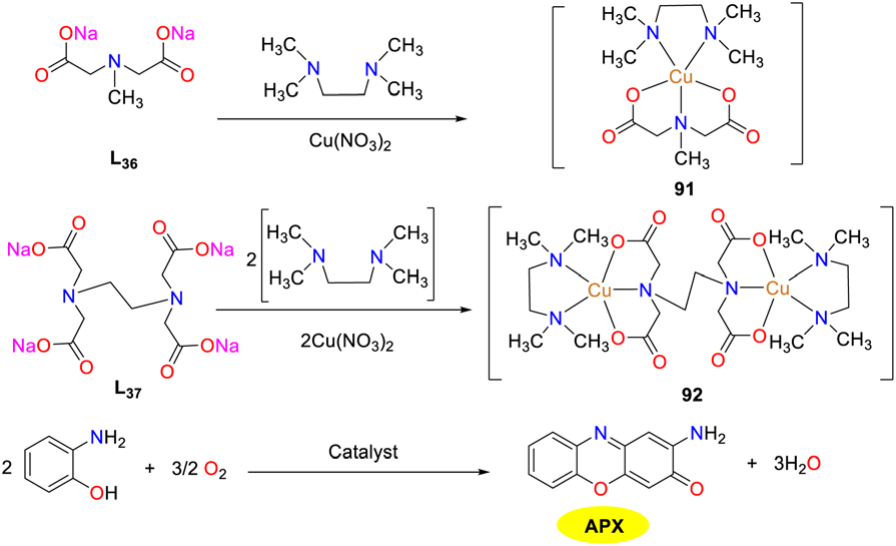
Synthesis of complexes 91–92 and the oxidation reaction of OAP to APX (ref. 59).

In the same year, the Fathy group accomplished the characterization and synthesis of dual ligand Cu(ii) complexes 91 and 92 through the incorporation of both N and O donors, emulating the behavior of copper proteins (Scheme 34).^59^ An observation particularly noteworthy was that binuclear complex 92 exhibited a superior phenoxazinone synthase functionality to its mononuclear counterpart 91. The *K*_cat_/*K*_M_ values of complexes 91 and 92 were 6.84 × 10^3^ and 266.05 × 10^3^ M^−1^ s^−1^, respectively, which echoed the structural similarity between natural binuclear catechol oxidases and poly copper phenoxazinone synthetases. Notably, the investigations revealed a nuanced understanding of their catalytic dynamics, emphasizing that no solitary factor governed the efficiency of these oxidase mimic catalysts. In addition, the catalytic performance of copper complexes was attributed to the affinity of phenolic substrates, the redox potential disparities between phenol and Cu(ii) complexes, and alongside the energetic landscape.

**Scheme 35:**
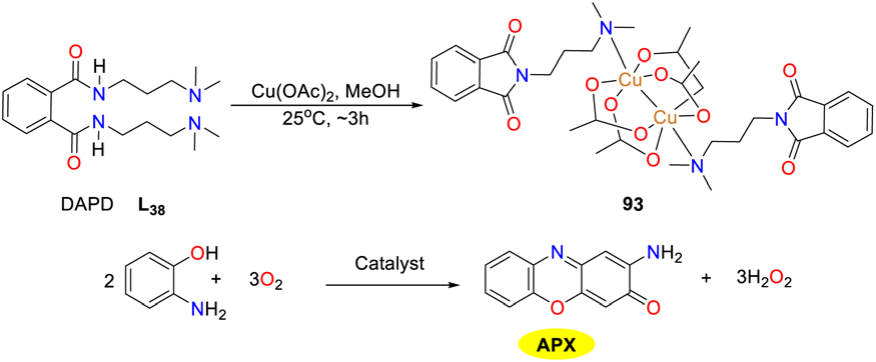
Preparation of complex 93 and the oxidation reaction of OAP to APX (ref. 60).

In 2024, a versatile bioinspired metal catalyst 93 was synthesized and characterized as a distorted octahedral complex by Das and co-workers (Scheme 35).^60^ Literature on paddlewheel-type complexes with catechol oxidase and phenoxazinone synthase activities is scarce; thus, this study addresses this deficiency. The *N*^1^,*N*^2^-bis(3-(dimethylamino)propyl)phthalimide (DAPD) ligand was synthesized to facilitate the formation of a fused heterocycle and the subsequent generation of a paddlewheel complex. Notable antibacterial effects against resistant *E. coli* and *B. cereus*, and promising *in vitro* anticancer activity against HepG2 cells were also observed. Spectroscopic and electroanalytical techniques suggest that the initial oxidation coupling of OAP results in the formation of a catalyst–substrate intermediate. Subsequently, molecular oxygen is activated by the copper center, leading to the formation of hydrogen peroxide and iminobenzoquinone. Ultimately, the reaction of a BQMI intermediate with another OAP yields APX species (*K*_cat_ = 6.6654 × 10^3^ h^−1^).

### 2.4 Other metal complex-catalyzed reactions

**Scheme 36:**
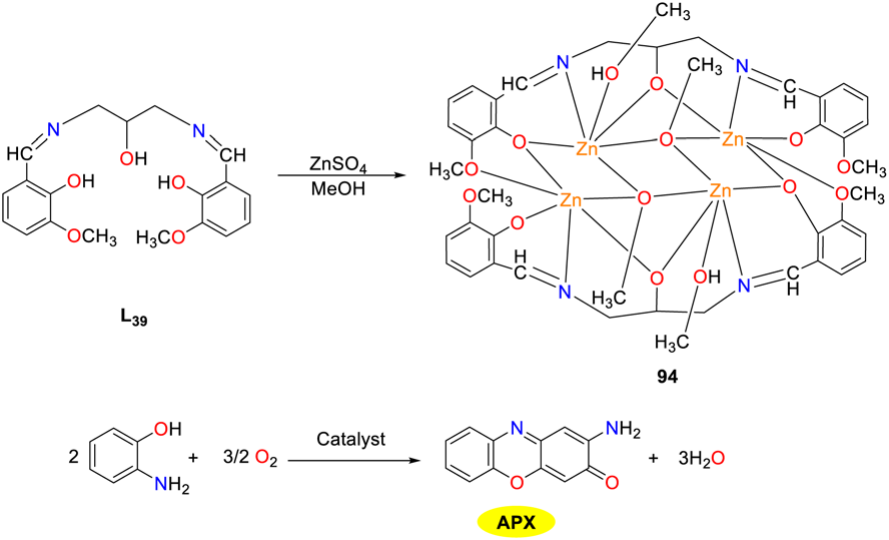
Formation of complex 94 and the oxidation reaction of OAP to APX (ref. 61).

In 2018, the Biswas group reported the synthesis, structural characterization, and catalytic properties of novel tetranuclear zinc(ii) Schiff base complex 94 (Scheme 36).^61^ This compound was notable as the first documented example where a single solvent (CH_3_OH) served as a terminal coordinator bridging agent (μ_3_-CH_3_OH) and crystallization solvent. The zinc(ii) complex exhibited impressive photostability and luminescence with a prolonged lifetime in ethanol. It proved to be an effective catalyst for the oxidation of OAP in ethanol (*K*_cat_ = 6.19 × 10^2^ h^−1^), which was driven by substrate–catalyst adduct formation and radical generation. Notably, imino benzoquinone radicals were rapidly disproportionate and were challenging to detect. This was the first and only instance where a tetrazine(ii)–Schiff base cluster demonstrated the catalytic oxidation of OAP *via* ligand-centered radical activity.

**Scheme 37:**
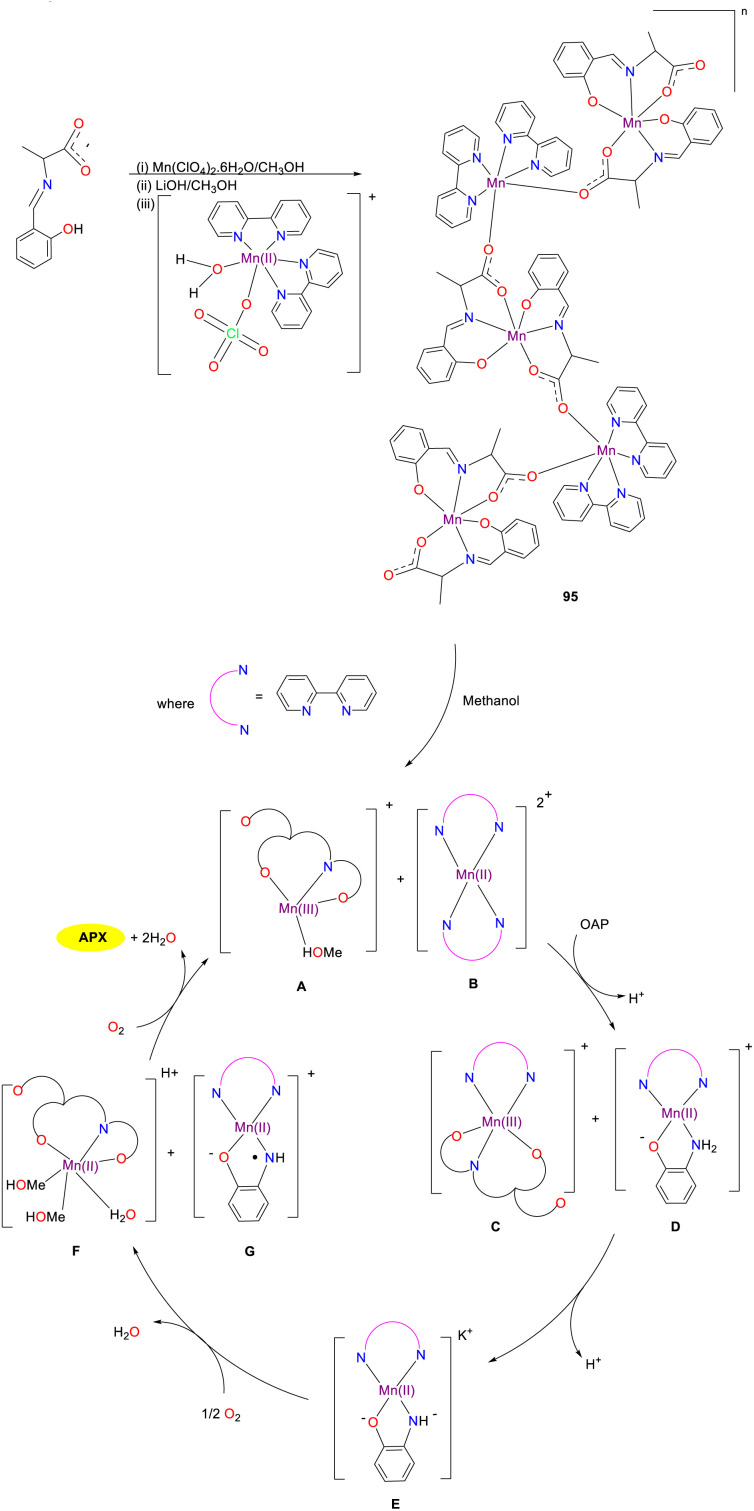
Synthesis of complex 95 and the proposed mechanism (ref. 62).

The first mixed-valence one-dimensional alternating chain composed of Mn(iii) and Mn(ii) bridges by *cis*–*trans* carboxylate 95 was synthesized by the Ghosh group, which exhibited good catalytic activity for the oxidation of OAP to APX (Scheme 37).^62^ According to the mechanism of the reaction, the mixed valence Mn(ii)/(iii) complex first dissociated into its two constituent fragments A and B in the solution, and one of the previously coordinated 2,2′-bipyridine ligands was replaced from the Mn(ii) center and coordinated to the Mn(iii) center, leading to the synthesis of intermediates C and D. Subsequently, the deprotonation of intermediate D resulted in intermediate E, which was further converted into an aminophenol radical G and intermediate F through oxidation processes. Ultimately, the desired product APX is afforded through the regional oxidation of intermediate F with the regeneration of active Mn(iii) complex to accomplish the catalytic cycle. Mn complex 95 proved to be an effective catalyst for the oxidation of OAP through the substrate–catalyst adduct formation and radical generation procedure (*K*_cat_ = 738.0 h^−1^).

**Scheme 38:**
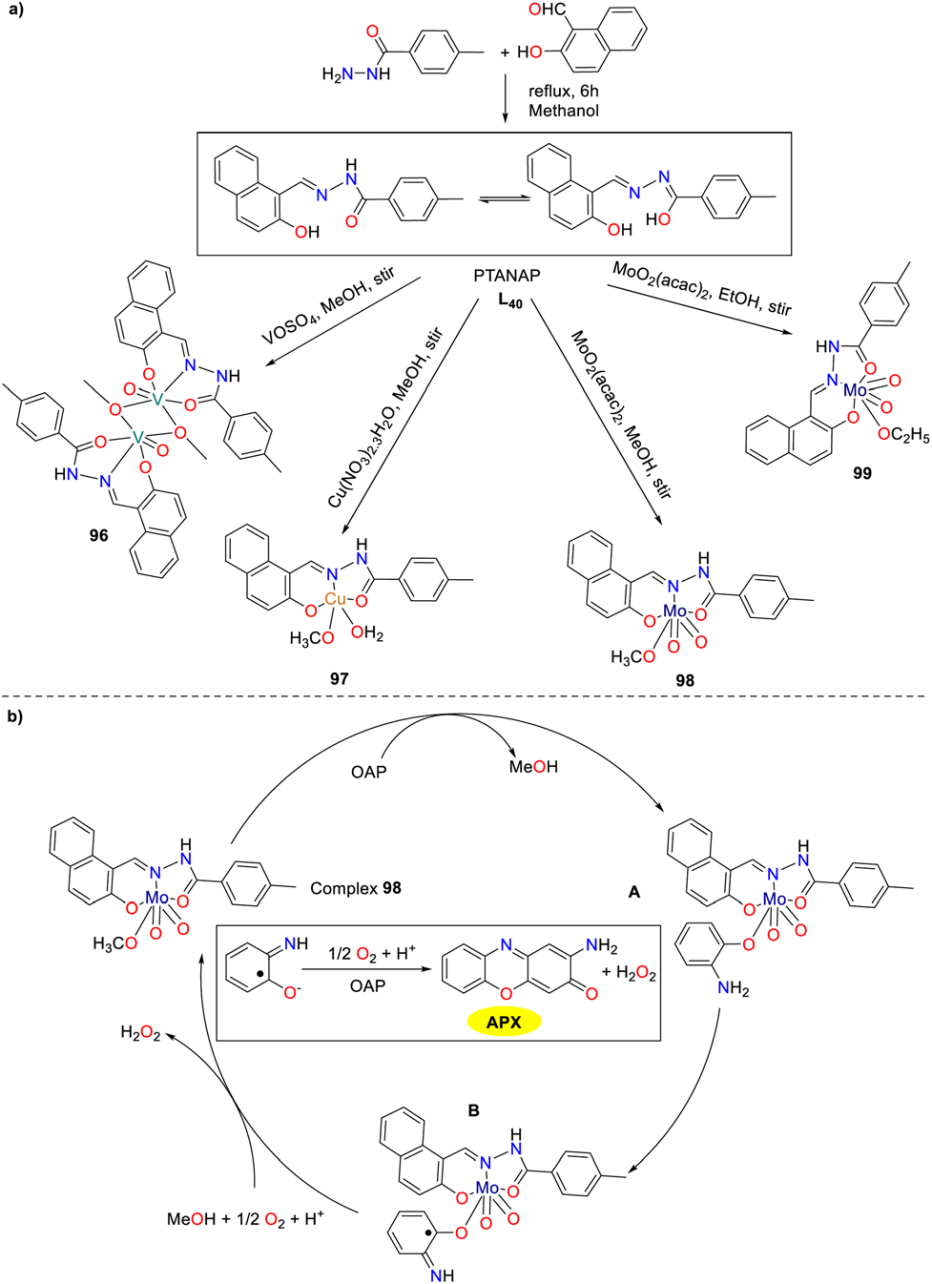
Preparation of complexes 96–99 and the proposed mechanism (ref. 63).

The vanadium complex shows promise for cancer chemotherapy, particularly against leukemia and lymphoma cells with potential therapeutic application in humans. Additionally, metal complexes were extensively utilized as catalysts for the oxidation of ethylbenzene, catechol, and *o*-aminophenol. Building on this, an amide–imine conjugate, (*E*)-*N*′-((2-hydroxynaphthalen-1-yl)methylene)-4-methylbenzohydrazide (PTANAP), and its V(v), Cu(ii), and Mo(iv) complexes 96–99 have been synthesized and characterized by Das and co-workers in 2019 (Scheme 38).^63^ The metal complexes show promising biocatalytic activity, mimicking catechol oxidase (96–97) and phenoxazinone synthase (98–99). This study evaluated the phenoxazinone-synthetic activity of complexes 98 and 99 (the *K*_cat_ values were 6.560 × 10^3^ and 2.464 × 10^3^ h^−1^). Mass spectrometry analysis provided insights into the oxidation of OAP to APX on the basis of molybdenum complex 99. The proposed mechanistic pathway for the phenoxazinone synthase-like activity is illustrated below.

**Scheme 39:**
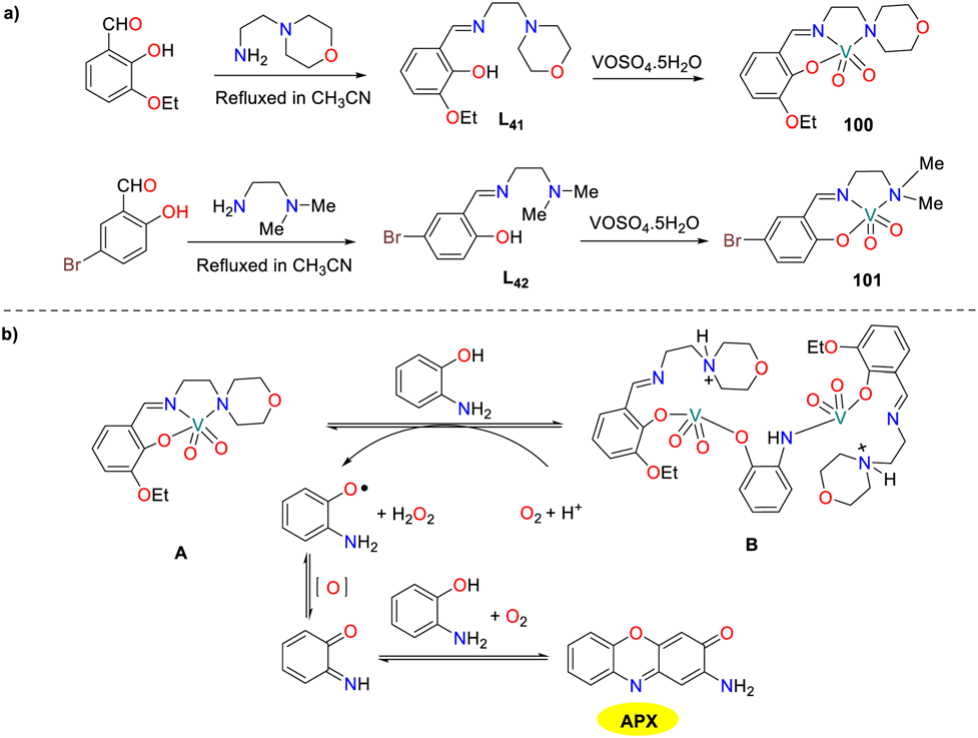
Formation of complexes 100–101 and the proposed mechanism (ref. 64).

In the same year, two structurally analogous vanadium dioxanone complexes 100 and 101 with tridentate N_2_O donor Schiff bases were synthesized and characterized by the Chattopadhyay group (Scheme 39).^64^ Both dioxovanadium(v) complexes displayed phenoxazinone-like synthase activity, enabling them to serve as functional models for the copper-containing enzyme phenoxazinone synthase. The *K*_cat_ values of complexes 100 and 101 were 375.1 and 300.6 h^−1^, respectively. The possible mechanism for the oxidation of OAP to APX is depicted. First, the reaction of OAP with the complex A forms an adduct intermediate B with the breaking vanadium–N (amine) and vanadium–N (imine) bonds simultaneously. Subsequently, the oxidization of intermediate B by molecular oxygen yields an OAP-free radical, which is further converted into the BQMI intermediate through oxidization processes. Finally, the desired product APX was obtained by the reaction of BQMI with another OAP through oxidization sequence.

**Scheme 40:**
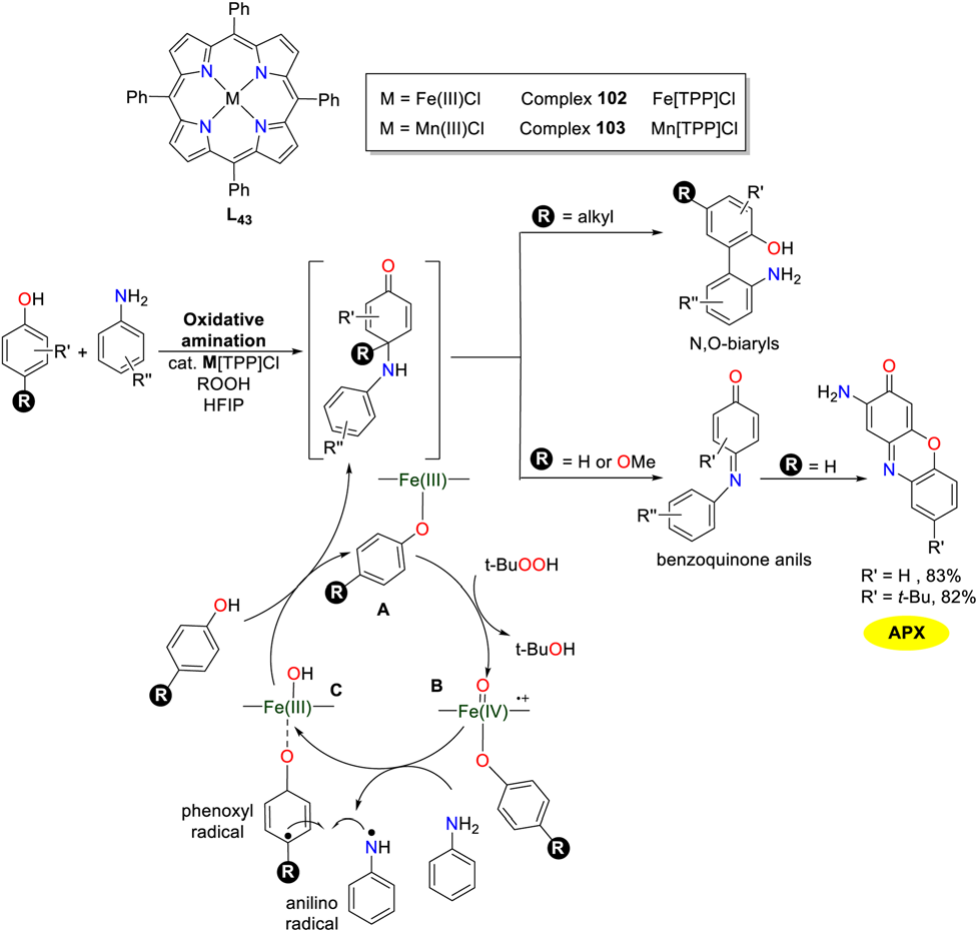
Structures of complexes 102 and 103 and the proposed mechanism (ref. 65).

In 2020, the Pappo group reported high-efficiency *para*-selective oxidative amination of phenols with primary or secondary anilines employing M[TPP]Cl (M = Fe or Mn) complexes 102 and 103 as the catalysts (Scheme 40).^65^ This methodology provided direct access to benzoquinone compounds with *N*,*O*-biaryl structures, which are challenging to obtain through other sustainable approaches. The proposed mechanism entails coupling an aniline radical with an iron-ligated phenoxyl radical. The C–N coupling step generated an unstable anilinoquinone intermediate that might undergo dehydrogenation (R = H), elimination (R = OMe), or [3,3]-sigmatropic rearrangement (R = alkyl) sequences depending on the *para*-substituent of phenol. A series of phenoxazinones have been synthesized by the catalytic system, which were crucial structural motifs in natural products and pharmaceuticals. The synthetic strategy was accomplished through the cross-coupling of 2-aminophenol with 2-amino-(4-*t*Bu)-phenol, yielding the corresponding 2-aminophenoxazinone derivatives in 83% and 82% yields, respectively. A novel catalytic system for chemo-, regio-, and stereoselective oxidative aniline coupling reactions are urgently required in the future.

**Scheme 41:**
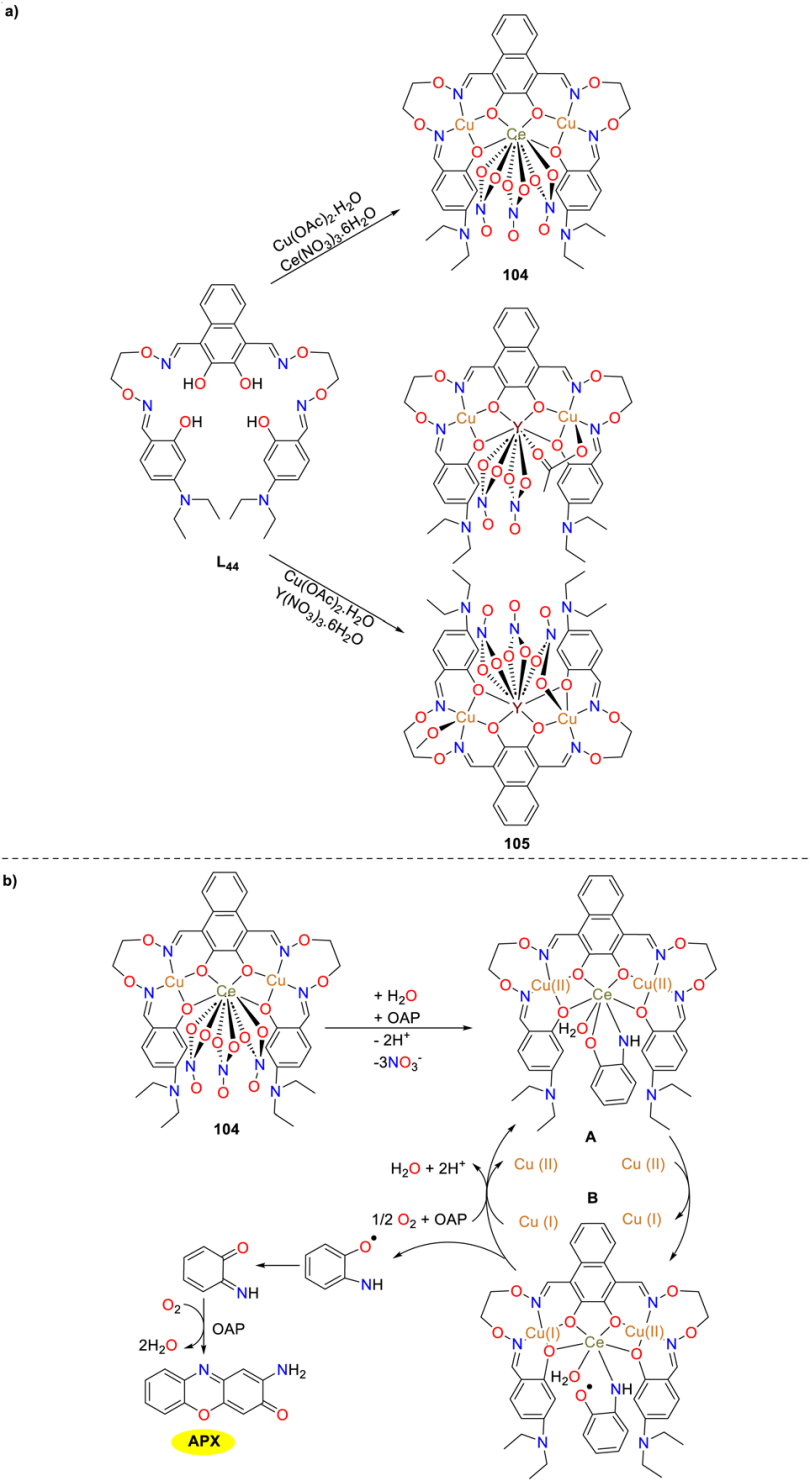
Synthesis of complexes 104 and 105 and the proposed oxidation mechanism (ref. 66).

In the same year, the hetero-trinuclear complex 104 and heterohexanuclear complex 105 were synthesized and thoroughly analyzed by Dong and co-workers (Scheme 41).^66^ The catalytic oxidation properties of these complexes were investigated in depth, which revealed that only complex 104 demonstrated high catalytic activity and exhibited notable catecholase and phenoxazinone synthase-like oxidation capabilities (*K*_cat_ = 9.73 h^−1^). Point spray mass spectrometry analysis determined that the transformation of OAP to APX involved the formation of an *o*-aminophenol radical with complex 104. Furthermore, it was hypothesized that Cu(ii) atoms within complex 104 played a pivotal role as electron transport mediators in the oxidation process. Notably, this protocol represented the first instance of a heteronuclear bis(salamo)-based Cu(ii) complex, facilitating the simultaneous oxidation of 2-aminophenol, providing a valuable reference for future research.

**Scheme 42:**
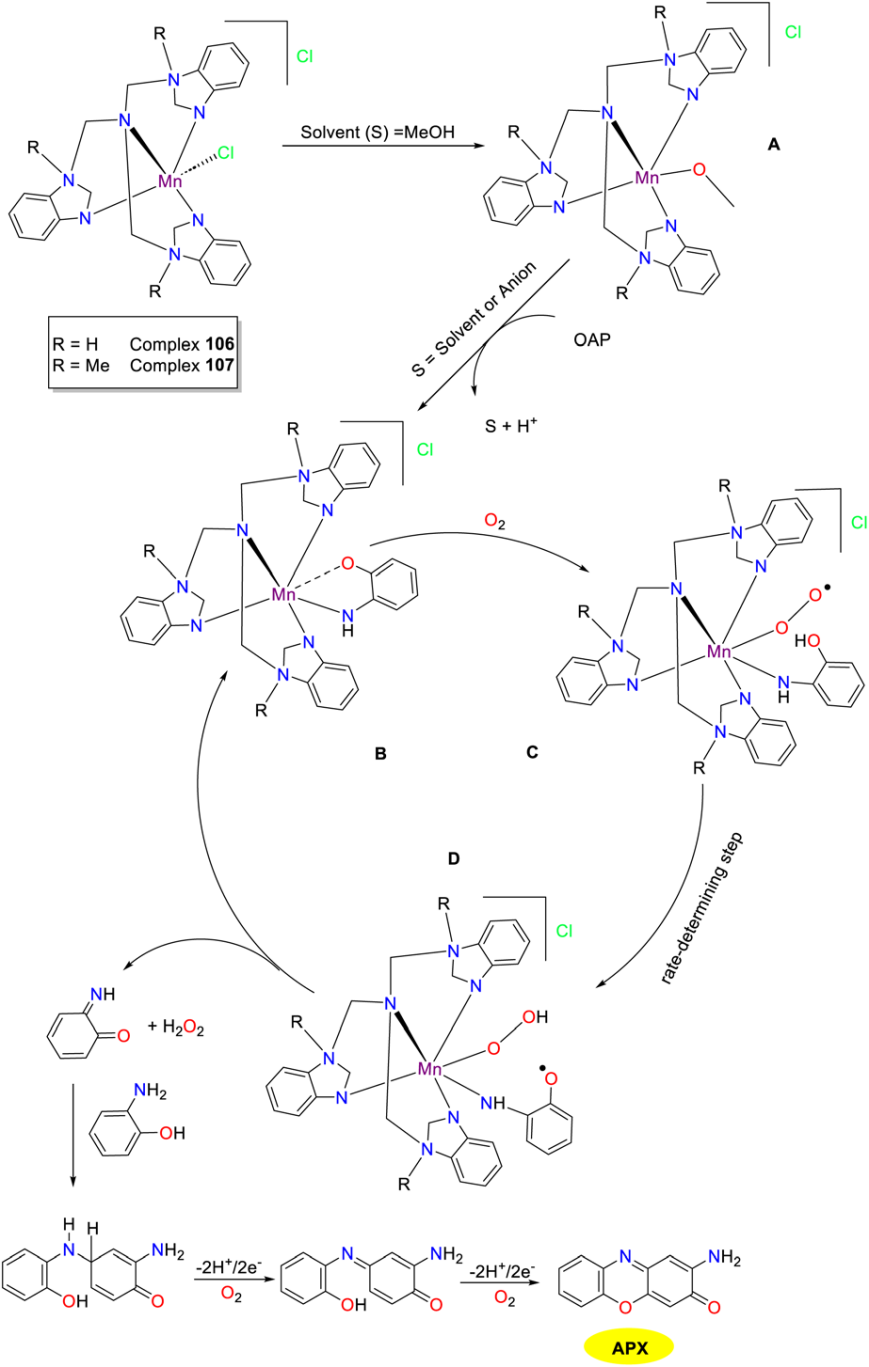
Structures of complexes 106 and 107 and the proposed mechanism (ref. 67).

In 2021, the Maji group synthesized pentacoordinate mononuclear Mn(ii) complexes incorporating two tetradentate benzimidazole-based tripodal ligands, and investigated their function in phenoxazinone synthesis (Scheme 42).^67^ Electrochemical tests revealed the non-innocent nature of the Mn(ii) complexes, and EPR studies confirmed the existence of the high-spin Mn(ii) conformation. Mn(ii) complexes 106 and 107 manifested extraordinary phenoxazinone synthase activity with *K*_cat_ values of 440 h^−1^ and 234 h^−1^, respectively. Based on the ESI-MS spectra data, a mechanism involving hydrogen peroxide as the intermediate is proposed, suggesting the involvement of molecular oxygen in the catalytic process. This comprehensive study illuminated metal–ligand collaboration and phenoxazinone synthase mimicry, providing a novel platform for designing more efficient bioinspired catalysts.

**Scheme 43:**
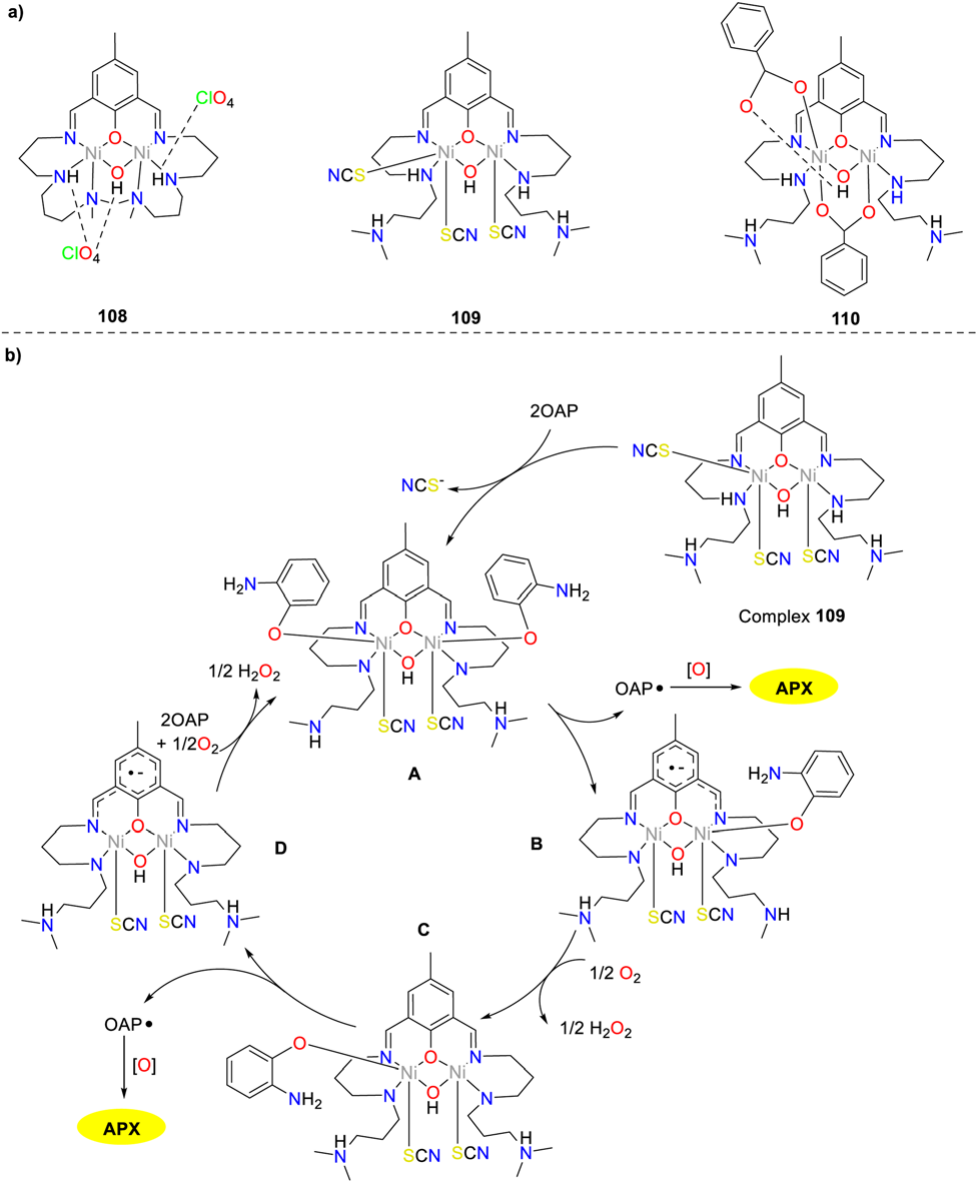
Structures of complexes 108–110 and the proposed mechanism (ref. 68).

In the same year, the synthesis and structural analysis of three dinickel(ii) complexes 108–110 were reported by Panja and co-workers (Scheme 43).^68^ Of note, similar bridged patterns of complex 110 have been documented, the novel combination of aqua, carboxylate, and phenoxy bridges to the dinickel(ii) has not been reported. All three complexes exhibited phenoxazinone-like catalytic activity, which could catalyze OAP to afford APX (the *K*_cat_ values were 12.30, 16.70, and 4.32 h^−1^). It has been established that the efficient catalytic activity was predicated on the presence of labile positions for substrate binding. In dinickel(ii) complexes 108 and 109, the metal centers were found to be coordinated with unsaturated groups or labile thiocyanate ions, which facilitated the coordination of OAP and led to the efficient construction of APX. It is worth noting that the availability of vacant sites and labile positions was crucial for catalytic efficiency. In contrast, the Ni(ii) center was coordinated with monodentate benzoate ions in complex 110, which was stabilized through intramolecular hydrogen bonding, resulting in low substrate conversion due to reduced favorability for adduct formation.

**Scheme 44:**
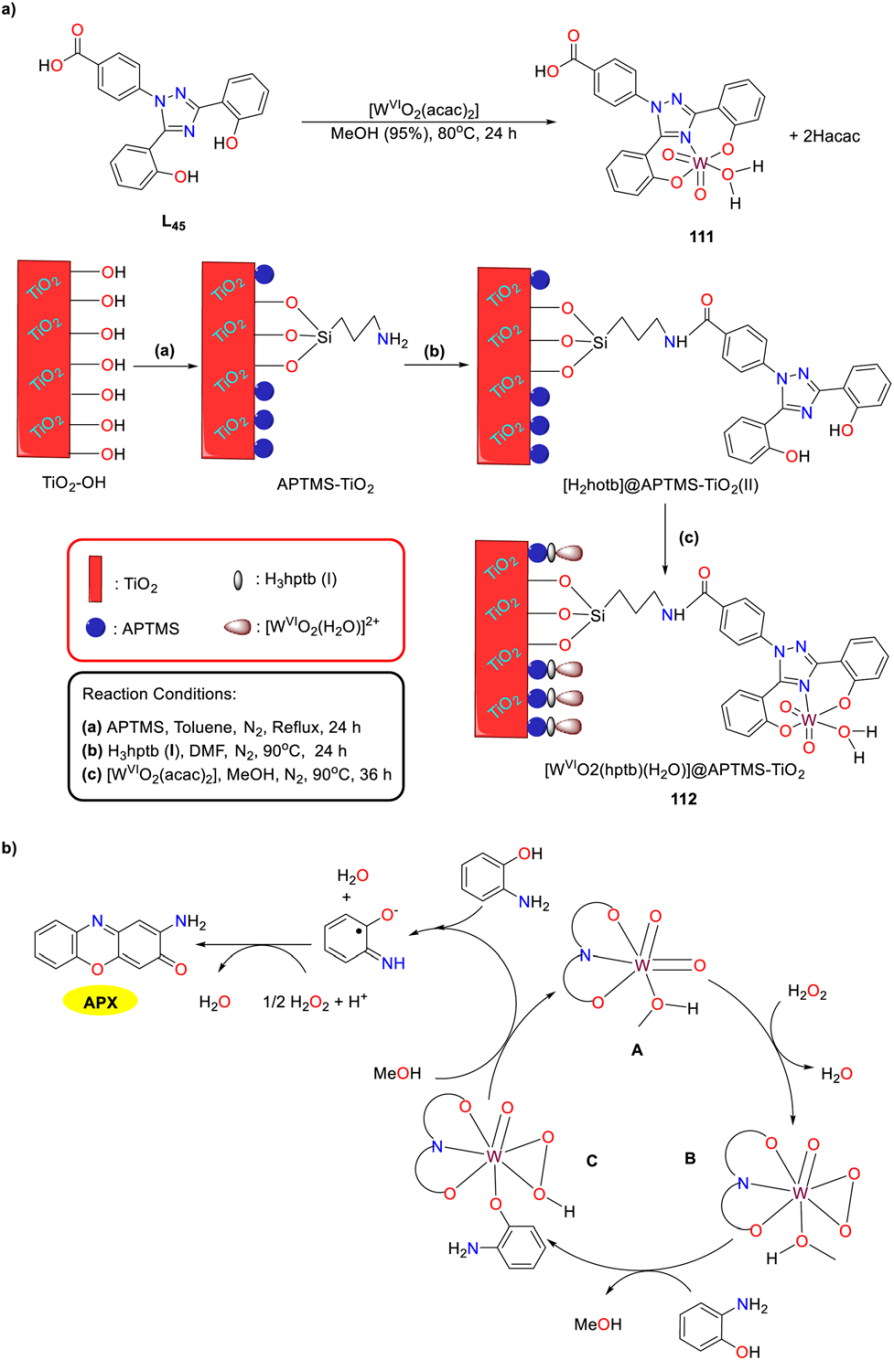
Preparation of complexes 111 and 112 and the proposed mechanism (ref. 69).

In 2023, the synthesis and characterization of the ICL670-based tungsten(vi) complex and its corresponding amine-functionalized heterogeneous titanium dioxide-supported tungsten(vi) complex were accomplished by the Maurya group (Scheme 44).^69^ These complexes were demonstrated to be bio-mimic type II copper–molybdenum oxidases, facilitating phenoxazinone synthase-like copper and molybdenum coordination. Both tungsten(vi) complexes 111 and 112 could catalyze the oxidative coupling of *o*-aminophenol smoothly in methanol under green oxidant H_2_O_2_ conditions to yield 2-aminophenazine-3-one in 80% and 93% yields, respectively. A kinetic analysis of the PHS-like activity was performed on complex 111. The *K*_cat_ value determined from this analysis was 1.18 h^−1^. The tentative mechanism for the tungsten(vi) complex-catalyzed OAP oxidation-coupling reaction is outlined below.

**Scheme 45:**
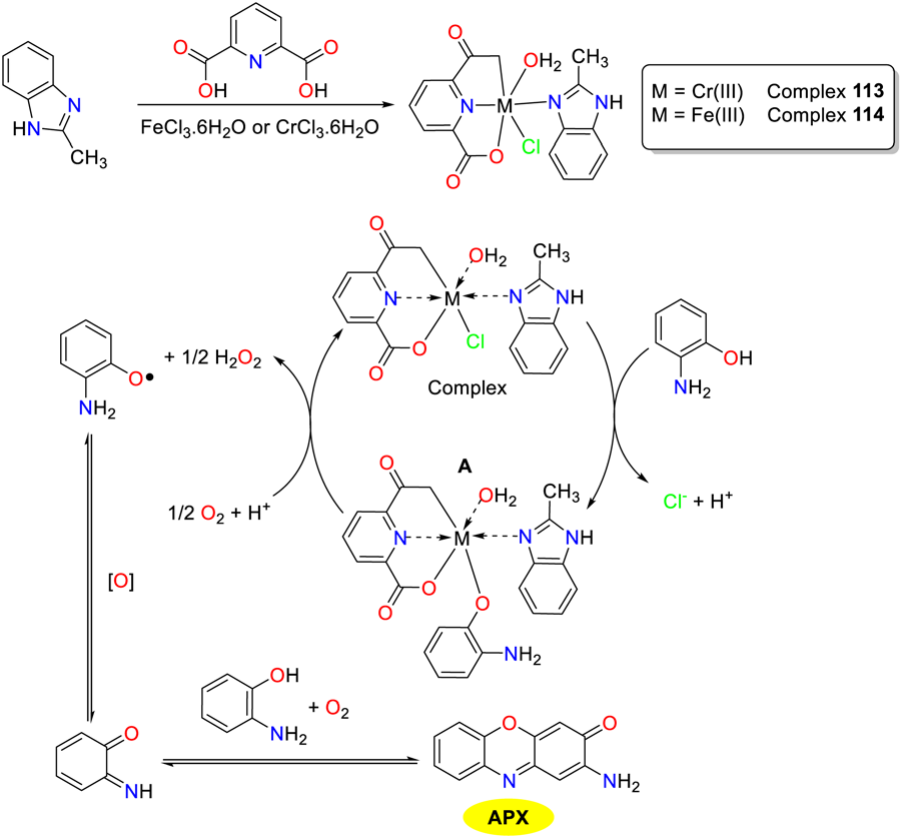
Synthesis of complexes 113 and 114 and the proposed mechanism (ref. 70).

In 2024, the El-Lateef group disclosed the synthesis of two innovative coordination compounds featuring 2,6-pyridine dicarboxylic acid and 2-methyl-1*H*-benzimidazole as dual ligands with chromium(iii) and iron(iii) serving as central ions, respectively (Scheme 45).^70^ Of note, both chromium(iii) complex and iron(iii) complex efficaciously facilitated the oxidative coupling of OAP to yield APX in an acetonitrile medium, demonstrating phenoxazinone synthase-like activity. A tentative mechanism for the assembly of the APX scaffold is outlined below. Initially, the interaction between the OAP molecule and the metal complex results in the formation of an adduct B, which could be further converted into an OAP radical through the oxidation of molecular oxygen. Subsequently, the OAP radical undergoes the second oxidation process and furnishes intermediate BQMI. Finally, the desired product is afforded through the reaction of BQMI with another OAP through OAP-mediated reactions. Employing classical Michaelis–Menten enzymatic kinetic analysis, these complexes exhibited superior *K*_cat_ (the *K*_cat_ values were 208.400 and 177.822 h^−1^, respectively), outperforming previously documented analogs in terms of catalytic efficiency.

**Scheme 46:**
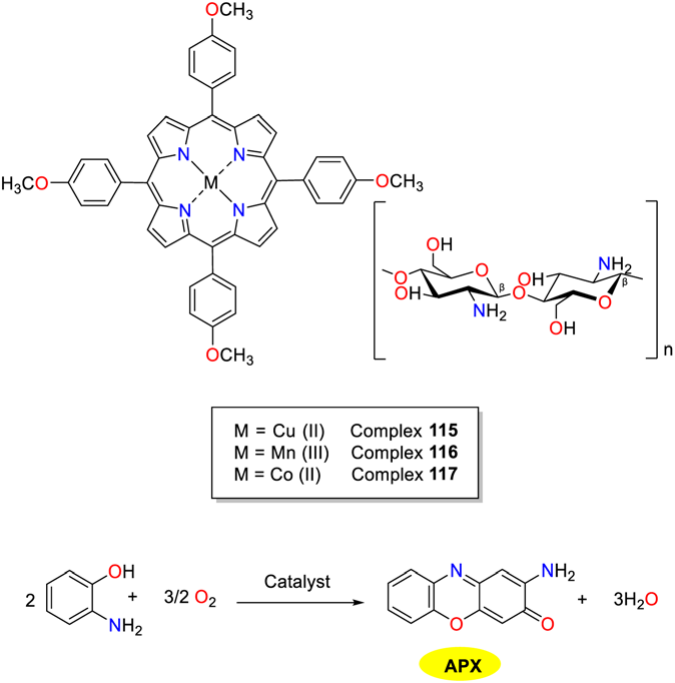
Synthesis of complexes 115–117 and the oxidation reaction of OAP to APX (ref. 71).

In the same year, the El-Khalafy group investigated the catalytic properties of chitosan-supported *tetra*(*p*-methoxyphenyl)porphyrin complexes 115–117 for the heterogeneous activation of OAP to APX in the presence of bicarbonate, mimicking the activity of phenoxazinone enzyme synthase (Scheme 46).^71^ Copper complex 115 supported by chitosan exhibited the highest catalytic efficiency under optimal conditions, achieving an 87% conversion within a 90 minute timeframe. The catalytic performance of copper complex 115 was explored by the investigation of the temperature, bicarbonate concentration, and dissolved oxygen. No superoxide anions were observed as reactive species during the oxidation of OAP catalyzed by copper complex 115 (*V*_max_ = 1.4459 min^−1^ and *K*_M_ = 0.0750 M). It is noteworthy that this catalyst demonstrated high stability and could be recovered from the reaction mixture through simple filtration. Of note, after multiple washes with water, the complex could be recycled for subsequent research without significant changes even after five cycles.

**Scheme 47:**
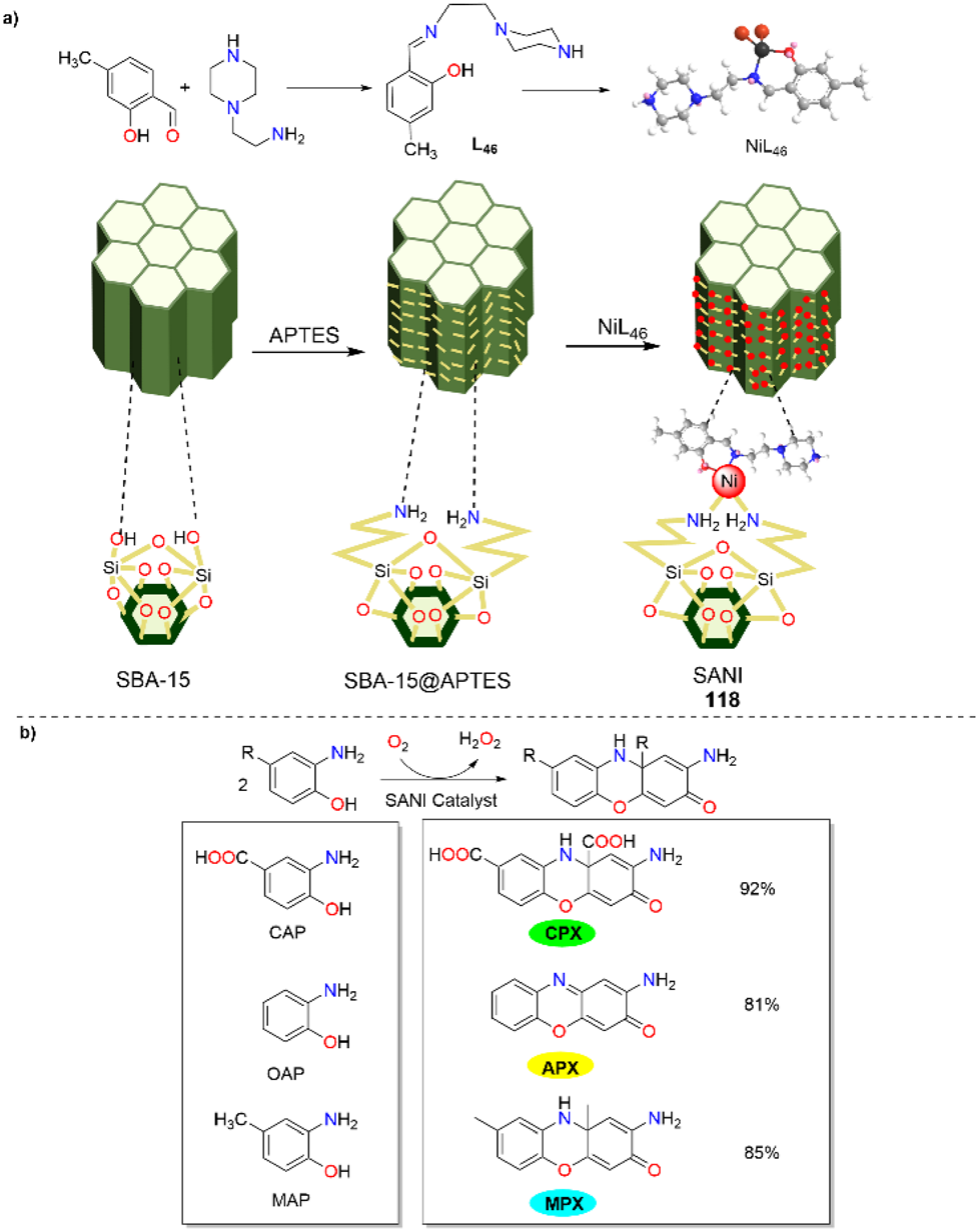
Synthesis of the SANI nanocatalyst and the transformation of OAP to APX (ref. 72).

In 2024, the Bhaumik group unveiled a new mesoporous nanocatalyst known as SANI (118), which was synthesized by modifying a distinct two-dimensional hexagonally ordered SBA-15 surface with a unique Ni(ii) complex (Scheme 47).^72^ This nanocatalyst was employed to produce phenoxazinone derivatives, showcasing its wide applicability. When substituted *ortho*-aminophenol, 3-amino-4-hydroxybenzoic acid, and 2-amino-4-methylphenol were employed as the substrates, the oxidation reactions proceeded smoothly and furnished the corresponding phenoxazinone derivatives in 92%, 81%, and 85% yields, respectively, which demonstrated the potential utility of this mesoporous nanocatalyst. Additionally, SANI exhibited exceptional stability and could be reused multiple times without significant performance loss. The reaction for the formation of phenoxazinone was carried out effectively at room temperature, with the catalyst maintaining its effectiveness throughout several cycles. The outstanding catalytic performance of NiL_46_ active sites and efficient heterogeneous integration on the ordered mesoporous SBA-15 support might be ranked among the most economically viable and sustainable strategies for accessing the APX scaffold.

**Scheme 48:**
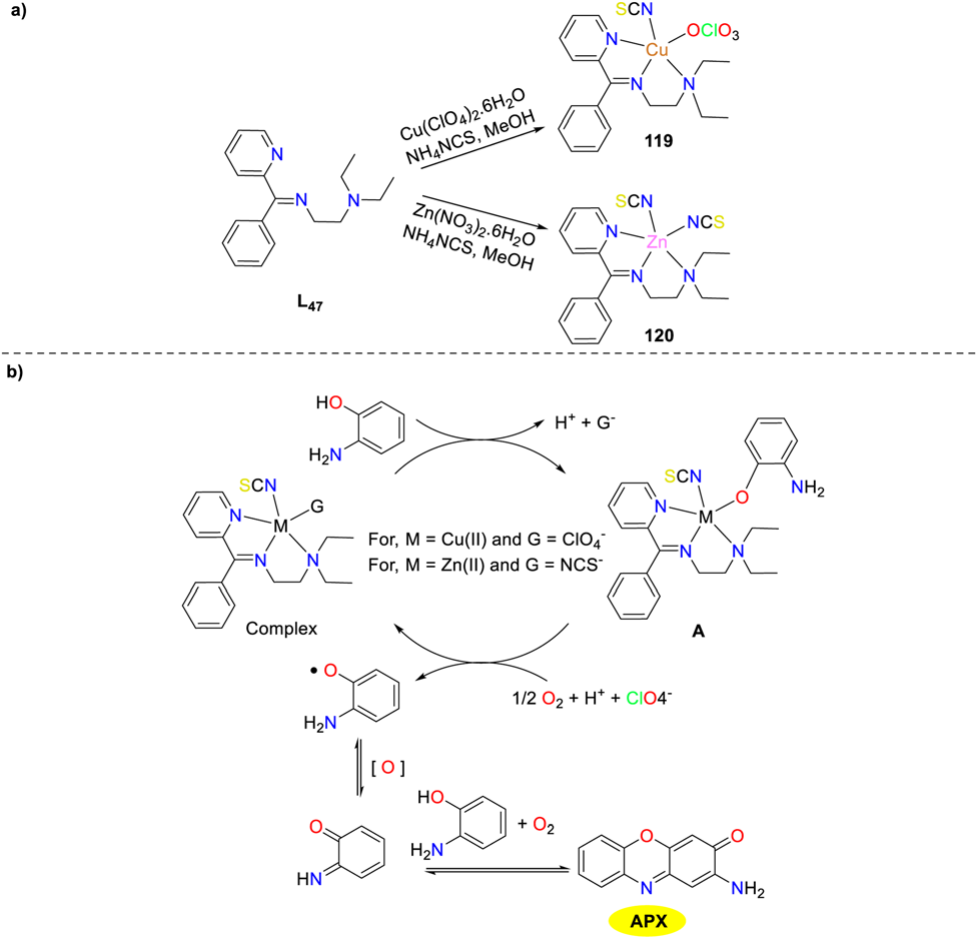
Preparation of complexes 119–120 and the proposed mechanism (ref. 73).

In the same year, neutral Schiff base ligand complexes of Cu(ii) and Zn(ii) 119 and 120 were synthesized and structurally characterized by Ghosh *et al.* (Scheme 48).^73^ Both metal complexes exhibited good phenoxazinone synthase activities, and the catalytic activities of complexes 119 and 120 varied depending on the solvent. Specifically, both complexes 119 and 120 exhibited higher *K*_cat_ in MeOH (*K*_cat_ = 78.28 and 40.00 h^−1^) compared to MeCN (*K*_cat_ = 21.17 and 11.00 h^−1^) and ethyl acetate (*K*_cat_ = 43.06 and 4.00 h^−1^), respectively. The dielectric constant of MeCN is greater than that of MeOH, and the affinity for association or complex formation between the complexes and OAP is higher in MeOH than in MeCN. This, in turn, accounts for the higher *K*_cat_ in MeOH than in MeCN. However, since solvents with higher polarity have better stabilizing effects on free radicals, the phenoxazinone synthase activity is higher in MeOH, which has a higher polarity than that of ethyl acetate. This study provides detailed kinetics and mechanistic insights into these catalytic activities.

### 2.5 Multiple metal complex-catalyzed reactions

**Scheme 49:**
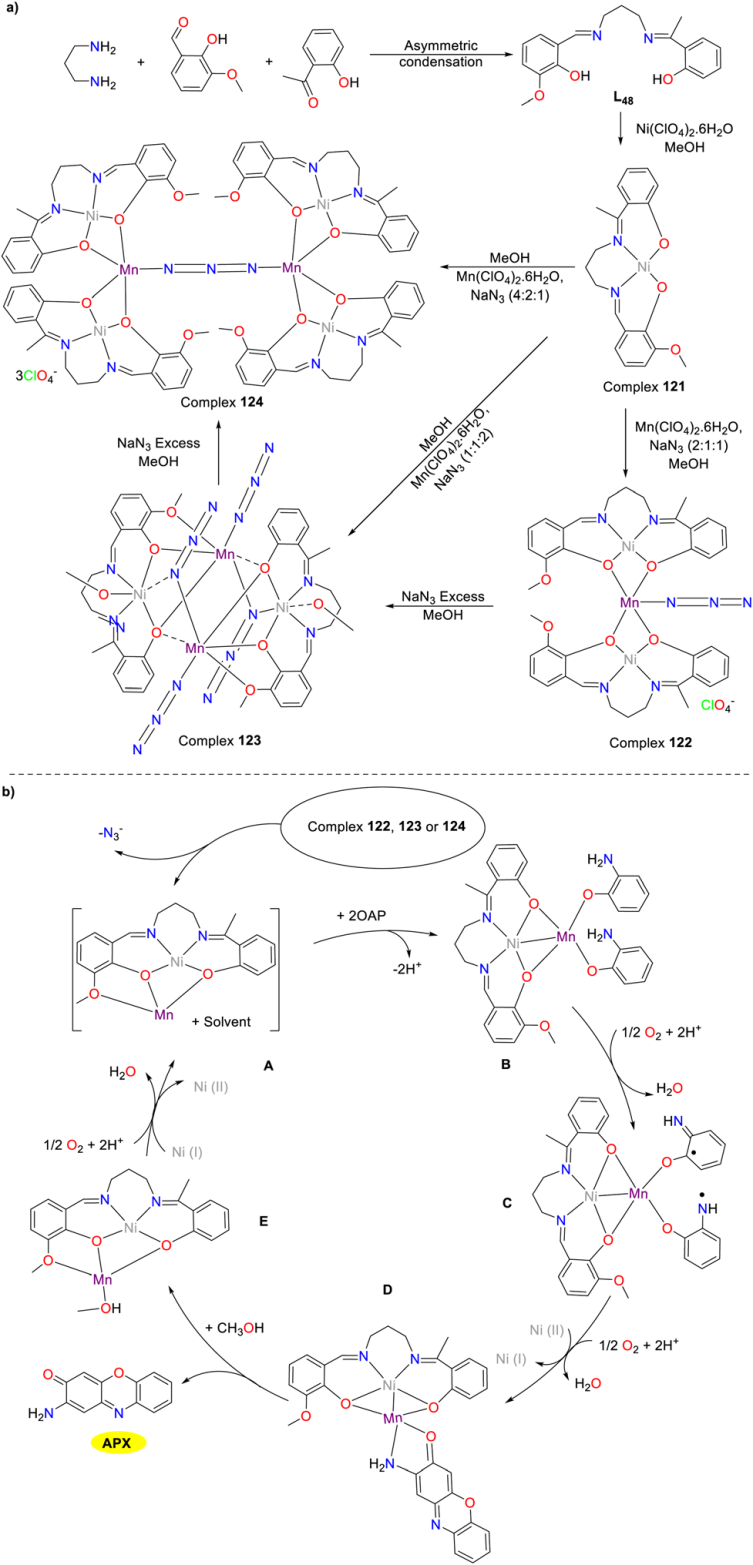
Synthesis of complexes 121–124 and the proposed mechanism (ref. 74).

In 2018, the Ghosh group synthesized a novel mononuclear complex 121 by employing an asymmetric Schiff base ligand with N_2_O_3_ as a donor to advance the chemistry of metal–ligand bimetallic compound synthesis (Scheme 49).^74^ Various trinuclear complex 122, tetranuclear complex 123, and hexagonal complex 124 could be synthesized through the reaction of complex 121 with manganese perchlorate and sodium azide by varying the ratios of reagents. Complexes 122–124 have been demonstrated to possess phenoxazinone synthase activity. The *K*_cat_ values were 3240, 3360, and 13 248 h^−1^ for complexes 122–124, respectively. The differences in the *K*_cat_ values of the complexes may be related to the number of (NiL_48_) Mn^2+^ units produced per molecule. The three donor oxygen atoms of the asymmetric N_2_O_3_ donor Schiff base-derived metal-ligands seemingly played an extremely significant role in stabilizing the active species. Additionally, the Mn(ii) center in these complexes formed a tuneable unsaturated five or six coordination with the solvent molecule, which facilitated the coordination between the substrate and the Mn(ii) center, thereby further influencing the catalytic efficiency.

**Scheme 50:**
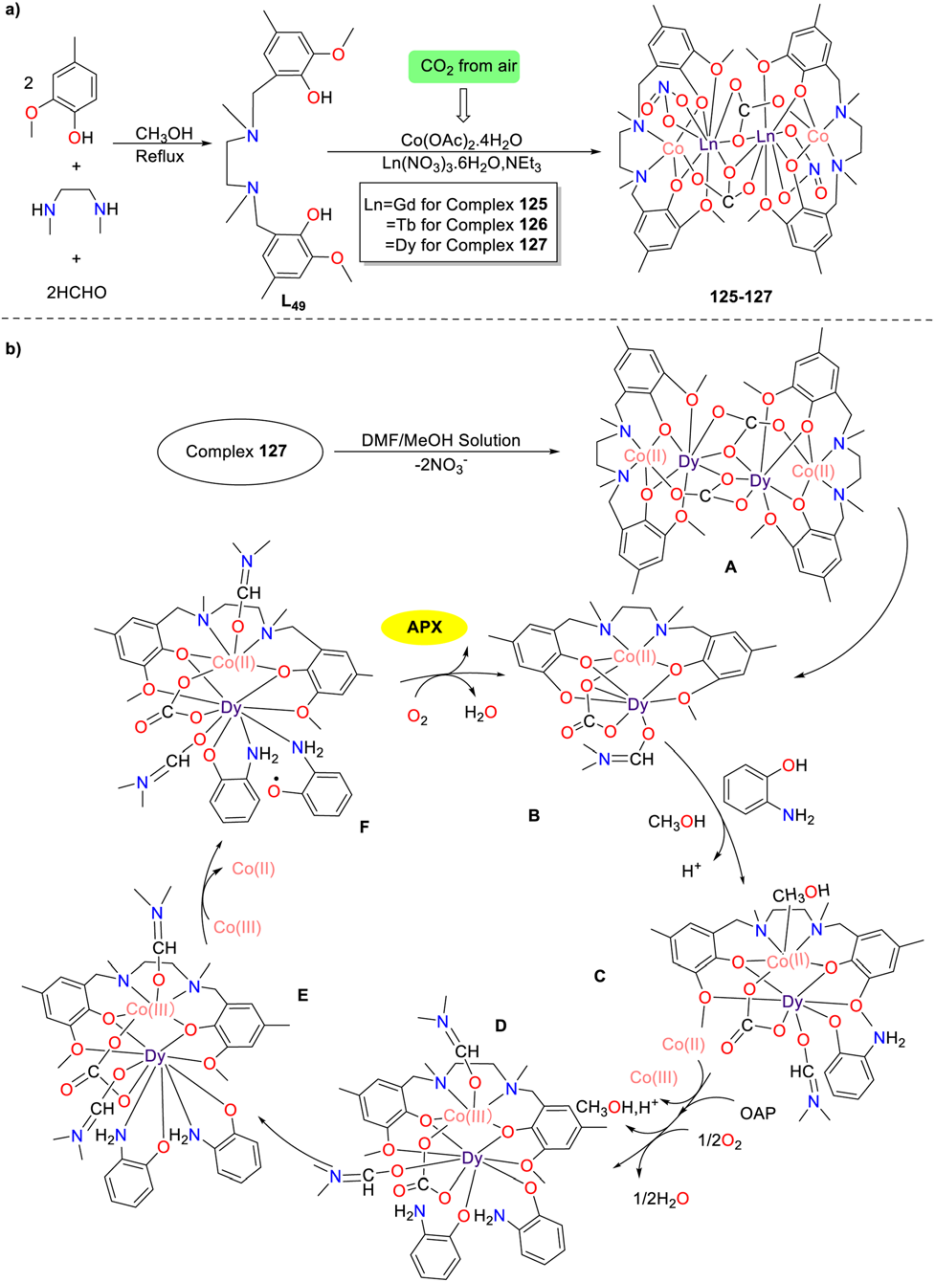
Synthesis of complexes 125–127 and the proposed mechanism (ref. 75).

In 2019, the first example of carbonate bridged tetranuclear Co(ii)–Ln(iii) complexes 125–127 was synthesized by Ghosh and colleagues, and those multiple complexes exhibited considerable phenoxazinone-synthase-like activity; the *K*_cat_ values were 2930.6, 2965.2, and 2998.5 h^−1^ for complexes 125–127, respectively (Scheme 50).^75^ Taking complex 127 as an example, the possible mechanism is depicted below. Initially, the complex 127 furnishes intermediate A in a DMF/methanol solution, which is further converted into catalytically active binuclear B through a cleavage reaction. Subsequently, the coordination of *o*-aminophenol with intermediate B produces intermediate C, which is further transformed into intermediate D by reacting with another *o*-aminophenol molecule through an aerobic oxidative process with the release of water. Next, the coordination of the nitrogen atom of OAP with the Dy(iii) metal ion produces intermediate E, which undergoes an oxidative process by Co(iii) metal ions and affords *o*-aminophenol radical F. Finally, the desired product APX is afforded *via* the aerobic oxidation with the regeneration of the active intermediate B. A particularly noteworthy aspect of complex synthesis is the fixation of atmospheric carbon dioxide into carbonates to form multiple complexes.

**Scheme 51:**
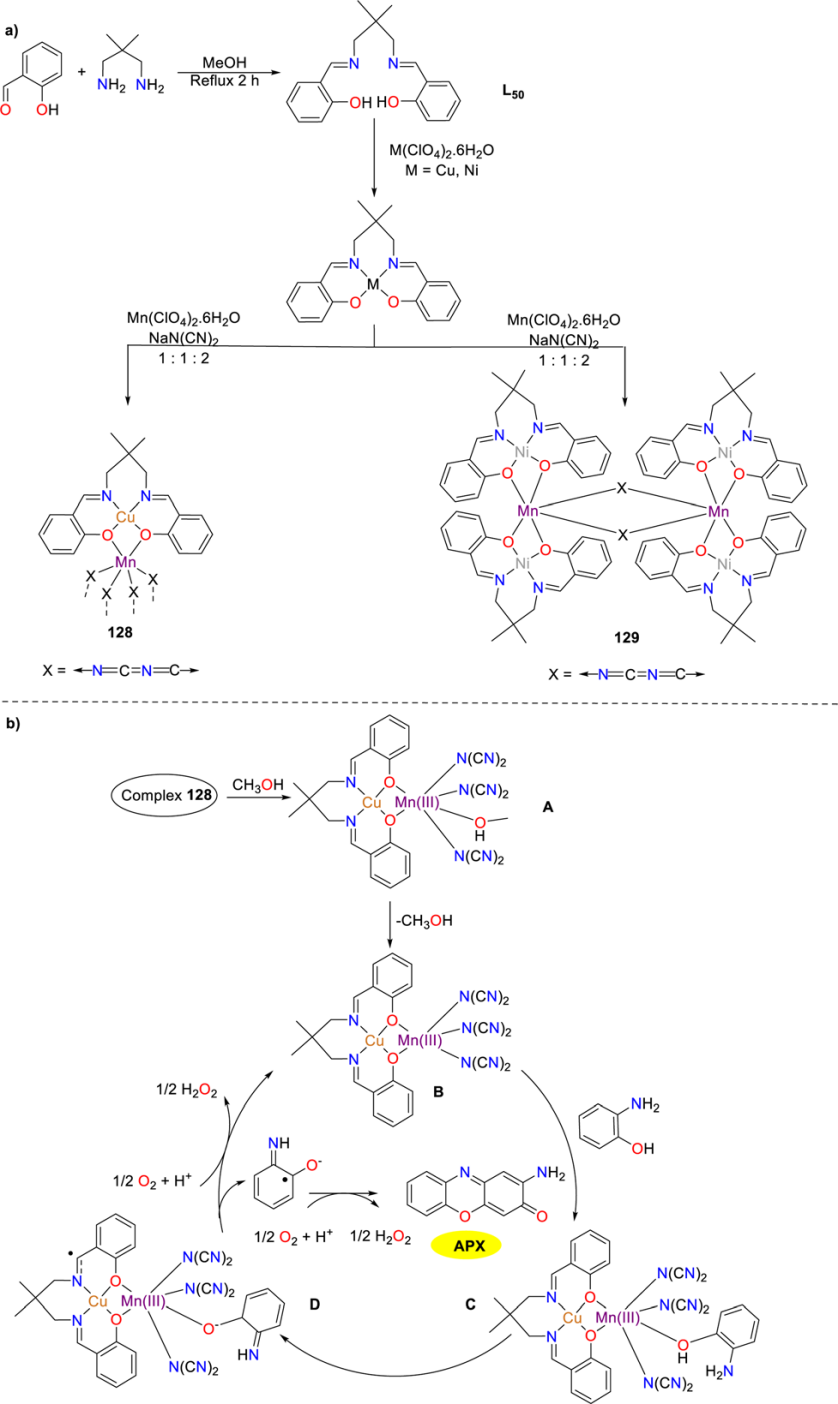
Synthesis of complexes 128–129 and the proposed mechanism (ref. 76).

Two heterometallic Schiff base complexes 128 and 129 were synthesized and characterized by the Das group in the same year (Scheme 51).^76^ Cu–Mn complex 128, which was synthesized employing the N_2_O_2_ donor ligand and a dicyanamide spacer as the substrates, has been characterized as a one-dimensional zigzag polymeric structure. In contrast, Mn–Mn complex 129 is distinguished by a discrete hexanuclear structure. The catalytic activity towards the oxidation of *o*-aminophenol was exhibited by complex 128, which demonstrated a significantly high *K*_cat_ value (5129 h^−1^) compared to other heterometallic Schiff base compounds reported in the literature. This high *K*_cat_ value reflected the enhanced efficiency and catalytic activity of the heterometallic catalyst in the transformation. Nevertheless, Mn–Mn complex 129 was found to retain its geometric structure in the solution, which resulted in its inability to form a compound–substrate intermediate during the catalytic reaction.

**Scheme 52:**
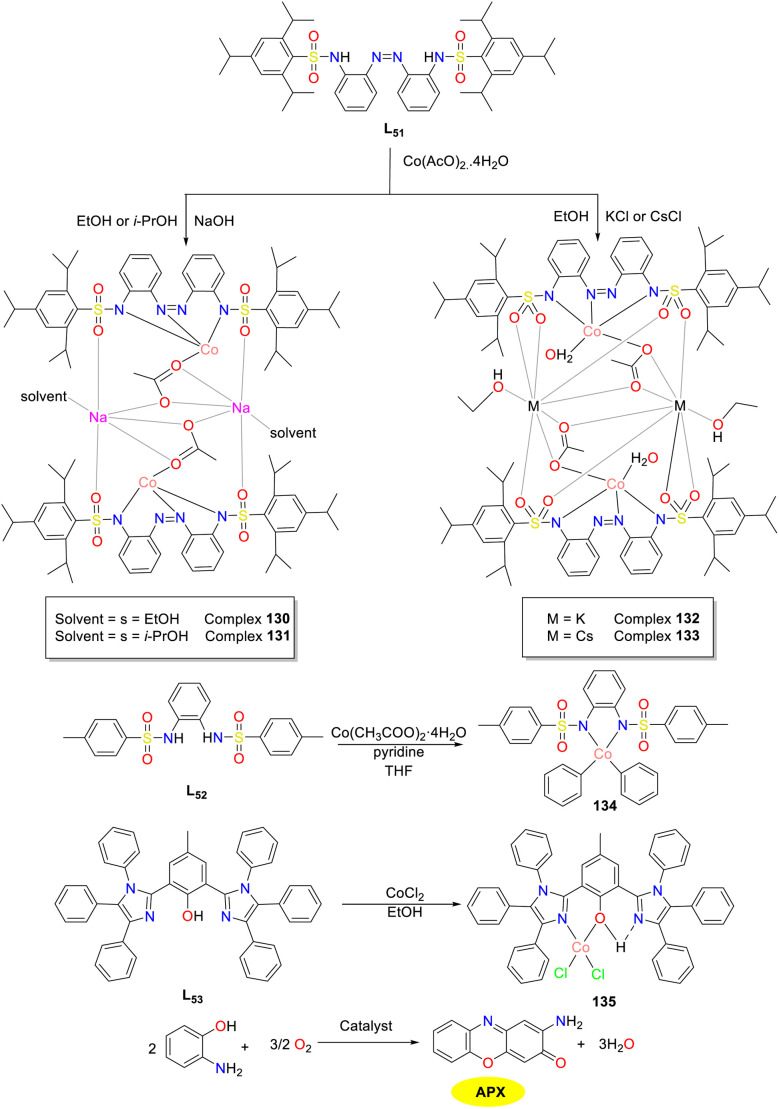
Synthesis of complexes 130–135 and the oxidation reaction of OAP into APX (ref. 77).

In 2020, Eseola and colleagues advanced the development of synthetic phenoxazinone synthase models and investigated the impact of a coordination environment and molecular factors on biomimetic activity (Scheme 52).^77^ Three distinct ligands L_51_–L_53_ were synthesized, which were further combined with cobalt acetate monohydrate to furnish four heteronuclear complexes 130–133, demonstrating diverse levels of phenoxazinone synthase-like activity. Of note, enhanced ligand chelation was associated with decreased catalytic efficiency. The similar catalytic efficiency of complexes 130–133 indicated that different groups of complexes did not significantly impact catalytic activity, suggesting that L_51_ might be the primary active agent. Consequently, it is recommended to combine the anionic chelating ligand with readily separable neutral co-ligands at the cobalt(ii) center rather than neutral chelating ligands paired with monoanionic co-ligands. Additionally, cobalt complex 134 with a bidentate ligand was less effective than complex 130 with a tridentate ligand, suggesting that coordinative saturation may be detrimental. The halogen donor in complex 135 impeded substrate binding, which could be mitigated through the removal of chloride employing potassium or cesium carbonate.

**Scheme 53:**
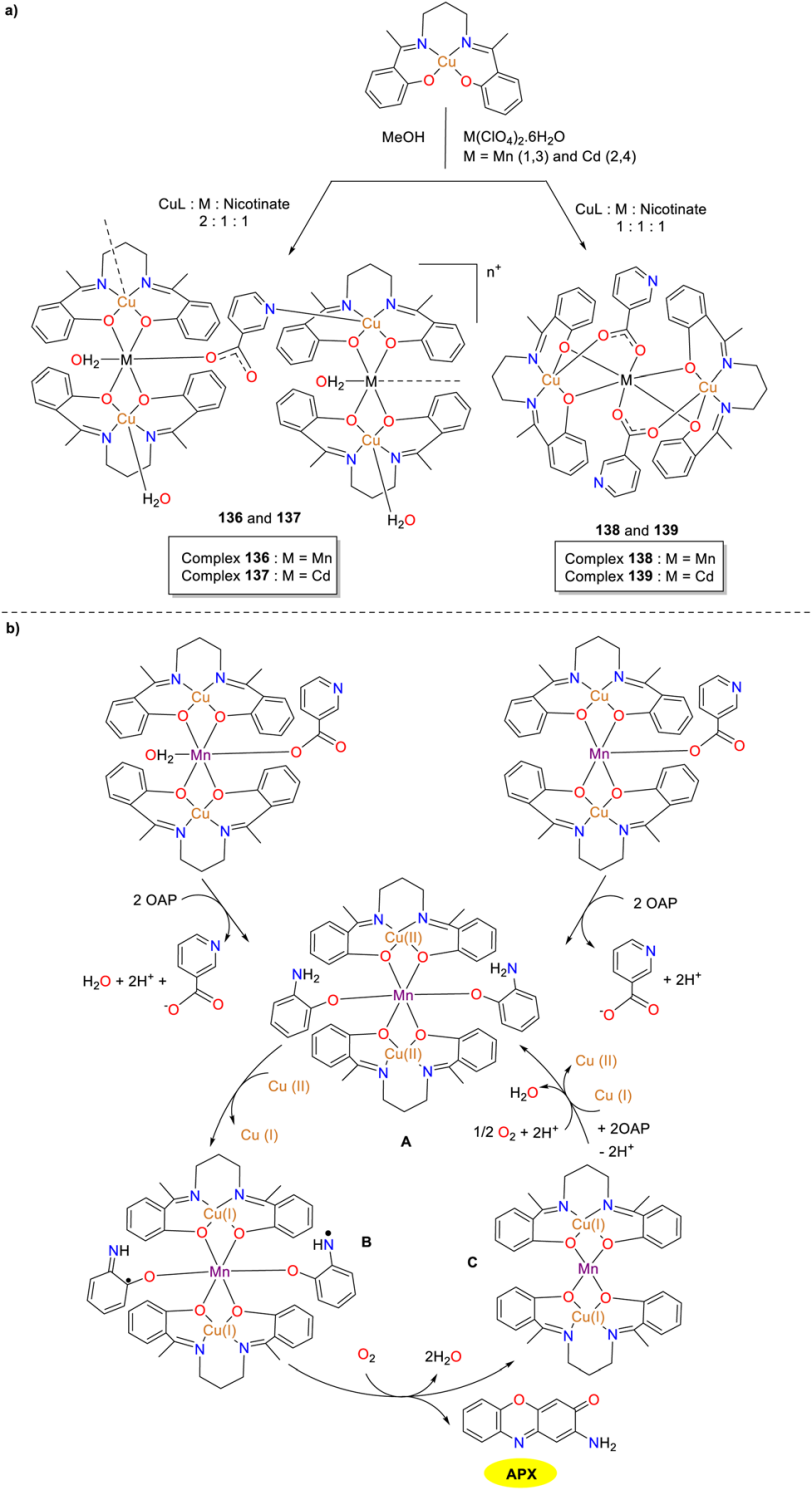
Synthesis of complexes 136–139 and the proposed mechanism (ref. 78).

In the same year, the reaction of copper complex, perchlorate, and nicotinate at a 2/1/1 stoichiometric ratio was carried out by Ghosh and co-workers, leading to the formation of two one-dimensional metal complexes 136 and 137 (Scheme 53).^78^ When this ratio was changed to 1/1/1, two alternative complexes 138 and 139 possessing a unique trinuclear architecture were obtained. Of note, only multiple metal complexes 136 and 138 were actively involved in the catalytic cycle exhibiting phenoxazinone synthase activity. In contrast, the functionality of complexes 137 and 139 where manganese(ii) was substituted with cadmium(ii), remained inactive. The *K*_cat_ values were 300 and 356 h^−1^ for complexes 136 and 138, respectively. This disparity in effectiveness emphasized the crucial role of water molecule, which could coordinate with the central Mn(ii) complex 136 and facilitate substrate attachment. Nevertheless, the catalytic incapability of complexes 137 and 139 suggested that manganese(ii) might not only assist in substrate binding but also participate in crucial electron transfer and intermediate stabilization processes. To further verify the proposition, additional synthesis of complexes involving the substitution of manganese(ii) with a variety of transition and non-transition metal is necessary.

**Scheme 54:**
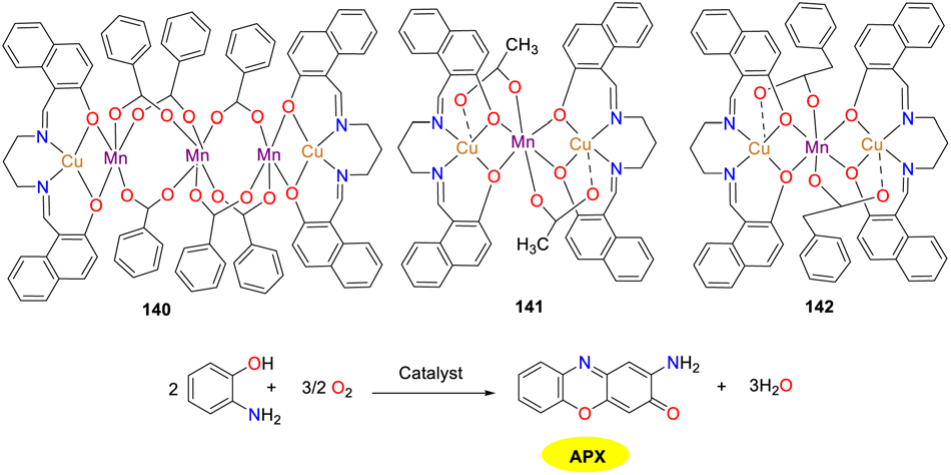
Structures of complexes 140–142 and the oxidation reaction of OAP to APX (ref. 79).

In 2020, the Ghosh group synthesized three novel hetero-metallic copper(ii)–manganese(ii) complexes 140–142, which all exhibited catalytic activity in oxidase reactions (Scheme 54).^79^ The carboxylate ion, which served as the conjugate base, could facilitate the attachment of catechol/*o*-aminophenol to the catalyst through acceptance of proton. The catalytic efficiency of the three complexes was discrepant despite the similar reduction potential of Cu(ii) to Cu(i). The *K*_cat_ values were 25, 4, and 11 h^−1^ for complexes 140–142, respectively. The higher catalytic performance of complex 140 is probably attributed to the greater number of [(CuL)Mn]^2+^ units per molecule. On the contrary, the lower activity of complex 141 is influenced by multiple factors such as p*K*_a_ of carboxylic acid, steric effects around the metal center, and stability of the intermediates.

## 3. Construction of 2-aminophenoxazinone compounds catalyzed by biosynthetic enzymes

Enzyme-catalyzed reactions and other biosynthetic methods align with the principles of green chemistry, resulting in an increasing interest among researchers in both academic and industrial communities. In a natural environment, microorganisms could synthesize phenoxazinone skeletons through enzymatic processes, and phenoxazinone synthase is widely distributed across various species. The rapid and efficient metabolic pathways enable the swift production of target compounds, and these metabolic routes can be precisely regulated through genetic engineering techniques, which present significant advantages as biosynthetic platforms.^80,81^ Of note, the cultivation conditions for microorganisms are relatively simple and cost-effective, making them well-suited for large-scale production. In addition, their green synthesis processes have minimal environmental impacts, which demonstrate considerable potential for application in modern industry and biotechnology.

**Scheme 55:**
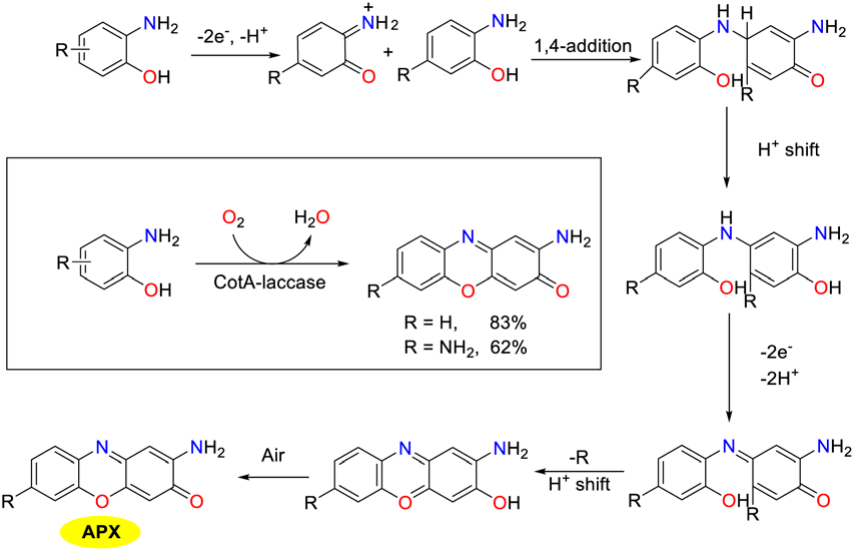
Proposed pathway for the oxidation of OAP to APX (ref. 82).

In 2014, the Robalo group demonstrated that CotA-laccase from *Bacillus subtilis* could oxidize various aromatic amines such as *ortho*-phenylenediamines, *para*-phenylenediamines, and *ortho*-aminophenols, to yield substituted phenazine and phenoxazinone derivatives (Scheme 55).^82^ The method presents an environmental approach for the formation of phenazines and phenoxazinone frameworks with broad applications. Laccase-catalyzed reactions were performed in aqueous solvent systems under mild conditions with water as the only by-product. The proposed mechanism involves the initial single electron oxidation of *ortho*-diamine or *ortho*-aminophenol precursors, producing *ortho*-quinone-diimine or *ortho*-quinone-imine intermediates. Subsequently, these intermediates undergo 1,4-conjugated Michael addition to furnish coupling intermediates, followed by sequential protolysis, and oxidation sequences lead to the formation of the desired products.

**Scheme 56:**
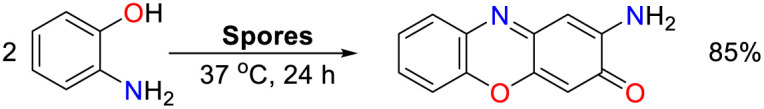
Oxidation of OAP to form APX by the spore system (ref. 19).

In 2020, the Hu group investigated the use of *Bacillus amyloliquefaciens* spore as an alternative to laccase for organic synthesis, leveraging the high density of CotA-laccase present on the spore surface (Scheme 56).^19^ The spores demonstrated efficacy in the cross-coupling of substituted aromatic amines such as 2-aminophenol, achieving yield up to 85% for the corresponding phenoxazinones. The spore-based oxidation system furnished the desired products in good yields with notable selectivity. The synthetic advantages of the protocol were further highlighted by an inexpensive catalytic system, excellent thermal stability, and reusability of spores, which could open up a sustainable and environmentally friendly strategy for the generation of 2-aminophenoxazinone scaffolds. Additionally, the catalytic system operated with oxygen as the oxidant and water as the medium, obviating the need for enzyme purification or immobilization.

**Scheme 57:**
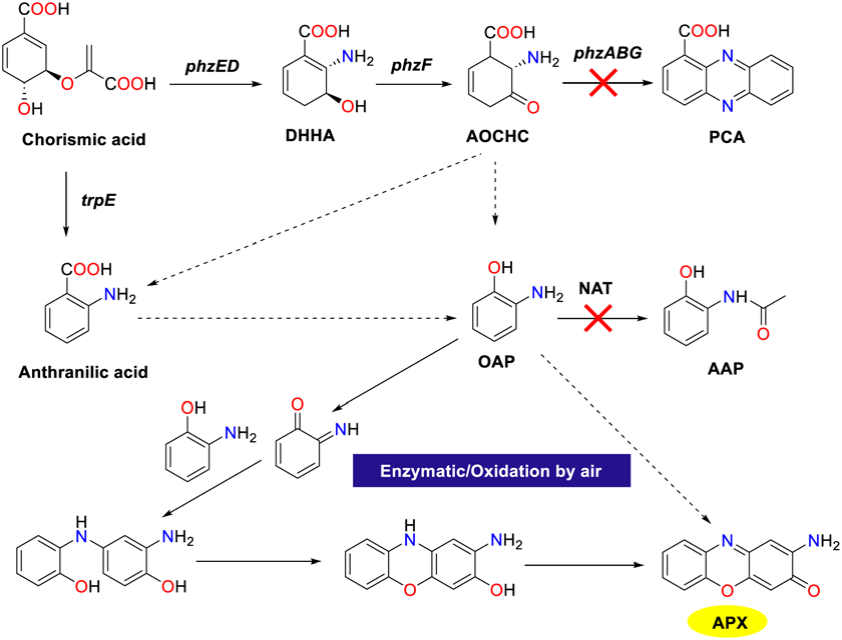
Proposed biosynthesis pathway of APX in HT66 (ref. 83).

In 2022, Zhang *et al.* identified APX in *P. chlororaphis* HT66ΔphzBΔNat and demonstrated that it shared a biosynthetic pathway with PCA (Scheme 57).^83^ By employing rational metabolic engineering techniques including pathway blockage, promoter engineering, gene integration site screening, and heterologous phenoxazinone synthase introduction, the production of APX in *P. chlororaphis* reached the highest 589.78 mg L^−1^ titer. Of note, 2-aminophenol could be converted into AAP by NATs in HT66, while in HT66ΔphzBΔNat, the lack of NAT disrupted AAP production, causing the 2-aminophenol accumulation. Since APX production was reduced in HT66ΔphzBΔNat compared to HT66ΔphzB, phenoxazinone synthase was absent in HT66, which suggested that 2-aminophenol could spontaneously condense to form APX under aerobic conditions. This research advances the understanding of APX biosynthesis and suggests high-efficiency strategies for developing new antibacterial biopesticides.

## 4. Synthetic process routes

In practical production, it is essential not only to develop novel and effective synthesis methods but also to implement the strategies within the production process. Consequently, this review offers a comprehensive introduction of two efficient synthetic processes for phenoxazinone. These processes achieve continuous production in alignment with green chemistry principles while demonstrating excellent substrate adaptability, enabling the synthesis of phenoxazinone skeletons with various substituents. The environmentally friendly characteristics of these processes not only mitigate environmental impacts but also enhance the economic viability and sustainability of production. By optimizing synthesis conditions, these approaches fulfill production requirements while improving the overall efficiency, thereby providing reliable technical support for practical applications.

**Scheme 58:**
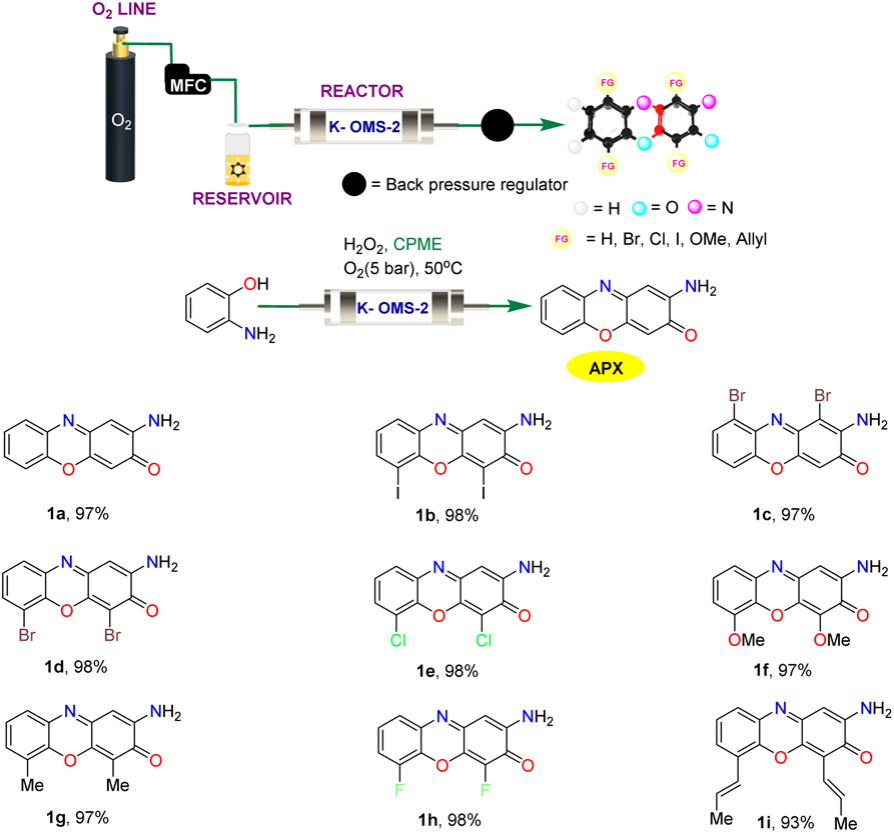
Flow reactor and scope for the synthesis of APX (ref. 22).

In 2020, the Vaccaro group showcased the advancement of a continuous flow protocol that enabled the waste-minimized synthesis of significant pharmaceuticals and natural compounds (Scheme 58).^22^ The protocol utilizing heterogeneous manganese, octahedral-molecular sieves (K-OMS), cyclopentyl methyl ether (CPME), hydrogen peroxide, and molecular oxygen as the catalytic system could facilitate the C–H oxidative coupling of 2-aminophenols under mild reaction conditions. It is worth noting that the coupling procedure resulted in the formation of diverse 2-aminophenoxazin-3-ones in excellent yields with negligible metal contamination. In addition, a secure and environmentally benign solvent CPME was employed in this transformation, which enabled the rapid synthesis of fully functionalized molecular entities. The stability of the heterogeneous catalyst could be maintained, demonstrating minimal metal leaching and contributing to low waste generation. Particularly, the applicability of this protocol has been affirmed through its successful implementation on a multi-gram scale.

**Scheme 59:**
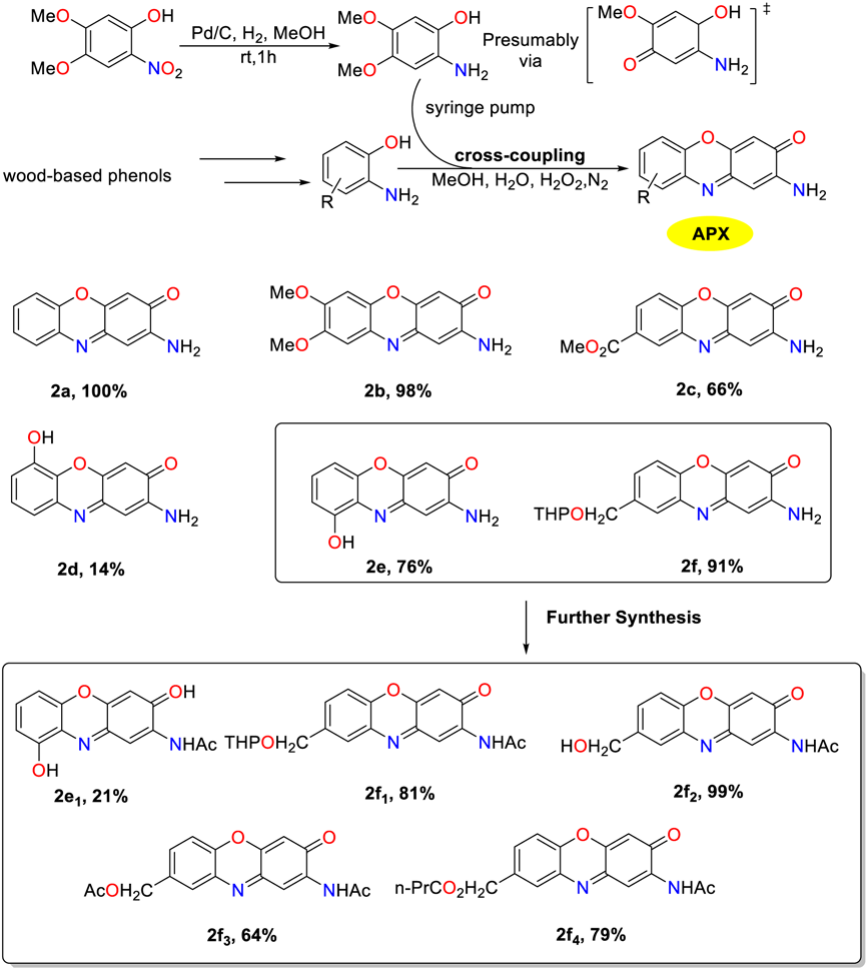
Innovative methods for the synthesis of 2-aminophenoxazinone (ref. 84).

In 2019, a novel and environmentally friendly method for the synthesis of APX compounds was developed by Opatz and colleagues utilizing xylochemicals derived from wood as the substrates through oxidative cross coupling reactions (Scheme 59).^84^ A wide range of APX derivatives with various functional groups were obtained in high yields by employing vanillin as a key precursor, enabling a concise and efficient synthesis process. Notably, the successful synthesis of APX derivatives such as questiomycin A, peristrophine, and maroxazinone was achieved, which furnished the corresponding products in moderate to good yields. Those desired products demonstrate promising low IC_50_ values, which indicate their potential utility in an antiproliferative therapy and will facilitate the evaluation of efficacy and selectivity against cancer cells.

## 5. Synthesis by innovative methods

Despite the established synthesis methods that achieved high-yield production of APX scaffolds, emerging green catalytic technologies such as photocatalysis and electrocatalysis feature the significant advantages of green environmental and sustainable characteristics. Photocatalysis harnesses light energy to drive chemical reactions and facilitates the synthesis of phenoxazinone under mild conditions, which decreases energy consumption and environmental impact. However, electrocatalysis enhances the controllability and efficiency *via* current-driven reactions, thereby improving both the economic viability and the safety of production processes. These two methodologies represent more sustainable catalytic approaches that can enhance the synthesis efficiency of phenoxazinone while adhering to the principles of green chemistry. Consequently, these synthetic strategies contribute to advancing sustainable development goals and achieving considerable promise for future applications.

**Scheme 60:**
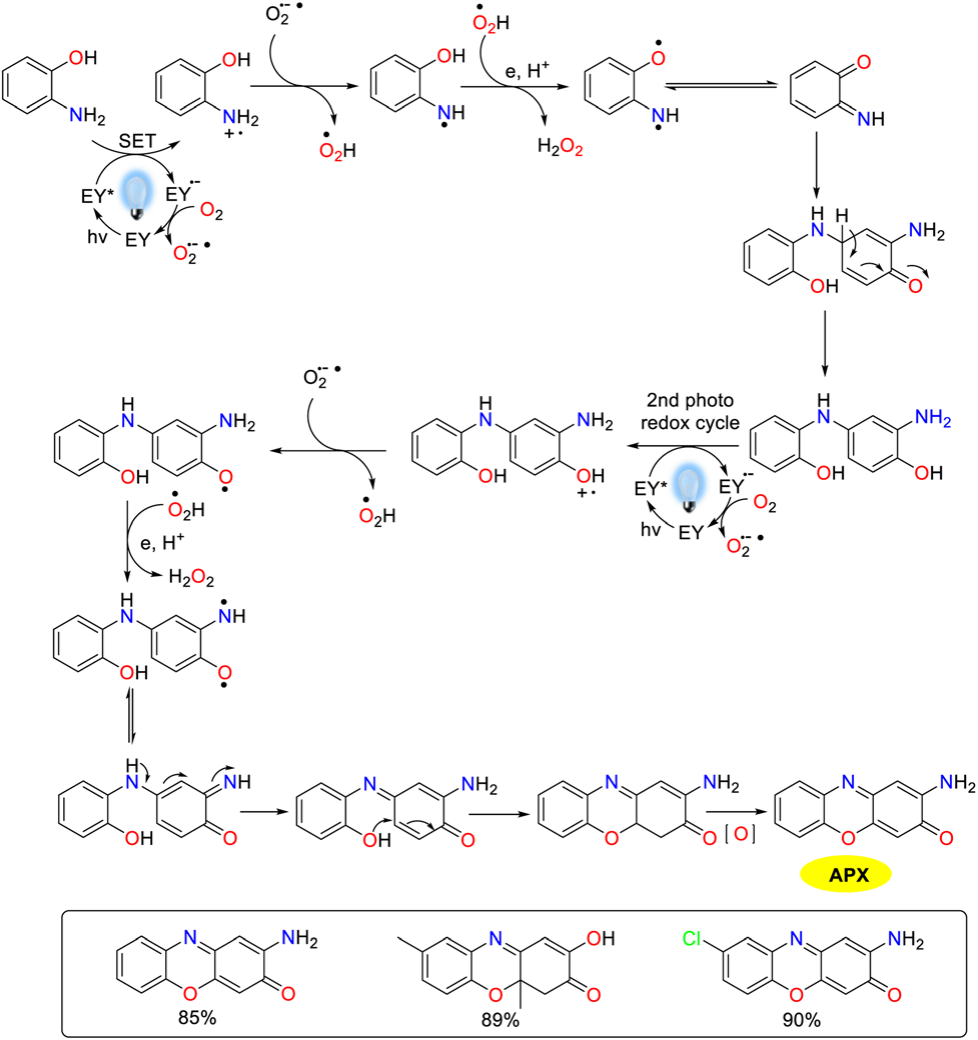
Proposed reaction pathway for the synthesis of APX from OAP (ref. 23).

In 2021, the Dhar group disclosed an efficient, economic, and metal-free photocatalytic approach for the synthesis of phenoxazinone derivatives in good yields from *ortho*-substituted aromatic amines in aqueous media at room temperature with Eosin Y as the photoredox catalyst (Scheme 60).^23^ A tentative catalytic cycle for the visible-light-induced protocol is proposed. Initially, *o*-aminophenol affords highly reactive electrophilic specie *o*-aminophenol imine cationic radical upon irradiation with blue LEDs and is then converted into an *o*-aminophenol imine radical through the reaction with a strongly basic superoxide anion. Subsequently, 1,4-conjugated Michael addition product is obtained through a single electron transfer process, followed by nucleophilic attack at the *para*-position of the quinone. Finally, the desired product is afforded through a series of sequences including the second photoredox cycle of EY, intramolecular cyclization, and oxidation process. This methodology is regarded as a considerable advancement and a valuable alternative to the existing techniques for the rational synthesis of phenoxazinone-based heterocycles.

**Scheme 61:**
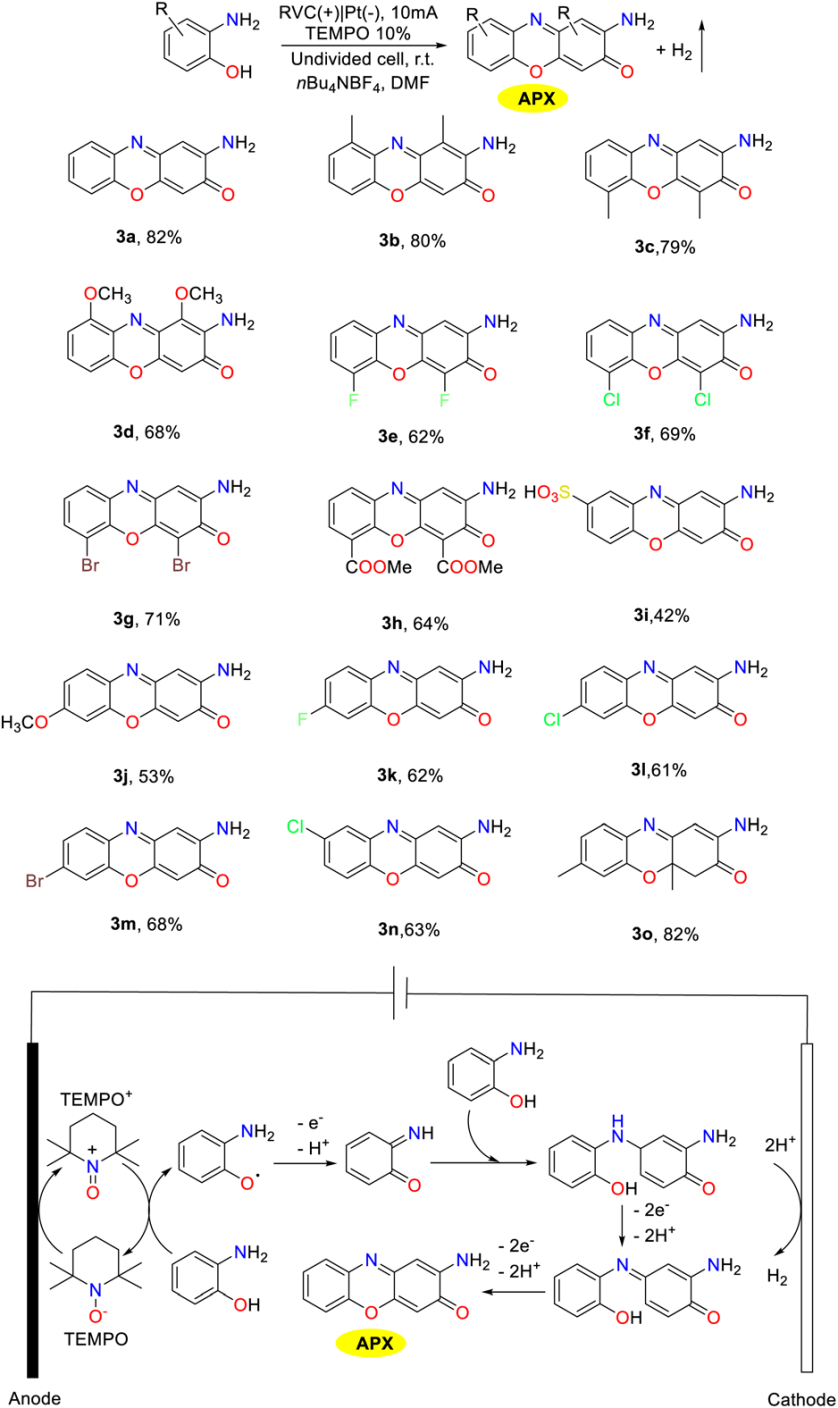
Electrochemical dehydrogenative cyclocondensation of *o*-aminophenols (ref. 21).

In 2021, a high-efficiency and straightforward TEMPO-catalyzed electrochemical methodology for the synthesis of APX scaffolds as antiproliferative agents has been disclosed by the Cai group (Scheme 61).^21^ This approach encompassed the dehydrogenative cyclocondensation of *o*-aminophenol without stoichiometric oxidants employing readily accessible TEMPO as an organo-electrocatalyst. The mechanistic study showed that the anode first oxidized TEMPO to TEMPO^+^, which reacted with OAP to form an OAP radical. The radical then lost an electron and a proton to generate the key intermediate BQMI. Next, another OAP molecule reacted with BQMI *via* 1,4-conjugate addition and oxidative dehydrogenation, followed by dehydrogenative cyclization to afford the corresponding APX. This environmentally friendly method has been demonstrated to be practical due to its application on a gram-scale and simple device. It is worth noting that a novel antitumor agent 3h featuring two carboxylic ester groups in the phenoxazinone framework could also be synthesized by the TEMPO-catalyzed electrochemical protocol. Overall, this technique offers a green and efficient avenue for the synthesis of a valuable APX skeleton, enhancing structural diversity and bioactivity in related compounds.

**Scheme 62:**
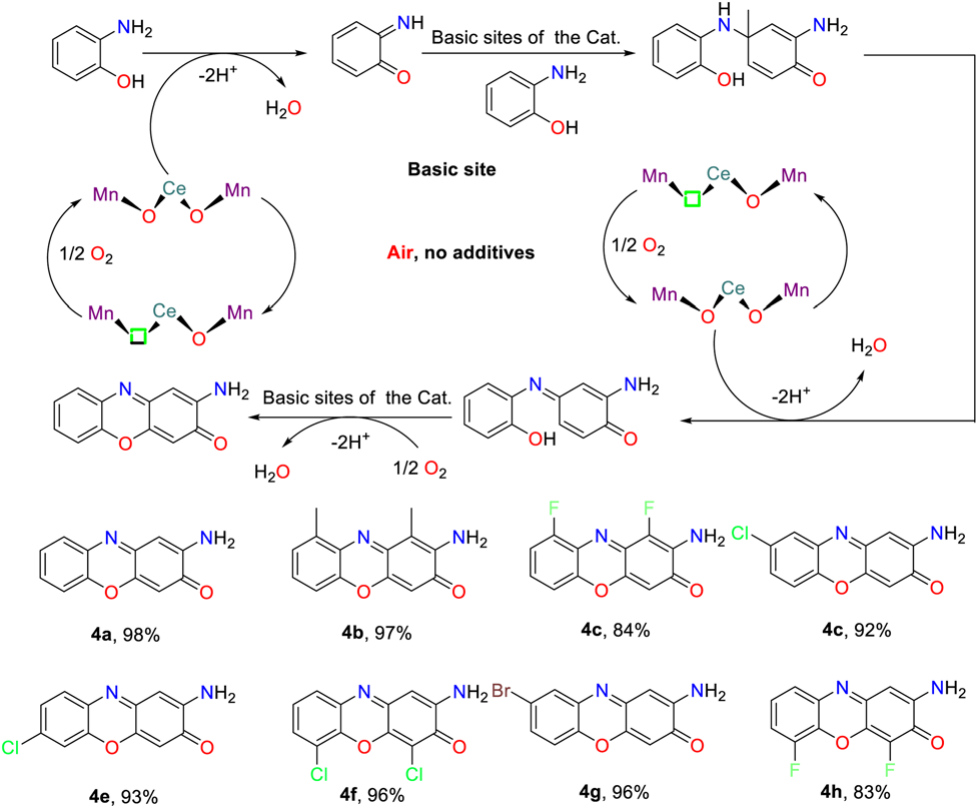
Possible reaction pathway for the synthesis of APX from OAP (ref. 85).

In 2022, the Long group accomplished the green synthesis of APX scaffolds employing cerium-doped manganese oxide as the catalyst and atmospheric oxygen as the oxidant in the absence of any additional reagent (Scheme 62).^85^ The augmented catalytic efficiency was attributed to the pronounced enhancement in the oxidation capacity of the catalyst, which was the outcome of the cerium doping process *via* the regulation of the alkalinity and activity. To assess the applicability of the Mn_0.9_Ce_0.1_O_*γ*350_ catalytic system, a great deal of substrates with electron-donating and electron-withdrawing substituents were analyzed under the optimal conditions, which led to the formation of the APX scaffolds in good yields. In particular, the molecule 4b exhibited superior fluorescence, which could be utilized as a fluorescent molecular probe for detection and labeling applications. This approach stands as a sustainable and economical strategy for the synthesis of APX in industrial applications, ensuring the stability of the heterogeneous catalyst while minimizing metal leaching.

## 6. Conclusion

In summary, elaborated molecular architectures, specifically those with 2-aminophenoxazinone motifs, represent an important class of heterocyclic compounds and remain a fascinating research area due to their pivotal skeletal units in a broad variety of alkaloid natural products, agrochemicals, pharmaceutical molecules, and functional materials. In this review, we summarized the recent advances in the synthesis of 2-aminophenoxazinone frameworks based on different catalytic approaches and synthetic process routes of 2-aminophenoxazinone compounds, most of which feature the advantages of high conversion efficiency, good functional group compatibility, and highly efficient catalytic system.

Although the current strategy allows the generation of a greater diversity of 2-aminophenoxazinone motifs with various steric and electronic natures smoothly, and open up a new platform for the exploitation of functional materials and new drugs, some formidable challenges and prominent avenues for further progress have been identified. First, the employment of transition metal complexes as the catalyst may cause the contamination of the 2-aminophenoxazinone products, which could restrict further practical application in the drug industry. Second, the great challenge of reactivity and selectivity control of those reactions, operational complexity for the formation of the transition metal complex, and competitive side reactions still remain to be addressed. A number of reliable, highly efficient, and multipurpose strategies are imminently required to further improve the chemoselectivity, regioselectivity, practicality, and simplicity, and the practical application of these approaches could be prospective to transcend in the future.

## Data availability

No primary research results, software or code have been included and no new data were generated or analysed as part of this review.

## Conflicts of interest

There are no conflicts to declare.
